# Correction: *Are sweet snacks more sensitive to price increases than sugar-sweetened beverages: analysis of British food purchase data*

**DOI:** 10.1136/bmjopen-2017-019788corr1

**Published:** 2018-05-17

Smith RD, Cornelsen L, Quirmbach D, *et al*. Are sweet snacks more sensitive to price increases than sugar-sweetened beverages: analysis of British food purchase data. *BMJ Open* 2018;**8:**e019788. doi: 10.1136/bmjopen-2017-019788

The sentence referring to bottom left panel on Figure 1 should say:

‘A price increase for chocolate and confectionary items is associated with small but significant decreases across all soft drinks (0.6–0.8% for 10% price increase) as well as biscuits (1.2%), cakes (1.6%) and savoury snacks (0.3%)’

Instead of:

‘A price increase for chocolate and confectionary items is associated with small but significant decreases across all soft drinks (0.6–0.8% for 10% price increase) as well as biscuits and cakes (1.2%), and savoury snacks (1.6%)’.

