# Supplementary material for: Are sweet snacks more sensitive to price increases than sugar-sweetened beverages: analysis of British food purchase data
Source: BMJ Open. 2018 Apr 26;8(4):e019788. doi: 10.1136/bmjopen-2017-019788 (PMC5922464; doi:10.1136/bmjopen-2017-019788)
Supplement: Supplementary file 1 [file bmjopen-2017-019788supp001.pdf]

Appendix 1: Demand modelling strategy

The demand model applied was based on the linear version of Almost Ideal Demand System where expenditure shares are modelled as a function of prices and total expenditure adjusted for all price levels:

$$w_{iht} = \alpha_i + \sum_{j=1}^{N} \gamma_{ij} lnp_{jht} + \beta_i \frac{lnx_{ht}}{P_{ht}} + \varepsilon_{iht} \quad (1)$$

Where:

w_{iht} is expenditure share of group *i* (i=1, 2, …, 13) for household *h* (h=1,2,…32,249) in 4-weekly periods *t* (t=1, 2, …, 26)

lnx_{ht} is the log of total household monthly expenditure on food and beverage per capita

lnp_{jht} is the log of price for category *j* for household *h* in period *t*

$P_{ht}$is a Laspeyres price index of geometrically weighted average prices defined as $lnP = \sum_i \bar{w}_i lnp_i$

α_i is a constant for group *i*

γ_{ij} and β_i are parameters to be estimated

ε_{iht} is a random disturbance

As not all households purchase items from each of the food and beverage groups in each period, the data includes zero-observations. These were more likely to occur in more disaggregated groups (e.g. 45% of observations among other soft drinks, 73% in cake-type snacks were zeroes). To deal with these zero observations that can bias the estimates, we followed a two-step procedure developed by Shonkweiler and Yen (1999).[1] In the first step, the decision to purchase beverages in any group was modelled as a function of lagged quantity of foods/beverages purchased in that group, household size, age of the main shopper, socio-economic group (A&B, C1&C2 or D&E), whether or not the household owns their house, income group (for the whole sample only), presence of children and time indicators to take into account seasonal trends, using a probit model. From the probit model, we estimated the probability density function (ϕ_i) and cumulative density function (Φ_i) of the predictions of the fitted model. These two variables were applied in the second step of estimating the demand function (1):

$$w_{iht}^{*} = \Phi_{iht}(w_{iht}) + \varphi_{iht}\phi_{iht} + \sum_{t=1}^{13} \rho_{it} T_{it} + v_{ih} + \varepsilon_{it} \quad (2)$$

Where:

T_{it} are indicator variables to capture any seasonal or other time effects (13 four-week periods)

v_{ih} is a fixed household effect

We estimated (2) equation-by-equation using a fixed effect model with robust clustered standard errors to allow for any misspecification, particularly serial correlation of observations within the households. Clusters were defined at the geographical area used in estimating prices (n=110).

The specification used (2) imposed the restrictions, compatible with the AIDS model, of adding-up $[\sum_{i=1}^{N} \alpha_i = 1; \sum_{i=1}^{N} \beta_i = 0]$ and homogeneity $[\sum_{i=1}^{N} \gamma_{ij} = 0]$.

There are two important sources of potential endogeneity in the model. First, total expenditure enters the model as a proxy for incomes while it is also used to calculate the expenditure shares. Furthermore, total expenditure might be endogenous because of possible correlation with unobserved characteristics affecting demand behaviour or because of shocks common to total expenditure and expenditure shares. Secondly, unit prices estimated from monthly aggregates of expenditure and volume are likely to be biased due to aggregation effects.[2] If prices or expenditures are correlated with the equation errors, estimators will be both biased and inconsistent.

To deal with quality effects in prices, we took the assumption that in a relatively small geographical area households face the same prices during the same time period. To estimate these geographical average unit values we calculated the monthly average prices for the (n=110) postcode areas which we observe in the data. Where the monthly price was missing (e.g. households did not purchase the products in this beverage group in a particular month), it was replaced by the first non-missing average of the previous and the following monthly prices.

To reduce possible endogeneity between expenditure shares (w_{iht}) and total expenditure (lnx_{ht}) that enters the demand equation in (1) we use the approach developed Blundell et al. (1999)[3] and regressed household per capita expenditure (lnx_{ht}) on household socio demographic characteristics (social class, income, income squared (whole sample only), household size and presence of children. The predicted values from the model were used as instruments for total expenditure (lnx_{ht}) in (1).

Uncompensated (Marshallian) elasticities were estimated for each beverage and food group, at sample averages (w and Φ) as follows:

$$e_{ij} = \Phi_i * \left(\frac{\gamma_{ij}}{w_i} - \frac{\beta_i w_j}{w_i}\right) - \Delta_{ij} \quad (3)$$

Where Δ_{ij} is the Kronecker delta which equals 1 when *i=j* and 0 otherwise.

Expenditure share equations in (2) are estimated with clustered (geographical area) robust standard errors and standard errors of the unconditional elasticities (3) are bootstrapped (250 replications) to account for possible bias arising from two-step procedure. Elasticities are reported with bias-corrected confidence intervals.

References

1. Shonkwiler JS, Yen ST. Two-Step Estimation of a Censored System of Equations. Am J Agr Econ. 1999 Nov 1;81(4):972–82.

2. Deaton A. Quality, quantity, and spatial variation of price. The Am Econ Rev. 1988;418–430.

3. Blundell R, Robin JM. Estimation in large and disaggregated demand systems: An estimator for conditionally linear systems. J Appl Econom. 1999;14(3):209-32.
