# Supplementary material for: Are sweet snacks more sensitive to price increases than sugar-sweetened beverages: analysis of British food purchase data
Source: BMJ Open. 2018 Apr 26;8(4):e019788. doi: 10.1136/bmjopen-2017-019788 (PMC5922464; doi:10.1136/bmjopen-2017-019788)
Supplement: Supplementary file 2 [file bmjopen-2017-019788supp002.pdf]

Appendix 2: Price elasticities

Table 1. Price elasticities of demand in full sample (n=623,459)

	High-sugar soft drinks	Medium-sugar soft drinks	Low-sugar soft drinks	Other soft drinks	Alcohol	Biscuits & cookies	Chocolate & conf.	Cake-type snacks	Savoury snacks	Meat & fish	Dairy & eggs	Fruit & vegetables	Rest food & drink
High-sugar soft drinks	**-0.77 [-0.85;-0.70]**	-0.03 [-0.06;0]	**-0.11 [-0.19;-0.02]**	-0.05 [-0.11;0.02]	**-0.19 [-0.24;-0.14]**	0.02 [-0.07;0.10]	**-0.06 [-0.09;-0.04]**	-0.02 [-0.11;0.06]	**0.22 [0.11;0.35]**	**-0.29 [-0.41;-0.15]**	**0.26 [0.18;0.34]**	-0.08 [-0.17;0.01]	0 [-0.17;0.17]
Medium-sugar soft drinks	**-0.25 [-0.44;-0.06]**	**-0.62 [-0.70;-0.55]**	**-0.28 [-0.46;-0.10]**	**-0.33 [-0.53;-0.16]**	**-0.20 [-0.33;-0.06]**	-0.19 [-0.44;0.03]	**-0.08 [-0.16;-0.02]**	-0.06 [-0.28;0.14]	**-0.34 [-0.63;-0.07]**	**1.10 [0.76;1.38]**	**-0.22 [-0.43;-0.01]**	**0.37 [0.13;0.60]**	-0.08 [-0.51;0.41]
Low-sugar soft drinks	-0.01 [-0.06;0.05]	**-0.03 [-0.05;-0.01]**	**-0.82 [-0.89;-0.76]**	**-0.13 [-0.17;-0.09]**	**-0.25 [-0.29;-0.21]**	-0.02 [-0.08;0.05]	**-0.07 [-0.09;-0.05]**	0 [-0.05;0.06]	-0.01 [-0.10;0.07]	0.05 [-0.04;0.14]	**0.15 [0.09;0.21]**	**0.12 [0.07;0.19]**	-0.04 [-0.18;0.08]
Other soft drinks	**0.11 [0.05;0.18]**	**-0.05 [-0.07;-0.02]**	0 [-0.08;0.05]	**-0.83 [-0.88;-0.77]**	**-0.17 [-0.21;-0.12]**	-0.02 [-0.10;0.05]	**-0.08 [-0.11;-0.06]**	0.01 [-0.05;0.08]	0.06 [-0.04;0.17]	**-0.17 [-0.27;-0.07]**	**0.13 [0.06;0.21]**	0.03 [-0.04;0.10]	0.01 [-0.14;0.14]
Alcohol	-0.04 [-0.10;0.00]	**-0.03 [-0.05;-0.01]**	-0.02 [-0.08;0.03]	**-0.10 [-0.14;-0.05]**	**-0.90 [-0.94;-0.86]**	**-0.06 [-0.13;-0.01]**	**0.03 [0.01;0.05]**	**0.08 [0.03;0.14]**	**0.11 [0.04;0.19]**	0.08 [-0.01;0.17]	0.02 [-0.03;0.08]	**-0.07 [-0.14;-0.01]**	**-0.37 [-0.47;-0.25]**
Biscuits & cookies	0 [-0.06;0.04]	-0.01 [-0.02;0.02]	**0.13 [0.08;0.19]**	**0.05 [0.01;0.09]**	**0.06 [0.02;0.10]**	**-0.69 [-0.75;-0.64]**	**-0.12 [-0.15;-0.09]**	**-0.07 [-0.12;-0.02]**	**0.13 [0.03;0.20]**	**-0.18 [-0.28;-0.10]**	-0.04 [-0.11;0.01]	0 [-0.06;0.07]	**-0.40 [-0.51;-0.27]**
Chocolate & conf.	**0.08 [0.02;0.15]**	0.02 [-0.01;0.04]	**0.16 [0.09;0.24]**	0.01 [-0.04;0.07]	**0.07 [0.01;0.12]**	**-0.17 [-0.26;-0.10]**	**-0.74 [-0.78;-0.71]**	**0.07 [0.01;0.13]**	**0.27 [0.17;0.39]**	**0.44 [0.30;0.54]**	**-0.30 [-0.38;-0.22]**	**-0.26 [-0.35;-0.19]**	**-0.94 [-1.10;-0.77]**
Cake-type snacks	-0.02 [-0.11;0.06]	0 [-0.03;0.03]	**0.17 [0.08;0.27]**	0.09 [0;0.16]	-0.02 [-0.09;0.05]	**-0.23 [-0.33;-0.13]**	**-0.16 [-0.19;-0.11]**	**-0.66 [-0.78;-0.57]**	**0.31 [0.14;0.44]**	**-0.32 [-0.45;-0.14]**	0.06 [-0.04;0.17]	-0.08 [-0.17;0.02]	-0.08 [-0.17;0.02]
Savoury snacks	0 [-0.05;0.04]	0.01 [0;0.03]	0 [-0.05;0.05]	-0.04 [-0.07;0]	-0.03 [-0.06;0]	-0.02 [-0.07;0.04]	**-0.03 [-0.05;-0.01]**	-0.04 [-0.10;0.01]	**-0.75 [-0.82;-0.67]**	-0.03 [-0.12;0.05]	0 [-0.05;0.05]	0 [-0.05;0.05]	**-0.23 [-0.32;-0.11]**
Meat & fish	0 [-0.03;0.03]	0 [-0.01;0.01]	0.01 [-0.03;0.04]	0.01 [-0.01;0.04]	0.03 [0;0.05]	**-0.05 [-0.09;-0.01]**	0 [-0.01;0.02]	-0.02 [-0.05;0]	-0.04 [-0.10;0.01]	**-0.76 [-0.81;-0.70]**	**-0.12 [-0.15;-0.08]**	**-0.08 [-0.12;-0.04]**	-0.02 [-0.08;0.06]
Dairy & eggs	**0.03 [0.01;0.05]**	-0.01 [-0.02;0]	0.01 [-0.01;0.03]	**0.04 [0.02;0.06]**	0.01 [-0.01;0.02]	0.02 [0;0.05]	-0.01 [-0.03;0]	-0.01 [-0.04;0.01]	-0.02 [-0.06;0.02]	-0.03 [-0.06;0.02]	**-0.88 [-0.91;-0.85]**	**-0.12 [-0.14;-0.09]**	**-0.09 [-0.14;-0.03]**
Fruit & veg	-0.01 [-0.03;0.02]	0.00 [-0.01;0.01]	0 [-0.03;0.02]	-0.01 [-0.03;0]	-0.02 [-0.04;0.01]	0.03 [0;0.06]	**-0.07 [-0.09;-0.06]**	**-0.08 [-0.11;-0.06]**	**0.07 [0.03;0.12]**	**-0.10 [-0.14;-0.05]**	-0.01 [-0.04;0.02]	**-0.60 [-0.63;-0.57]**	-0.06 [-0.13;0.01]
Rest food & drink	-0.01 [-0.04;0.01]	0 [-0.01;0.01]	**-0.03 [-0.05;-0.01]**	**0.04 [0.02;0.06]**	0 [-0.01;0.02]	**0.04 [0.01;0.06]**	**0.03 [0.02;0.04]**	0 [-0.02;0.02]	**-0.13 [-0.16;-0.03]**	**-0.1 [-0.14;-0.06]**	-0.01 [-0.04;0.02]	-0.03 [-0.06;0]	**-0.66 [-0.71;-0.61]**

Notes: Elasticities in bold indicate those where 95% confidence intervals do not include zero. Columns indicate the group of price change and rows indicate the group of demand change. High-sugar soft drinks: >8g of sugar/100ml; medium-sugar soft drinks: 5-8g of sugar/100ml; low-sugar soft drinks: <5g of sugar/100ml; other soft drinks: water, fruit juice with no added sugars and milk-based drinks.

Table 2. Price elasticities of demand in low-income (annual household income £<20,000) sample (n=223,174)

	High-sugar soft drinks	Medium-sugar soft drinks	Low-sugar soft drinks	Other soft drinks	Alcohol	Biscuits & cookies	Chocolate & conf.	Cake-type snacks	Savoury snacks	Meat & fish	Dairy & eggs	Fruit & vegetables	Rest food & drink
High-sugar soft drinks	**-0.84**<br>**[-1.00;-0.71]**	-0.03<br>[-0.09;0.02]	**-0.15**<br>**[-0.27;-0.01]**	0.00<br>[-0.11;0.12]	**-0.25**<br>**[-0.36;-0.16]**	0.02<br>[-0.10;0.19]	-0.06<br>[-0.11;0]	-0.07<br>[-0.24;0.11]	0.17<br>[0;0.38]	-0.02<br>[-0.28;0.21]	**0.30**<br>**[0.15;0.44]**	-0.07<br>[-0.24;0.10]	-0.08<br>[-0.43;0.23]
Medium-sugar soft drinks	**-0.33**<br>**[-0.68;-0.02]**	**-0.57**<br>**[-0.69;-0.44]**	-0.05<br>[-0.38;0.26]	**-0.31**<br>**[-0.62;-0.07]**	-0.25<br>[-0.46;0]	-0.08<br>[-0.49;0.29]	-0.10<br>[-0.23;0.01]	-0.17<br>[-0.64;0.22]	-0.39<br>[-0.89;0.31]	**0.98**<br>**[0.39;1.46]**	**-0.39**<br>**[-0.71;-0.03]**	**0.43**<br>**[0.02;0.83]**	-0.06<br>[-0.87;0.73]
Low-sugar soft drinks	0<br>[-0.10;0.09]	-0.02<br>[-0.05;0.02]	**-0.80**<br>**[-0.93;-0.70]**	**-0.13**<br>**[-0.22;-0.05]**	**-0.28**<br>**[-0.35;-0.21]**	-0.01<br>[-0.13;0.10]	**-0.07**<br>**[-0.10;-0.03]**	-0.03<br>[-0.15;0.06]	0.05<br>[-0.18;0.17]	0.03<br>[-0.16;0.21]	**0.14**<br>**[0.03;0.25]**	**0.21**<br>**[0.10;0.33]**	-0.14<br>[-0.44;0.06]
Other soft drinks	0.08<br>[-0.04;0.23]	-0.01<br>[-0.05;0.05]	**0.12**<br>**[0.01;0.25]**	**-0.89**<br>**[-0.98;-0.80]**	**-0.27**<br>**[-0.37;-0.17]**	-0.08<br>[-0.22;0.08]	-0.06<br>[-0.11;-0.02]	0<br>[-0.12;0.13]	0.09<br>[-0.08;0.27]	**-0.27**<br>**[-0.51;-0.07]**	**0.19**<br>**[0.05;0.32]**	**0.26**<br>**[0.11;0.38]**	-0.17<br>[-0.41;0.06]
Alcohol	-0.01<br>[-0.12;0.09]	-0.04<br>[-0.08;0]	0.01<br>[-0.06;0.11]	-0.05<br>[-0.14;0.02]	**-0.92**<br>**[-0.98;-0.85]**	-0.04<br>[-0.15;0.06]	0.02<br>[-0.01;0.06]	-0.04<br>[-0.13;0.06]	**0.17**<br>**[0.05;0.33]**	0.15<br>[-0.02;0.30]	0.12<br>[-0.01;0.21]	-0.08<br>[-0.17;0.06]	**-0.50**<br>**[-0.69;-0.28]**
Biscuits & cookies	0.01<br>[-0.09;0.10]	-0.03<br>[-0.06;0]	0.07<br>[0;0.16]	0.04<br>[-0.03;0.11]	0<br>[-0.06;0.06]	**-0.74**<br>**[-0.85;-0.65]**	**-0.13**<br>**[-0.17;-0.09]**	-0.01<br>[-0.11;0.10]	0.12<br>[-0.02;0.26]	-0.16<br>[-0.30;0]	-0.03<br>[-0.12;0.07]	0.04<br>[-0.07;0.14]	**-0.34**<br>**[-0.60;-0.18]**
Chocolate & conf.	0.08<br>[-0.03;0.19]	0.01<br>[-0.03;0.04]	**0.16**<br>**[0.04;0.25]**	0.02<br>[-0.06;0.10]	0.07<br>[0;0.16]	**-0.27**<br>**[-0.40;-0.15]**	**-0.73**<br>**[-0.79;-0.67]**	0.02<br>[-0.09;0.15]	**0.19**<br>**[0.02;0.35]**	**0.49**<br>**[0.31;0.67]**	**-0.29**<br>**[-0.42;-0.16]**	**-0.35**<br>**[-0.46;-0.21]**	**-0.74**<br>**[-1.00;-0.48]**
Cake-type snacks	-0.12<br>[-0.27;0.04]	-0.04<br>[-0.10;0.02]	**0.24**<br>**[0.09;0.39]**	**0.14**<br>**[0.03;0.27]**	-0.09<br>[-0.20;0.01]	-0.19<br>[-0.35;0.02]	**-0.15**<br>**[-0.23;-0.09]**	**-0.71**<br>**[-0.85;-0.54]**	**0.27**<br>**[0.06;0.47]**	**-0.38**<br>**[-0.68;-0.11]**	0.10<br>[-0.05;0.29]	-0.07<br>[-0.23;0.11]	**-0.44**<br>**[-0.83;-0.12]**
Savoury snacks	0.02<br>[-0.07;0.09]	0.02<br>[-0.01;0.05]	-0.02<br>[-0.10;0.06]	0.02<br>[-0.05;0.08]	-0.03<br>[-0.10;0.02]	-0.02<br>[-0.11;0.07]	-0.03<br>[-0.08;0]	-0.07<br>[-0.16;0]	**-0.71**<br>**[-0.83;-0.59]**	0<br>[-0.14;0.12]	-0.02<br>[-0.10;0.07]	-0.02<br>[-0.10;0.07]	**-0.27**<br>**[-0.48;-0.09]**
Meat & fish	0.02<br>[-0.04;0.07]	0<br>[-0.02;0.02]	-0.01<br>[-0.06;0.04]	0.03<br>[-0.02;0.07]	**0.05**<br>**[0.01;0.09]**	**-0.07**<br>**[-0.13;-0.01]**	0.02<br>[0;0.05]	-0.01<br>[-0.06;0.06]	0.01<br>[-0.06;0.10]	**-0.80**<br>**[-0.90;-0.70]**	**-0.15**<br>**[-0.21;-0.08]**	**-0.11**<br>**[-0.17;-0.04]**	-0.10<br>[-0.22;0.02]
Dairy & eggs	0.02<br>[-0.01;0.06]	-0.01<br>[-0.02;0.01]	-0.01<br>[-0.05;0.03]	0.03<br>[0;0.07]	0.02<br>[-0.01;0.05]	0.02<br>[-0.02;0.07]	-0.02<br>[-0.04;0.01]	0.01<br>[-0.03;0.06]	-0.05<br>[-0.10;0.02]	-0.07<br>[-0.13;0]	**-0.86**<br>**[-0.90;-0.81]**	**-0.12**<br>**[-0.17;-0.08]**	-0.05<br>[-0.15;0.05]
Fruit & veg	-0.03<br>[-0.08;0.01]	0<br>[-0.02;0.02]	0.02<br>[-0.02;0.07]	-0.03<br>[-0.06;0.01]	-0.02<br>[-0.06;0.02]	0.01<br>[-0.04;0.07]	**-0.09**<br>**[-0.12;-0.07]**	**-0.08**<br>**[-0.13;-0.04]**	**0.13**<br>**[0.05;0.20]**	-0.08<br>[-0.16;0]	-0.03<br>[-0.09;0.02]	**-0.58**<br>**[-0.64;-0.53]**	**-0.15**<br>**[-0.26;-0.04]**
Rest food & drink	-0.01<br>[-0.04;0.04]	0.01<br>[-0.01;0.02]	**-0.05**<br>**[-0.1;-0.01]**	0.01<br>[-0.02;0.05]	0.02<br>[0;0.05]	**0.06**<br>**[0.01;0.10]**	**0.03**<br>**[0.01;0.05]**	**0.05**<br>**[0.01;0.09]**	-0.18<br>[-0.24;0]	**-0.12**<br>**[-0.18;-0.06]**	-0.04<br>[-0.08;0.01]	-0.03<br>[-0.08;0.01]	**-0.57**<br>**[-0.66;-0.48]**

Notes: Elasticities in bold indicate those where 95% confidence intervals do not include zero. Columns indicate the group of price change and rows indicate the group of demand change. High-sugar soft drinks: >8g of sugar/100ml; medium-sugar soft drinks: 5-8g of sugar/100ml; low-sugar soft drinks: <5g of sugar/100ml; other soft drinks: water, fruit juice with no added sugars and milk-based drinks.

Table 3. Price elasticities of demand in mid-income (annual household income £20,000-£49,000) sample (n=305,841)

	High-sugar soft drinks	Medium-sugar soft drinks	Low-sugar soft drinks	Other soft drinks	Alcohol	Biscuits & cookies	Chocolate & conf.	Cake-type snacks	Savoury snacks	Meat & fish	Dairy & eggs	Fruit & vegetables	Rest food & drink
High-sugar soft drinks	**-0.75**<br>**[-0.85;-0.64]**	**-0.04**<br>**[-0.09;-0.01]**	-0.06<br>[-0.16;0.04]	-0.07<br>[-0.17;0.02]	**-0.15**<br>**[-0.22;-0.06]**	0.05<br>[-0.07;0.18]	**-0.07**<br>**[-0.11;-0.02]**	-0.02<br>[-0.14;0.11]	**0.27**<br>**[0.10;0.42]**	**-0.36**<br>**[-0.53;-0.17]**	**0.20**<br>**[0.08;0.33]**	-0.10<br>[-0.22;0.03]	0.01<br>[-0.27;0.22]
Medium-sugar soft drinks	-0.20<br>[-0.43;0.05]	**-0.67**<br>**[-0.78;-0.57]**	**-0.51**<br>**[-0.74;-0.23]**	**-0.36**<br>**[-0.60;-0.15]**	-0.12<br>[-0.30;0.13]	-0.06<br>[-0.40;0.27]	**-0.10**<br>**[-0.20;-0.01]**	0.00<br>[-0.27;0.27]	**-0.48**<br>**[-0.94;-0.11]**	**1.24**<br>**[0.72;1.69]**	-0.19<br>[-0.44;0.08]	**0.41**<br>**[0.10;0.76]**	-0.06<br>[-0.77;0.55]
Low-sugar soft drinks	-0.01<br>[-0.08;0.06]	**-0.05**<br>**[-0.08;-0.02]**	**-0.85**<br>**[-0.92;-0.77]**	**-0.09**<br>**[-0.14;-0.02]**	**-0.23**<br>**[-0.28;-0.18]**	-0.01<br>[-0.09;0.07]	**-0.07**<br>**[-0.12;-0.04]**	0.00<br>[-0.08;0.10]	-0.07<br>[-0.20;0.04]	**0.13**<br>**[0.01;0.25]**	**0.16**<br>**[0.08;0.26]**	0.07<br>[-0.02;0.15]	-0.01<br>[-0.14;0.17]
Other soft drinks	**0.11**<br>**[0.03;0.22]**	**-0.06**<br>**[-0.10;-0.03]**	0.02<br>[-0.07;0.12]	**-0.79**<br>**[-0.87;-0.73]**	**-0.13**<br>**[-0.19;-0.07]**	0.03<br>[-0.08;0.14]	**-0.07**<br>**[-0.11;-0.04]**	-0.01<br>[-0.11;0.09]	0.03<br>[-0.10;0.18]	-0.13<br>[-0.25;0.06]	0.13<br>[0;0.22]	-0.04<br>[-0.14;0.06]	-0.02<br>[-0.23;0.20]
Alcohol	-0.04<br>[-0.12;0.03]	-0.02<br>[-0.05;0]	0.01<br>[-0.05;0.09]	**-0.13**<br>**[-0.20;-0.08]**	**-0.91**<br>**[-0.95;-0.85]**	-0.05<br>[-0.15;0.03]	0.03<br>[0;0.05]	**0.13**<br>**[0.05;0.21]**	**0.13**<br>**[0.01;0.23]**	0.04<br>[-0.08;0.17]	-0.04<br>[-0.11;0.04]	**-0.10**<br>**[-0.18;-0.02]**	**-0.29**<br>**[-0.46;-0.14]**
Biscuits & cookies	-0.01<br>[-0.08;0.07]	0<br>[-0.02;0.03]	**0.18**<br>**[0.11;0.25]**	0.05<br>[0;0.11]	**0.09**<br>**[0.02;0.13]**	**-0.67**<br>**[-0.75;-0.58]**	**-0.11**<br>**[-0.15;-0.08]**	**-0.11**<br>**[-0.18;-0.05]**	**0.14**<br>**[0.03;0.24]**	**-0.19**<br>**[-0.33;-0.08]**	-0.06<br>[-0.16;0.01]	-0.01<br>[-0.09;0.08]	**-0.45**<br>**[-0.62;-0.29]**
Chocolate & conf.	0.06<br>[-0.05;0.17]	0.01<br>[-0.02;0.04]	**0.21**<br>**[0.12;0.32]**	0.02<br>[-0.07;0.09]	0.07<br>[0;0.14]	-0.07<br>[-0.18;0.03]	**-0.74**<br>**[-0.79;-0.69]**	0.07<br>[-0.02;0.19]	**0.27**<br>**[0.12;0.42]**	**0.41**<br>**[0.24;0.58]**	**-0.30**<br>**[-0.42;-0.19]**	**-0.20**<br>**[-0.31;-0.09]**	**-1.03**<br>**[-1.32;-0.81]**
Cake-type snacks	0.12<br>[-0.01;0.26]	0.05<br>[0;0.09]	**0.15**<br>**[0.03;0.26]**	0.06<br>[-0.05;0.17]	0.01<br>[-0.09;0.09]	**-0.26**<br>**[-0.47;-0.13]**	**-0.16**<br>**[-0.22;-0.11]**	**-0.65**<br>**[-0.78;-0.49]**	**0.25**<br>**[0.08;0.45]**	**-0.24**<br>**[-0.42;-0.04]**	-0.02<br>[-0.17;0.11]	**-0.16**<br>**[-0.31;-0.01]**	**-0.55**<br>**[-0.82;-0.23]**
Savoury snacks	-0.02<br>[-0.08;0.03]	0.01<br>[-0.01;0.04]	0.03<br>[-0.04;0.08]	**-0.06**<br>**[-0.11;-0.01]**	-0.02<br>[-0.08;0.02]	-0.01<br>[-0.10;0.05]	-0.02<br>[-0.05;0.02]	-0.02<br>[-0.11;0.04]	**-0.75**<br>**[-0.84;-0.65]**	-0.05<br>[-0.16;0.05]	-0.01<br>[-0.09;0.07]	-0.01<br>[-0.09;0.07]	**-0.23**<br>**[-0.35;-0.10]**
Meat & fish	-0.01<br>[-0.05;0.03]	0.01<br>[0;0.03]	0.03<br>[-0.02;0.07]	0.01<br>[-0.03;0.04]	0.03<br>[0;0.06]	-0.05<br>[-0.10;0]	0<br>[-0.02;0.02]	**-0.05**<br>**[-0.11;-0.01]**	-0.06<br>[-0.14;0]	**-0.75**<br>**[-0.81;-0.67]**	**-0.12**<br>**[-0.17;-0.07]**	**-0.08**<br>**[-0.13;-0.02]**	0.04<br>[-0.05;0.14]
Dairy & eggs	0.03<br>[-0.01;0.06]	-0.01<br>[-0.02;0]	0.03<br>[-0.01;0.07]	**0.04**<br>**[0.01;0.06]**	0<br>[-0.02;0.03]	0.03<br>[-0.01;0.07]	-0.01<br>[-0.03;0.01]	-0.03<br>[-0.07;0.01]	-0.02<br>[-0.09;0.03]	-0.01<br>[-0.07;0.04]	**-0.89**<br>**[-0.94;-0.84]**	**-0.13**<br>**[-0.16;-0.08]**	**-0.09**<br>**[-0.19;-0.01]**
Fruit & veg	0.01<br>[-0.02;0.05]	0.01<br>[0;0.02]	-0.02<br>[-0.05;0.02]	-0.03<br>[-0.05;0]	0<br>[-0.03;0.02]	0.02<br>[-0.02;0.06]	**-0.06**<br>**[-0.08;-0.04]**	**-0.08**<br>**[-0.12;-0.05]**	0.05<br>[0;0.11]	**-0.12**<br>**[-0.18;-0.05]**	0.02<br>[-0.02;0.06]	**-0.58**<br>**[-0.62;-0.54]**	-0.04<br>[-0.12;0.04]
Rest food & drink	0<br>[-0.05;0.05]	0<br>[-0.01;0.02]	**-0.05**<br>**[-0.09;-0.03]**	**0.05**<br>**[0.03;0.08]**	-0.02<br>[-0.04;0.01]	0.01<br>[-0.03;0.05]	**0.03**<br>**[0.01;0.04]**	0<br>[-0.04;0.03]	**-0.11**<br>**[-0.15;-0.03]**	**-0.09**<br>**[-0.14;-0.04]**	0.02<br>[-0.01;0.06]	-0.02<br>[-0.05;0.01]	**-0.7**<br>**[-0.76; -0.63]**

Notes: Elasticities in bold indicate those where 95% confidence intervals do not include zero. Columns indicate the group of price change and rows indicate the group of demand change. High-sugar soft drinks: >8g of sugar/100ml; medium-sugar soft drinks: 5-8g of sugar/100ml; low-sugar soft drinks: <5g of sugar/100ml; other soft drinks: water, fruit juice with no added sugars and milk-based drinks.

Table 4. Price elasticities of demand in high-income (annual household income > £50,000) sample (n=94,444)

	High-sugar soft drinks	Medium-sugar soft drinks	Low-sugar soft drinks	Other soft drinks	Alcohol	Biscuits & cookies	Chocolate & conf.	Cake-type snacks	Savoury snacks	Meat & fish	Dairy & eggs	Fruit & veg	Rest food & drink
High-sugar soft drinks	**-0.60**<br>**[-0.76;-0.39]**	-0.02<br>[-0.10;0.05]	**-0.26**<br>**[-0.46;-0.06]**	-0.03<br>[-0.19;0.12]	**-0.18**<br>**[-0.30;-0.04]**	0.03<br>[-0.18;0.33]	**-0.08**<br>**[-0.14;-0.01]**	0.06<br>[-0.15;0.27]	**0.32**<br>**[0.03;0.58]**	**-0.73**<br>**[-1.05;-0.34]**	**0.31**<br>**[0.14;0.55]**	-0.05<br>[-0.27;0.16]	0.19<br>[-0.34;0.60]
Medium-sugar soft drinks	-0.19<br>[-0.63;0.40]	**-0.57**<br>**[-0.72;-0.34]**	0.01<br>[-0.42;0.46]	**-0.39**<br>**[-0.76;-0.02]**	-0.26<br>[-0.66;0.03]	**-0.75**<br>**[-1.23;-0.13]**	0.01<br>[-0.15;0.17]	-0.16<br>[-0.65;0.34]	-0.08<br>[-0.75;0.59]	**0.90**<br>**[0.24;1.70]**	0.29<br>[-0.14;0.88]	0.12<br>[-0.43;0.72]	0.09<br>[-1.19;0.91]
Low-sugar soft drinks	-0.01<br>[-0.13;0.11]	-0.01<br>[-0.07;0.03]	**-0.78**<br>**[-0.94;-0.64]**	**-0.23**<br>**[-0.33;-0.11]**	**-0.22**<br>**[-0.29;-0.11]**	-0.06<br>[-0.21;0.07]	**-0.05**<br>**[-0.11;-0.01]**	0.07<br>[-0.07;0.22]	0.05<br>[-0.15;0.23]	-0.15<br>[-0.43;0.07]	0.13<br>[-0.02;0.27]	**0.16**<br>**[0.02;0.30]**	0.14<br>[-0.15;0.41]
Other soft drinks	**0.13**<br>**[0.02;0.28]**	**-0.06**<br>**[-0.11;-0.02]**	-0.13<br>[-0.28;0.02]	**-0.83**<br>**[-0.96;-0.71]**	-0.09<br>[-0.20;0.01]	-0.02<br>[-0.17;0.15]	**-0.13**<br>**[-0.21;-0.07]**	0.09<br>[-0.05;0.25]	0.12<br>[-0.08;0.36]	-0.19<br>[-0.45;0.08]	0.01<br>[-0.14;0.18]	-0.11<br>[-0.27;0.11]	0.29<br>[-0.04;0.58]
Alcohol	-0.10<br>[-0.21;0.05]	-0.04<br>[-0.09;0]	-0.07<br>[-0.21;0.07]	-0.08<br>[-0.19;0.01]	**-0.82**<br>**[-0.93;-0.74]**	-0.12<br>[-0.29;0.03]	0.04<br>[0;0.09]	0.11<br>[-0.05;0.25]	0.01<br>[-0.18;0.22]	0.11<br>[-0.10;0.30]	0.00<br>[-0.15;0.15]	-0.01<br>[-0.17;0.12]	-0.33<br>[-0.61;0]
Biscuits & cookies	0<br>[-0.14;0.12]	0.02<br>[-0.02;0.06]	**0.15**<br>**[0.03;0.28]**	0.06<br>[-0.05;0.17]	0.06<br>[-0.03;0.15]	**-0.64**<br>**[-0.78;-0.50]**	**-0.11**<br>**[-0.16;-0.04]**	-0.07<br>[-0.23;0.07]	0.13<br>[-0.09;0.31]	**-0.19**<br>**[-0.43;-0.01]**	0.02<br>[-0.10;0.20]	-0.12<br>[-0.26;0.08]	-0.35<br>[-0.62;0.01]
Chocolate & conf.	**0.19**<br>**[0.01;0.36]**	0.05<br>[-0.02;0.13]	-0.01<br>[-0.19;0.23]	-0.01<br>[-0.15;0.15]	0.13<br>[0;0.27]	-0.18<br>[-0.39;0.01]	**-0.75**<br>**[-0.85;-0.66]**	**0.27**<br>**[0.06;0.42]**	**0.49**<br>**[0.24;0.72]**	**0.41**<br>**[0.11;0.73]**	**-0.30**<br>**[-0.48;-0.08]**	**-0.22**<br>**[-0.44;-0.03]**	**-1.10**<br>**[-1.44;-0.72]**
Cake-type snacks	**-0.22**<br>**[-0.50;-0.01]**	-0.08<br>[-0.16;0.02]	0.12<br>[-0.08;0.37]	-0.02<br>[-0.19;0.18]	0.12<br>[-0.05;0.30]	-0.25<br>[-0.52;0.02]	**-0.14**<br>**[-0.23;-0.04]**	**-0.53**<br>**[-0.74;-0.25]**	**0.70**<br>**[0.30;1.03]**	-0.24<br>[-0.63;0.20]	0.25<br>[-0.06;0.51]	0.04<br>[-0.23;0.32]	**-1.18**<br>**[-1.71;-0.60]**
Savoury snacks	0.03<br>[-0.07;0.16]	0.01<br>[-0.04;0.04]	-0.03<br>[-0.15;0.06]	-0.07<br>[-0.15;0.04]	-0.03<br>[-0.11;0.05]	-0.04<br>[-0.17;0.09]	**-0.07**<br>**[-0.13;-0.01]**	-0.05<br>[-0.15;0.07]	**-0.83**<br>**[-0.98;-0.68]**	-0.05<br>[-0.25;0.15]	0.08<br>[-0.06;0.21]	0.07<br>[-0.06;0.21]	-0.14<br>[-0.36;0.11]
Meat & fish	-0.01<br>[-0.10;0.06]	0<br>[-0.03;0.02]	0.01<br>[-0.07;0.09]	0<br>[-0.07;0.05]	-0.01<br>[-0.07;0.04]	0<br>[-0.07;0.08]	-0.01<br>[-0.04;0.03]	0.01<br>[-0.08;0.07]	-0.11<br>[-0.24;0.01]	**-0.70**<br>**[-0.81;-0.57]**	-0.06<br>[-0.14;0.03]	-0.04<br>[-0.12;0.05]	-0.02<br>[-0.17;0.14]
Dairy & eggs	0.02<br>[-0.05;0.07]	0<br>[-0.02;0.02]	0.01<br>[-0.04;0.06]	0.04<br>[-0.01;0.10]	-0.02<br>[-0.06;0.03]	-0.01<br>[-0.07;0.06]	-0.03<br>[-0.06;0]	0<br>[-0.05;0.08]	-0.02<br>[-0.12;0.08]	0.02<br>[-0.06;0.12]	**-0.93**<br>**[-1.01;-0.86]**	**-0.09**<br>**[-0.17;-0.03]**	**-0.23**<br>**[-0.39;-0.06]**
Fruit & veg	0<br>[-0.06;0.07]	-0.02<br>[-0.04;0]	0.01<br>[-0.06;0.07]	0.04<br>[-0.01;0.09]	-0.03<br>[-0.07;0.01]	**0.09**<br>**[0.02;0.15]**	**-0.06**<br>**[-0.10;-0.03]**	**-0.08**<br>**[-0.15;-0.01]**	0.03<br>[-0.06;0.12]	-0.08<br>[-0.19;0.03]	-0.03<br>[-0.11;0.06]	**-0.69**<br>**[-0.75;-0.58]**	0.07<br>[-0.12;0.21]
Rest food & drink	-0.02<br>[-0.05;0.09]	0.01<br>[-0.01;0.03]	0.01<br>[-0.04;0.07]	0.03<br>[-0.01;0.07]	-0.01<br>[-0.05;0.02]	0.05<br>[-0.02;0.11]	0.04<br>[0;0.06]	**-0.06**<br>**[-0.12;-0.01]**	**-0.1**<br>**[-0.18;-0.01]**	**-0.1**<br>**[-0.19;-0.01]**	-0.02<br>[-0.08;0.05]	-0.06<br>[-0.13;0]	**-0.7**<br>**[-0.83;-0.58]**

Notes: Elasticities in bold indicate those where 95% confidence intervals do not include zero. Columns indicate the group of price change and rows indicate the group of demand change. High-sugar soft drinks: >8g of sugar/100ml; medium-sugar soft drinks: 5-8g of sugar/100ml; low-sugar soft drinks: <5g of sugar/100ml; other soft drinks: water, fruit juice with no added sugars and milk-based drinks.
