# Supplementary material for: Are sweet snacks more sensitive to price increases than sugar-sweetened beverages: analysis of British food purchase data
Source: BMJ Open. 2018 Apr 26;8(4):e019788. doi: 10.1136/bmjopen-2017-019788 (PMC5922464; doi:10.1136/bmjopen-2017-019788)
Supplement: Author's manuscript [file bmjopen-2017-019788.draft_revisions.pdf]

BMJ Open

BMJ Open is committed to open peer review. As part of this commitment we make the peer review history of every article we publish publicly available.

When an article is published we post the peer reviewers' comments and the authors' responses online. We also post the versions of the paper that were used during peer review. These are the versions that the peer review comments apply to.

The versions of the paper that follow are the versions that were submitted during the peer review process. They are not the versions of record or the final published versions. They should not be cited or distributed as the published version of this manuscript.

BMJ Open is an open access journal and the full, final, typeset and author-corrected version of record of the manuscript is available on our site with no access controls, subscription charges or pay-per-view fees (http://bmjopen.bmj.com).

If you have any questions on BMJ Open's open peer review process please email editorial.bmjopen@bmj.com

BMJ Open

Reducing sugar consumption: are sweet snacks more sensitive to price increases than sugar-sweetened beverages?

Journal:	*BMJ Open*
Manuscript ID	bmjopen-2017-019788
Article Type:	Research
Date Submitted by the Author:	26-Sep-2017
Complete List of Authors:	Smith, Richard ; London School of Hygiene and Tropical Medicine, Faculty of Public Health and Policy<br>Cornelsen, Laura; London School of Hygiene and Tropical Medicine,<br>Quirmbach, Diana<br>Jebb, Susan; Univerof Oxford, Primary Care Health Sciences<br>Marteau, Theresa; University of Cambridge, Behaviour and Health Research Unit
**Primary Subject Heading**:	Health economics
Secondary Subject Heading:	Public health
Keywords:	price elasticity, sweet snacks, sugar-sweetened beverages, fiscal policy

SCHOLARONE™
Manuscripts

For peer review only - http://bmjopen.bmj.com/site/about/guidelines.xhtml

Reducing sugar consumption: are sweet snacks more sensitive to price increases than sugar-sweetened beverages?

Richard D Smith (PhD) [1,2]*

Laura Cornelsen (PhD) [1]

Diana Quirmbach (PhD) [1, 2]

Susan A. Jebb (PhD) [2,3]

Theresa Marteau (PhD) [2]

[1] Faculty of Public Health and Policy, London School of Hygiene and Tropical Medicine, 15-17 Tavistock Place, London WC1H 9SH, UK

[2] Behaviour and Health Research Unit, Institute of Public Health, University of Cambridge, Forvie Site, Robinson Way, Cambridge CB2 0SR, UK

[3] Nuffield Department of Primary Care Health Sciences, University of Oxford, Woodstock Rd, Oxford OX2 6GG, UK

*Corresponding author:

Richard.Smith@LSHTM.ac.uk

+44 (0)20 7927 2403

For peer review only

For peer review only - http://bmjopen.bmj.com/site/about/guidelines.xhtml

Abstract

Objectives

Taxing sugars-sweetened beverages (SSBs) is now advocated, and implemented, in many countries as a measure to reduce the purchase and consumption of sugar to tackle obesity. To date there has been little consideration of the potential impact that such a measure could have if extended to other sweet foods such as confectionery, cakes, and biscuits that contribute more sugar and energy to the diet than SSBs. The objective of this study is to compare changes in the demand for sweet snacks and sugar-sweetened beverages arising from price increases.

Setting

Secondary data on household itemised purchases of all foods and beverages form 2012-2013.

Participants

Representative sample of 32,249 households in Great Britain.

Primary and secondary outcome measures

Sensitivity to changes in food or beverage own price and to the price of other foods or beverages estimated for the full sample and by income groups.

Results

Chocolate and confectionery, cakes and biscuits have similar price sensitivity as sugar-sweetened beverages, across all income groups. Unlike the case of SSBs, price increases in these categories sometimes also prompt reductions in the purchase of other sweet snacks and SSBs, which magnify the overall impact. The effects of price rises are greatest in the low-income group.

Conclusions

Increasing the price of SSBs has become an accepted policy in the attempt to reduce sugar intake. This analysis suggests that policies increasing the price of chocolate confectionery, cakes, and biscuits, may lead to additional and greater health gains through direct reductions in the purchases of these foods and a positive multiplier effect that reduces demand for other products. Although some uncertainty remains, the associations found in this analysis are sufficiently robust to suggest that policies – and research – concerning the use of fiscal measures should consider a broader range of products than is currently the case.

Strengths and limitations of this study

- Detailed transaction level data on all food and beverage purchases collected electronically from a representative sample of >30,000 GB households over two years
- Demand analysis accounts for zero-purchases and endogeneity of total food expenditure
- Transaction level data allows for separating and analysing demand for ready-to-consume sweet snacks
- Data excludes purchases of foods and beverages bought and consumed outside homes
- Purchase data does not necessarily amount to consumption due to possible waste

For peer review only - http://bmjopen.bmj.com/site/about/guidelines.xhtml

Copyright

The Corresponding Author has the right to grant on behalf of all authors and does grant on behalf of all authors, a worldwide licence to the Publishers and its licensees in perpetuity, in all forms, formats and media (whether known now or created in the future), to i) publish, reproduce, distribute, display and store the Contribution, ii) translate the Contribution into other languages, create adaptations, reprints, include within collections and create summaries, extracts and/or, abstracts of the Contribution, iii) create any other derivative work(s) based on the Contribution, iv) to exploit all subsidiary rights in the Contribution, v) the inclusion of electronic links from the contribution to third party material where-ever it may be located; and, vi) licence any third party to do any or all of the above.

Competing interest statement

All authors have completed the ICMJE uniform disclosure form at http://www.icmje.org/coi_disclosure.pdf (available from the corresponding author) and declare: no support from any organisation for the submitted work; no financial relationships with any organisations that might have an interest in the submitted work in the previous three years, no other relationships or activities that could appear to have influenced the submitted work.

Author contributions

RS and TM conceived the study. DQ and LC conducted analyses, interpreted results and drafted the paper. RS, TM and SJ helped design the study, interpreted the results and drafted the paper. RS is guarantor of the study and affirms that the manuscript is an honest, accurate, and transparent account of the study being reported; that no important aspects of the study have been omitted; and that any discrepancies from the study as planned (and, if relevant, registered) have been explained.

Ethical approval

Ethical approval was not required as the data used in this study are secondary, anonymised purchase data. No patients were involved in the study.

Funding statement

This study was funded by the Department of Health in England Policy Research Programme (Policy Research Unit in Behaviour and Health (PR-UN-0409-10109)). LC is funded by an MRC Fellowship Grant (MR/P021999/1). Representatives of the Department of Health had no role in the data collection, analysis or interpretation, and no role in the study design or in writing the manuscript. The views expressed in this paper are those of the authors and not necessarily those of the Department of Health in England.

Data sharing statement

The data for this study were purchased from Kantar Worldpanel but its use is restricted to the persons named in the purchase contract which forbids the users to share the data with other potential (unnamed on the contract) users. Data access requests should be directed to Kantar Worldpanel.

For peer review only - http://bmjopen.bmj.com/site/about/guidelines.xhtml

Introduction

With the global prevalence of obesity, and associated health risks, rising,[1,2] health-related taxes have become an established policy option intended to reduce energy intake. Many of these have focussed on sugar-sweetened beverages (SSBs), due to their consistent association with energy intake, weight gain, risk of type 2 diabetes, as well as dental caries.[3] In the US at least 39 states and the cities of Chicago and Washington, D.C. have small value-added taxes on SSBs sold in grocery stores and/or vending machines,[4] Norway, Finland and France apply different levels of volumetric taxes on SSBs, Hungary has adopted a system of volumetric taxes from products exceeding specified levels of sugar, Mexico introduced a tax of 10% on non-alcoholic and non-dairy drinks, Chile has a tax of 8% on SSBs, and there are similar plans across a number of other countries such as India, the Philippines, Indonesia, Israel and South Africa.[5] The UK government has confirmed an industry levy to incentivise producers to reformulate their products or, if not, to increase the price of SSBs.[6]

Research to date suggests that increasing the price of SSBs generates a small, but significant, reduction in their purchase (broadly, a 10% price rise reduces purchase by 6-8%), with a more pronounced effect in poorer households, and that substitution towards other soft-drink categories only minimally offsets the energy reductions achieved through decreases in SSBs.[7-17] However, there has been little research on the impact such a price increase could have on other contributors to sugar and energy intake, including alcohol and sweet snack-foods (such as confectionery, cakes and biscuits). With the apparent success of fiscal measures to increase the price of SSBs, it would be useful to establish whether a similar, or possibly greater, effect on consumption of snack-foods could be obtained from a similar price change.

The research presented here is the first to provide a direct analysis of the relationship between price increases and demand for sweet snack foods, within the context of demand for soft- and alcoholic drink purchases, across different income groups.

Methods

We used a partial demand model, adapted from the Almost Ideal Demand System (AIDS), applied to household expenditure data from January 2012 to December 2013, provided by Kantar Worldpanel. Kantar Worldpanel includes information on household expenditures from a sample of British households (~36,000), representative of the population with respect to household size, number of children, social class, geographical region and age group, on food and drink purchases for home consumption made in a variety of outlets, including major retailers, supermarkets, butchers, greengrocers, and corner shops. The dataset consists of individual transactions, providing detailed information on the day of purchase, outlet, amount spent, volume purchased and also nutrient composition of each of the products, including sugar. Households record all purchases (barcodes and the receipts) for products brought back into the home with handheld scanners at home. In addition, Kantar Worldpanel annually collects socio-demographic information for each household, such as household size and composition, income group, social class, tenure and geographical location (postcode district), as well as age, gender, ethnicity and highest educational classification of the main shopper. As we are interested in analysing the demand across income groups we excluded households (4,075) for which this variable is missing (due to households' preference to not report this).

For peer review only - http://bmjopen.bmj.com/site/about/guidelines.xhtml

The full dataset used in the analysis thus consists of 32,249 households, of which 80% appear in both years (25,535), providing ~75 million food and beverage purchases disaggregated at the brand and package level, capturing both cross-sectional and longitudinal variation in household purchases.

For the analysis data were aggregated into 12 distinct groups for this study: (i) sugary drinks, containing more than 5g sugar/100 ml (assuming a dilution rate of 1:4 as used by the British Soft Drinks Association for concentrated SSBs); (ii) diet/low-sugar drinks, with less than 5g sugar/100 ml; (iii) other soft-drinks, including fruit juices, milk-based drinks (excluding pure milk) and water; (iv) alcohol, including beer, lager, cider, wines and spirits; (v) cookies, biscuits and cereal bars; (vi) chocolate and confectionary; (vii) cake-type snacks, including cake bars, pastries, muffins, flapjack and mince pies; (viii) savoury snacks, including crisps, popcorn, crackers and savoury assortments; (ix) fresh and frozen meat and fish; (x) dairy; (xi) fruit and vegetables; (xii) rest of food and drink. Snack foods – defined as foods which are at ambient temperature and able to be consumed on the go without utensils – were the most disaggregated as they were the focus for this study.

As many beverages and snack foods are storable and not purchased very frequently, data was aggregated at 4-week intervals for each household, providing a total of 699,854 household-month observations. As the data is aggregated to 4-weekly periods and into twelve groups we estimate geographical price indices from transaction prices of each individual product, based on postcode area the household resides at (see appendix 1for further details).

Even at this level of aggregation, a substantial amount of zero expenditure months remain, as most households do not buy beverages or foods from every category every month and some households never buy certain categories during the whole sample period. A two-step procedure was followed to take account of this censoring of the dependent variable in the estimation strategy. The AIDS approach was adapted for the panel data context to allow control for unobserved household heterogeneity via a fixed effects specification. The full specification, including the procedures for handling censoring, endogeneity of prices and total expenditure, and estimation of price elasticities is provided in the appendix 1. Analyses are carried out in the full sample and in subsamples by household annual income (low-income (< £20,000), mid-income (£20,000 - £49,000) and high-income (>£50,000+).

Patient involvement

No patients and the public were not involved in this research.

Role of funding source

This study was funded by the Department of Health in England Policy Research Programme (Policy Research Unit in Behaviour and Health (PR-UN-0409-10109)). LC is funded by an MRC Fellowship Grant (MR/P021999/1). Representatives of the Department of Health had no role in the data collection, analysis or interpretation, and no role in the study design or in writing the manuscript. The views expressed in this paper are those of the authors and not necessarily those of the Department of Health in England.

Results

Table 1 presents the socio-demographic profile of the sample. A comparison of Kantar Worldpanel with representative household data from the Living Cost and Food survey (LCF) has found the sociodemographic and regional profiles of the samples to match well, although our sample has a

For peer review only - http://bmjopen.bmj.com/site/about/guidelines.xhtml

For peer review only

slightly higher share of (i) low-income households, (ii) households that own a computer and/or a car, and (iii) households in the South and Southeast of England.[18]

TABLE 1 ABOUT HERE

Table 2 presents the average total purchases of sugar (expressed as grams per person per day) that are purchased and brought home (i.e. excluding purchases consumed outside homes), across each of the categories outlined above and split by income level. There is a clear income gradient: those on lower-incomes purchase more sugar per person per day. It is also clear that more sugar is consumed across all income groups from sweet snacks (16.9g), not beverages (including alcoholic and non-alcoholic) (13.9g). In comparison to SSBs subject to the proposed levy, sweet snacks combined, contribute more twice the amount of sugar.

TABLE 2 ABOUT HERE

Table 3 presents total expenditure, expenditure shares and average prices across all households and split into three income groups. The critical aspect for analysis here is the expenditure share, where there is a marked social gradient with respect to expenditure on beverages and slightly less for sweet snacks. The low-income group spend 18% of total drink expenditure on the sugary soft drinks, compared with 15% and 13% for medium- and high-income groups, respectively. Similarly, of the total food expenditure sweet snacks represent 7%, 7% and 6% among the low-, medium- and high-income groups, respectively.

TABLE 3 ABOUT HERE

The full results of the unconditional, uncompensated own- and cross price elasticities are presented in the appendix 2. In sum, the own-price elasticity for alcoholic drinks is higher than for all other categories; that is, alcoholic drinks are more sensitive to price change than any other category. Elasticities for all categories are inelastic (i.e. smaller than 1); this means that there is a less than proportionate decrease in purchase following a price rise for products, indicating that price increases reduce demand for all products, although with differing strength of effect. This pattern is seen across all income groups, with very similar absolute elasticity values. Comparing SSB and sweet snack price sensitivity, the elasticity for SSB is on average -0.8 (a 10% increase in price yields an 8% reduction in quantity purchased) whereas for chocolate and confectionary it is -0.74, biscuits -0.69, cakes -0.61 and for savoury snacks -0.76 with relatively little variance across income groups. Sweet snack foods, overall, thus appear to have a similar level of price sensitivity as SSBs.

Of interest also is the impact on purchases across other aspects of the diet when the price of SSBs or sweet snacks increases. Figures 1 to 4 present the (statically significant, $p<0.05$) impacts on purchases as a result of a 1% increase in price of each of the soft-drink and snack categories, to illustrate the variance in impact. This is presented for the total sample (figure 1) and then for each income group (figures 2-4).

FIGURES 1-4 ABOUT HERE

In aggregate across all income groups (figure 1) there are clear differences from increasing the price of SSBs compared with snacks. Increases in the price of sugary drinks are associated with increased purchases of other soft-drinks and cakes as a slight offset. Increasing the price of diet/low sugar drinks elicits greater reaction in other categories, with some increases in cakes, biscuits and chocolate (and a miniscule change increase in fruit/veg). For sweet snacks, there are considerably more complementary effects, with significant reductions in other categories. For chocolate & confectionary especially, there are significant decreases across all soft-drinks, biscuits and cakes. For

For peer review only - http://bmjopen.bmj.com/site/about/guidelines.xhtml

biscuits, there are significant reductions cakes, and for cakes there are smaller changes, with reductions in biscuits and but increases in alcohol. Thus, increasing the price of chocolate snacks especially elicits a range of significant reductions in purchases across most categories.

Although many of the associations at the aggregate level are replicated across income groups (figures 2-4), there is some clear variance by income group, as may be expected. An increase in the price of sugary-drinks is not associated with any other changes for the low- and high-income groups, but is associated with increases in other soft-drinks and cakes for the medium-income group. Increasing the price of diet/low sugar drinks seems to be associated with more substitute relationships, with significant increases in other snacks, especially for low- and medium-income groups. However, for increases in the price of sweet snacks the differences are more marked. Increasing the price of biscuits generates complementary reductions in the purchase of chocolate and confectionary for the low-income group, reductions in cakes for the middle-income group, but no such reductions for the high-income group. Changes in the price of cake-type snacks has limited impact on other categories for those in the low-income group, but for the middle-income group also reduces purchase of biscuits (and meat/fish and fruit/veg), but is also associated with a slight increase in purchase of alcohol. For the high-income group this effect is even more pronounced, with increases in purchase of alcohol and chocolate as substitutes. Increasing the price of chocolate and confectionary has a very similar effect across all income groups, with associated reductions in the purchase of most other food and drink categories.

Discussion

The price sensitivity of chocolate & confectionery highest among the sweet snacks and is almost identical to that for SSBs (although both are lower than alcohol). Further, price increases in SSBs are associated with an increase in purchase of other soft-drinks and cakes, whereas an increase in the price of chocolate is associated with a reduction in purchase of SSBs, as well as a range of other snacks. The differences across food categories, and income groups, indicates the complexity of estimating the impact of a single price increase. Nonetheless, it does suggest that – especially given that chocolate & confectionary alone contribute similar quantity of sugar per person per day than SSBs in our sample – policies to increase the price of sweet snacks could have a far greater impact than that seen thus far for SSBs. Not only are the own-price elasticities equivalent to those for SSBs, but they have greater level of association with reductions in other categories of foods and SSBs (i.e. complementary relationships), creating a cumulative positive multiplier effect. This appears to be most pronounced in the low- and middle-income groups, as would be expected, and for chocolate. The strength of these results suggests that further research is warranted to analyse the impact on diet composition and model the long-term impacts of such interventions on health outcomes.

There are, of course, limitations to the analysis presented here. The data, although large, representative and detailed, may be subject to under-recording; an issue present in all types of survey data. For instance, while Kantar Worldpanel matches the data on purchases that are brought into the home with scanned receipts to ensure accuracy. A comparison of Kantar Worldpanel data with the Living Cost and Food survey has shown the former to have lower levels of recorded alcohol expenditure for instance.[18] The data also includes foods and beverages purchased and bought home and thus excludes all purchases that are consumed outside homes. It is likely that the share of these purchases (and thus share of sugar purchased) is higher among mid- and high-income groups.

For peer review only - http://bmjopen.bmj.com/site/about/guidelines.xhtml

Regardless of the models used, estimating demand requires a number of assumptions (see appendix 1), which may have influenced the estimates. We prioritised an approach that allowed controlling for unobservable household heterogeneity, including in the preferences towards different types of drinks and snacks, while also adjusting for non-purchase and endogeneity issues. Overall, own-price elasticities are estimated with greater robustness as an *a priori* expectation of an inverse relationship with price exists and own prices have a noticeable impact on purchases. However, the estimation of substitution or complementarity effects across products are harder to capture, as these are generally much smaller and the direction cannot be assumed *a priori* for most products.[19] Perhaps more critically, although this analysis can highlight significant relationships between products purchased, it cannot explain why these relationships exist. This requires further primary research.

Conclusion

Increasing the price of SSBs has become an accepted policy to reduce sugar intake, especially in children. Analysis presented here suggests that extending fiscal policies to include sweet snacks could lead to larger public health benefits, both directly by reducing purchasing and therefore consumption of these foods, and indirectly by reducing demand for other snack foods and indeed SSBs. Although some uncertainty remains, the associations observed in this analysis are sufficiently robust to suggest that policies – and research – concerning the use of fiscal measures to reduce SSB consumption should consider extending to the more frequently consumed sugar-based snacks including cakes, biscuits and, especially, chocolate and confectionary.

For peer review only

For peer review only - http://bmjopen.bmj.com/site/about/guidelines.xhtml

References

1. OECD. Obesity Update. Paris: OECD; 2014.

2. WHO. Obesity and overweight fact sheet [Internet]. WHO. 2016 [cited 2016 Jun 28]. Available from: http://www.who.int/mediacentre/factsheets/fs311/en/

3. SACN. Carbohydrates and Health [Internet]. London: The Scientific Advisory Committee on Nutrition; 2015 [cited 2016 Aug 17]. Available from: https://www.gov.uk/government/publications/sacn-carbohydrates-and-health-report

4. Bridging the Gap, University of Illinois at Chicago. Liquid Candy: Existing Soft Drink Taxes [Internet]. 2011 [cited 2016 Jun 27]. Available from: http://cspinet.org/liquidcandy/existingtaxes.html

5. Public Health England. Sugar Reduction: The evidence for action Annexe 2: A mixed method review of behaviour changes resulting from experimental studies that examine the effect of fiscal measures targeted at high sugar food and non-alcoholic drink. London: Public Health England; 2015 Oct.

6. UK Department of Health. Childhood obesity: a plan for action [Internet]. London; 2016 Aug [cited 2016 Aug 22]. Available from: https://www.gov.uk/government/publications/childhood-obesity-a-plan-for-action

7. Cornelsen L, Green R, Turner R, Dangour AD, Shankar B, Mazzocchi M, et al. What Happens to Patterns of Food Consumption when Food Prices Change? Evidence from A Systematic Review and Meta-Analysis of Food Price Elasticities Globally. Health Econ. 2015 Dec 1;24(12):1548–59.

8. Zhen C, Finkelstein EA, Nonnemaker J, Karns S, Todd JE. Predicting the Effects of Sugar-Sweetened Beverage Taxes on Food and Beverage Demand in a Large Demand System. Am J Agric Econ. 2014 Jan 1;96(1):1–25.

9. Briggs AD, Mytton OT, Madden D, O'Shea D, Rayner M, Scarborough P. The potential impact on obesity of a 10% tax on sugar-sweetened beverages in Ireland, an effect assessment modelling study. BMC Public Health. 2013;13:860.

10. Briggs ADM, Mytton OT, Kehlbacher A, Tiffin R, Elhussein A, Rayner M, et al. Health impact assessment of the UK soft drinks industry levy: a comparative risk assessment modelling study. The Lancet Public Health. 2017 Jan;2(1):e15–22.

11. Colchero MA, Popkin BM, Rivera JA, Ng SW. Beverage purchases from stores in Mexico under the excise tax on sugar sweetened beverages: observational study. BMJ. 2016 Jan 6;352:h6704.

12. Colchero MA, Salgado JC, Unar-Munguía M, Hernández-Ávila M, Rivera-Dommarco JA. Price elasticity of the demand for sugar sweetened beverages and soft drinks in Mexico. Economics & Human Biology. 2015 Dec;19:129–37.

13. Dharmasena S, Capps O. Intended and unintended consequences of a proposed national tax on sugar-sweetened beverages to combat the U.S. obesity problem. Health Econ. 2012 Jun 1;21(6):669–94.

14. Paraje G. The Effect of Price and Socio-Economic Level on the Consumption of Sugar-Sweetened Beverages (SSB): The Case of Ecuador. PLOS ONE. 2016 Mar 30;11(3):e0152260.

For peer review only - http://bmjopen.bmj.com/site/about/guidelines.xhtml

15. Smith TA, Lin B-H, Lee J-Y. Taxing caloric sweetened beverages: potential effects on beverage consumption, calorie intake, and obesity. USDA-ERS Economic Research Report [Internet]. 2010 [cited 2016 Sep 14];(100). Available from: http://papers.ssrn.com/sol3/papers.cfm?abstract_id=2118636

16. Tiffin R, Kehlbacher A, Salois M. The Effects of A Soft Drink Tax in the UK. Health Econ. 2015 May 1;24(5):583–600.

17. Zhen C, Wohlgenant MK, Karns S, Kaufman P. Habit Formation and Demand for Sugar-Sweetened Beverages. Am J Agr Econ. 2011 Jan 1;93(1):175–93.

18. Leicester A. How might in-home scanner technology be used in budget surveys? [Internet]. Institute for Fiscal Studies; 2012 [cited 2017 Feb 20]. Report No.: W12/01. Available from: https://ideas.repec.org/p/ifs/ifsewp/12-01.html

19. Cornelsen L, Mazzocchi M, Green R, Dangour AD, Smith RD. Estimating the Relationship between Food Prices and Food Consumption—Methods Matter. Appl Econ Perspect Policy. 2016 Sep 1;38(3):546–61.

For peer review only

Tables

Table 1 Demographic characteristics of estimation sample

	All households	Low-income	Mid-income	High-income
Number of HH's	32,249	11,580	15,816	4,853
Number of observations	699,854	223,174	305,841	94,444
Household size	2.7 (1.3)	2.3 (1.3)	2.9 (1.3)	3.2 (1.2)
Age of main shopper	47.8 (15.3)	52.4 (17.0)	46.0 (14.3)	42.9 (10.8)
Number of children if have children	1.7 (0.8)	1.8 (0.9)	1.8 (0.9)	1.7 (0.8)
Share of households that have children	0.38 (0.5)	0.27 (0.5)	0.42 (0.5)	0.49 (0.5)
**Social grade**		%		
Class A&B (highly skilled)	20.2	5.7	20.9	52.5
Class C1	37.5	30.5	43	36.2
Class C2	18	15.6	22.4	9.2
Class D	13.9	22	11.7	1.7
Class E (unskilled)	10.4	26.2	1.9	0.3
**Highest qualification**		%		
Degree or higher	24.1	11.6	25.9	47.8
Higher education	13.5	11.6	15.2	12.1
A Level	11.6	10.0	13.2	10.6
GCSE	18.8	22.2	18.8	10.8
Other	7.6	11.6	6.0	3.1
None	7.6	15.2	4.1	0.9
Unknown	16.8	17.9	16.7	14.6
**Tenure**		%		
Owned outright	24.2	29.5	22.8	16.2
Mortgaged	40.0	17.1	47.6	69.7
Rented	29.7	46.4	23.6	9.8
Other	1.5	1.8	1.4	0.8
Unknown	4.7	5.2	4.7	3.6

Notes: Low income < £20,000 per year; mid-income £20,000 - £ 49,000; high-income >£50,000+

For peer review only

For peer review only - http://bmjopen.bmj.com/site/about/guidelines.xhtml

Table 2 Purchases of sugar (g) per person and day in 2013

	All households	Low-income	Mid-income	High-income
**Total sugar purchased per day per person (g)**	123.2	152.8	124.6	88.5
**Purchases of sugar by food group (g)**				
Sugary soft drinks	7.49	8.9	8.0	5.4
Diet soft drinks	0.46	0.5	0.5	0.4
Other soft drinks	3.90	3.8	4.2	4.0
Alcohol	2.01	2.2	2.3	1.6
Cookies (incl cereal fruit bars)	7.07	8.8	7.3	4.6
Chocolate & confectionary	7.68	9.9	7.7	5.2
Cake-type snacks	2.18	2.8	2.2	1.5
Savoury snacks	0.59	0.7	0.6	0.5
Fresh & frozen unprocessed meat, fish	0.53	0.6	0.6	0.4
Dairy & eggs	15.86	19.6	15.9	11.4
Fruit & Vegetables	17.64	20.7	17.9	14.2
Rest food & drink	57.77	74.2	57.4	39.4

Notes: Sugar content in purchase data are aggregated to total GB using weights provided by Kantar Worldpanel and divided by number of persons (total GB and by income groups) and days in a year. Total GB population figures are based on Kantar Worldpanel estimates of number of households in income brackets, taking into account the share of household members in each bracket (1, 2, 3 or 4 members and for households that had 5 or more members we used an average size of 5). Total GB population estimate (2013): ~59.5m, from which 27% are in households with annual income <£20,000 (low-income), 40% are in households with income £20,000 - £49,000 (mid-income) and 17% are in households with income >£50,000 (high-income). Households for which income is unknown or unanswered are excluded (14%).

For peer review only

Table 3 Mean total expenditure, expenditure shares and prices

	All households (n=699,854)		Low-income (n=223,174)		Mid-income (n=305,841)		High-income (n=94,444)	
	**Mean**	**St.Dev**	**Mean**	**St.Dev**	**Mean**	**St.Dev**	**Mean**	**St.Dev**
**Total monthly expenditure (£)**	183.5	110.6	155.0	96.3	194.1	112.2	211.9	121.3
**Expenditure share (%)**								
Sugary soft drinks	0.021	0.033	0.022	0.037	0.021	0.032	0.019	0.029
Diet soft drinks	0.019	0.030	0.017	0.030	0.020	0.030	0.022	0.032
Other soft drinks	0.016	0.026	0.013	0.025	0.016	0.025	0.020	0.028
Alcohol	0.079	0.125	0.071	0.127	0.083	0.126	0.087	0.124
Biscuits & cookies (incl. cereal fruit bars)	0.025	0.029	0.026	0.031	0.025	0.028	0.022	0.026
Chocolate & confectionary	0.028	0.041	0.031	0.045	0.027	0.038	0.024	0.037
Cake-type snacks	0.006	0.012	0.007	0.014	0.006	0.011	0.005	0.010
Savoury snacks	0.029	0.030	0.028	0.032	0.029	0.030	0.028	0.028
Fresh & frozen unprocessed meat, fish	0.129	0.092	0.122	0.095	0.130	0.090	0.137	0.092
Dairy & eggs	0.131	0.068	0.136	0.073	0.129	0.065	0.125	0.063
Fruit & vegetables	0.130	0.088	0.124	0.090	0.129	0.085	0.142	0.088
Rest food & drink	0.389	0.120	0.403	0.127	0.385	0.116	0.370	0.114
**Unit price[1]**	**Mean**	**St.Dev**	**Mean**	**St.Dev**	**Mean**	**St.Dev**	**Mean**	**St.Dev**
Sugary soft drinks	0.84	1.08	0.83	1.08	0.84	1.08	0.85	1.09
Diet soft drinks	0.70	1.09	0.69	1.09	0.70	1.09	0.71	1.10
Other soft drinks	0.86	1.08	0.86	1.08	0.86	1.08	0.87	1.08
Alcohol	4.67	1.13	4.65	1.13	4.67	1.13	4.75	1.13
Cookies (incl. cereal fruit bars)	3.77	1.07	3.76	1.06	3.77	1.07	3.80	1.07
Chocolate & confectionary	0.77	1.33	0.77	1.33	0.77	1.33	0.78	1.33
Cake-type snacks	1.00	1.06	0.99	1.06	1.00	1.06	1.00	1.06
Savoury snacks	3.33	1.05	3.33	1.05	3.33	1.05	3.35	1.05
Fresh & frozen unprocessed meat, fish	1.13	1.04	1.13	1.04	1.13	1.04	1.14	1.04
Dairy & eggs	5.65	1.06	5.62	1.06	5.65	1.06	5.71	1.07
Fruit & vegetables	0.98	1.07	0.98	1.07	0.98	1.07	0.99	1.07
Rest food & drink	1.66	1.10	1.65	1.09	1.66	1.09	1.69	1.10

Notes: [1] average unit prices (£) over geographical areas (n=110); Low income < £20,000 per year; mid-income £20,000 - £ 49,000; high-income >£50,000+

For peer review only - http://bmjopen.bmj.com/site/about/guidelines.xhtml

Figure 1. Change in demand (%) as a response to 1% price increase in soft drinks and sweet snacks (all households n=699,854)

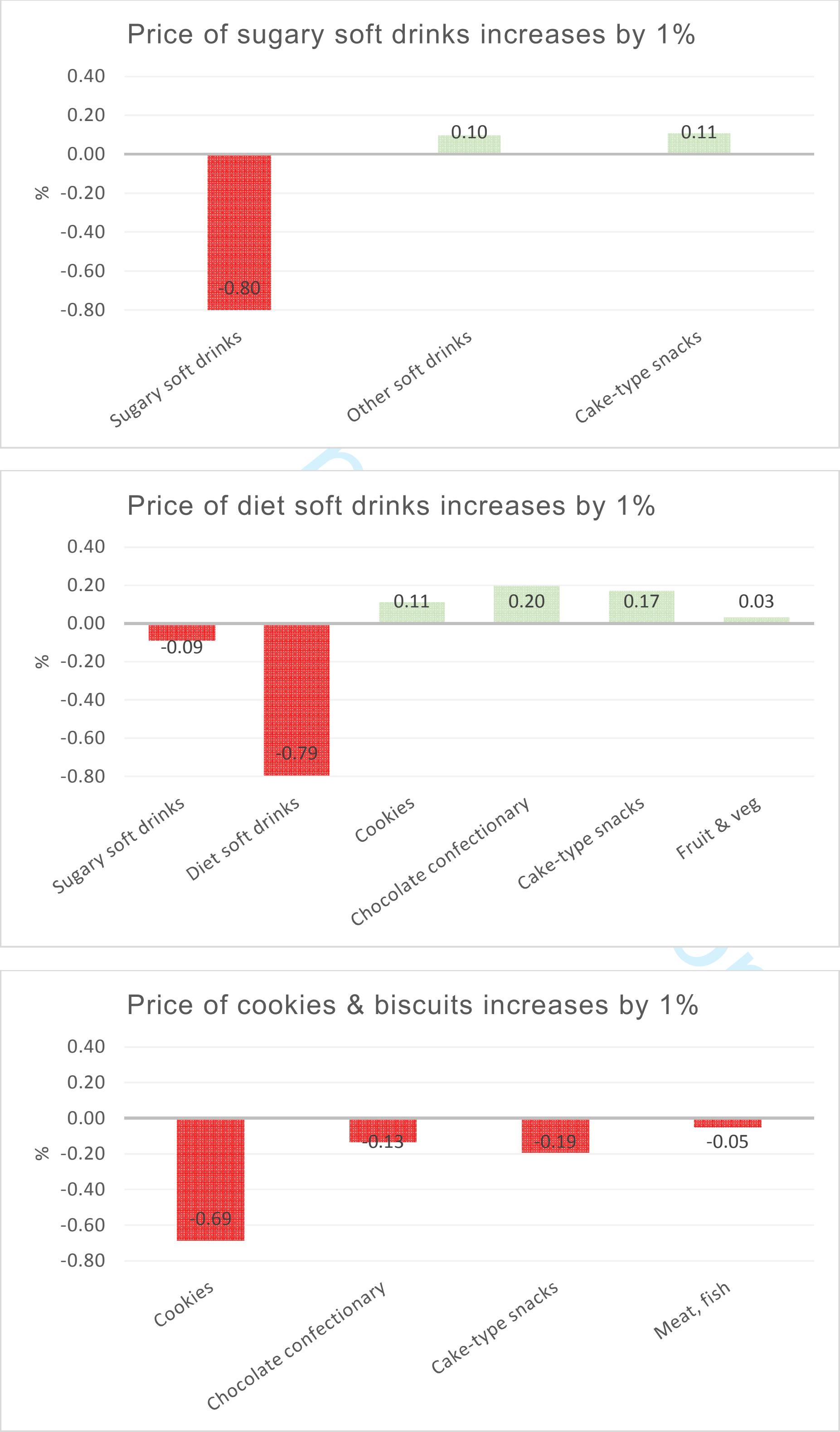

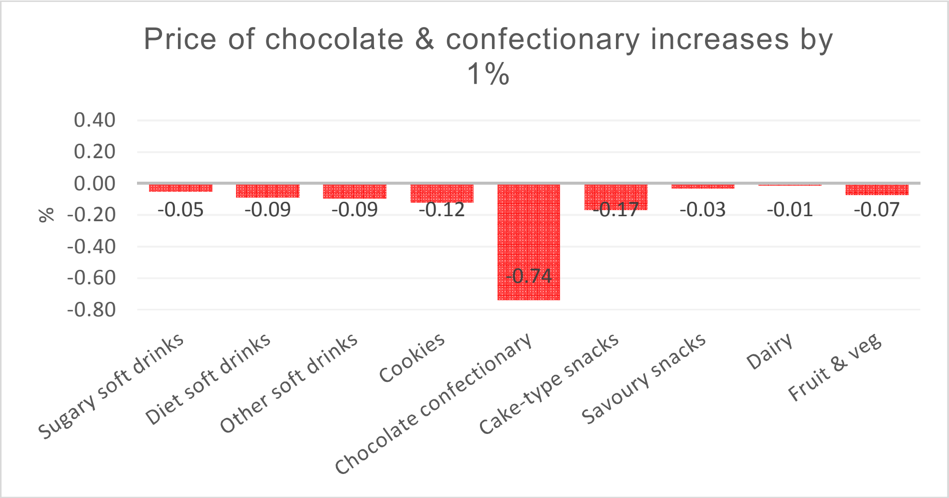
Price of chocolate & confectionary increases by 1%
0.40
0.20
0.00
-0.20
-0.40
-0.60
-0.80
%
-0.05
-0.09
-0.09
-0.12
-0.74
-0.17
-0.03
-0.01
-0.07
Sugary soft drinks
Diet soft drinks
Other soft drinks
Cookies
Chocolate confectionary
Cake-type snacks
Savoury snacks
Dairy
Fruit & veg

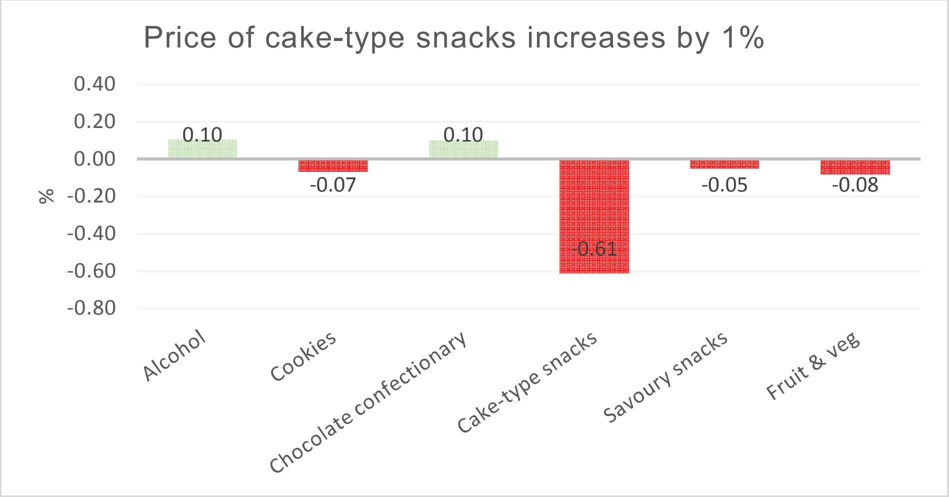
Price of cake-type snacks increases by 1%
0.40
0.20
0.00
-0.20
-0.40
-0.60
-0.80
%
0.10
-0.07
0.10
-0.61
-0.05
-0.08
Alcohol
Cookies
Chocolate confectionary
Cake-type snacks
Savoury snacks
Fruit & veg

Figure 2. Change in demand (%) as a response to 1% price increase in soft drinks and sweet snacks (low-income households n=233,174)

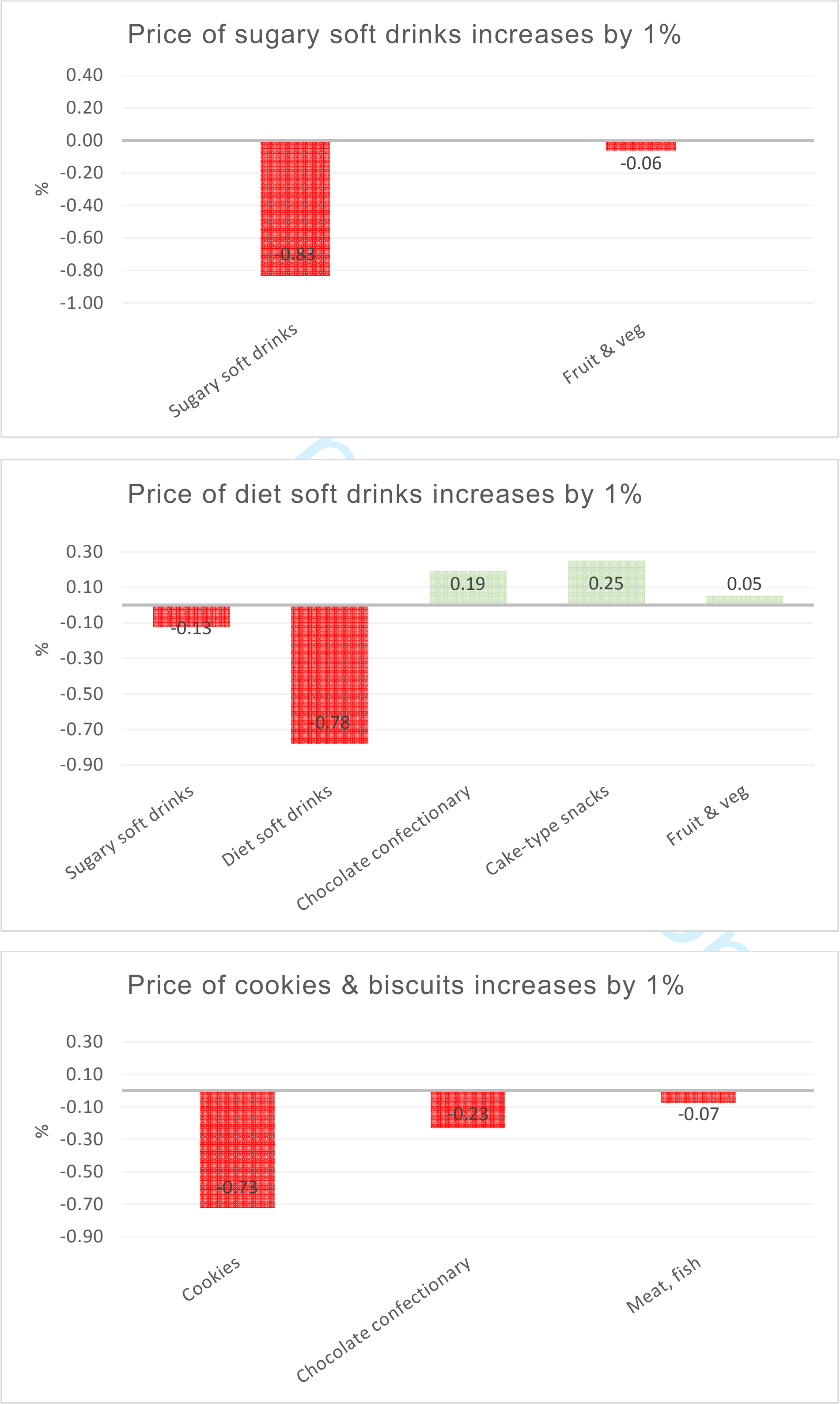

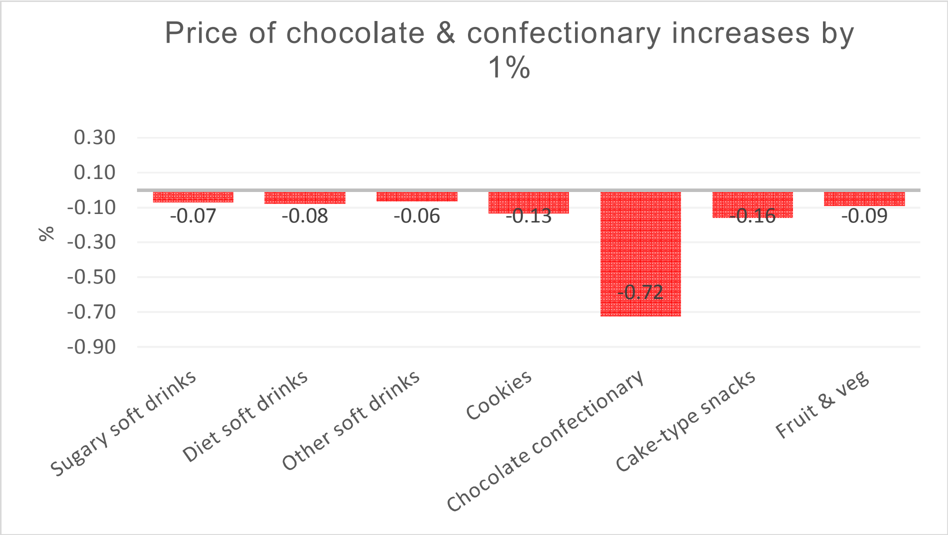

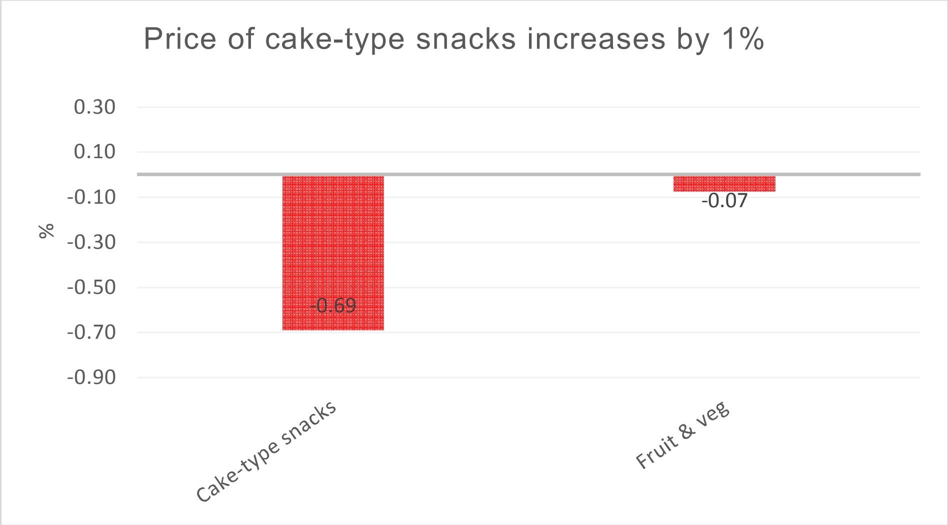

For peer review only

Figure 3. Change in demand (%) as a response to 1% price increase in soft drinks and sweet snacks (mid-income households n=305,841)

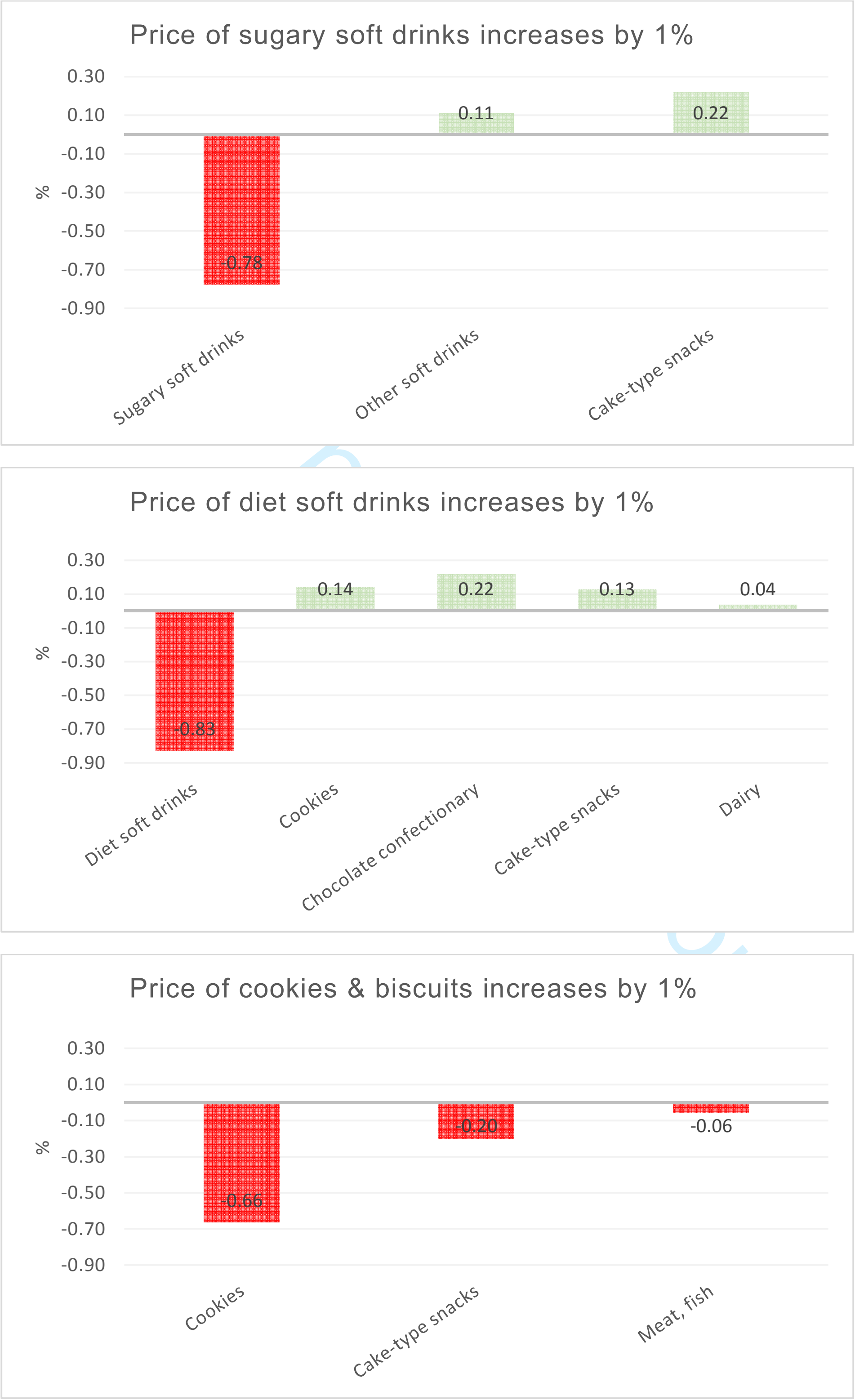

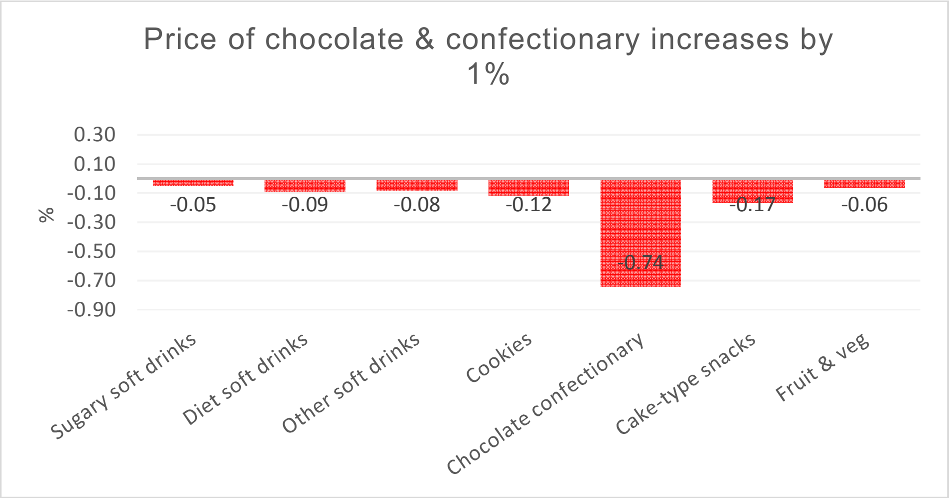
Price of chocolate & confectionary increases by 1%
0.30
0.10
-0.10
-0.30
-0.50
-0.70
-0.90
%
-0.05
-0.09
-0.08
-0.12
-0.74
-0.17
-0.06
Sugary soft drinks
Diet soft drinks
Other soft drinks
Cookies
Chocolate confectionary
Cake-type snacks
Fruit & veg

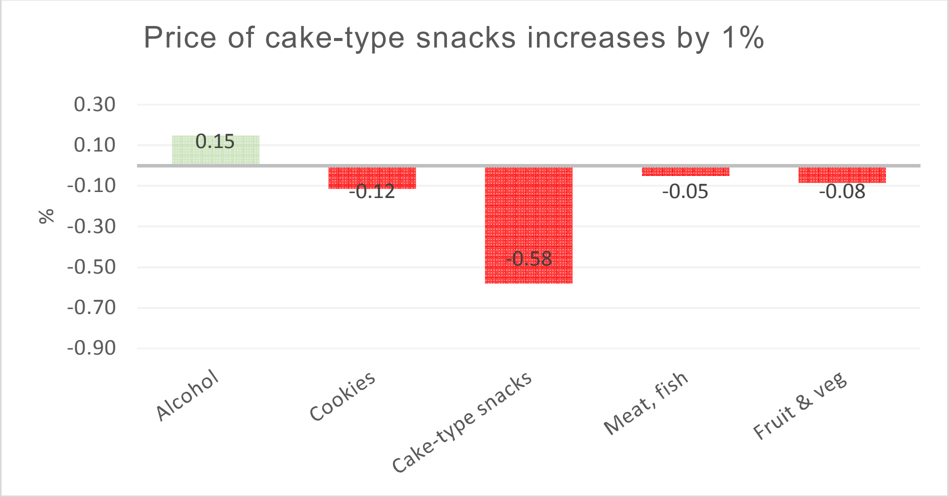
Price of cake-type snacks increases by 1%
0.30
0.10
-0.10
-0.30
-0.50
-0.70
-0.90
%
0.15
-0.12
-0.58
-0.05
-0.08
Alcohol
Cookies
Cake-type snacks
Meat, fish
Fruit & veg

Figure 4. Change in demand (%) as a response to 1% price increase in soft drinks and sweet snacks (high-income households n=94,444)

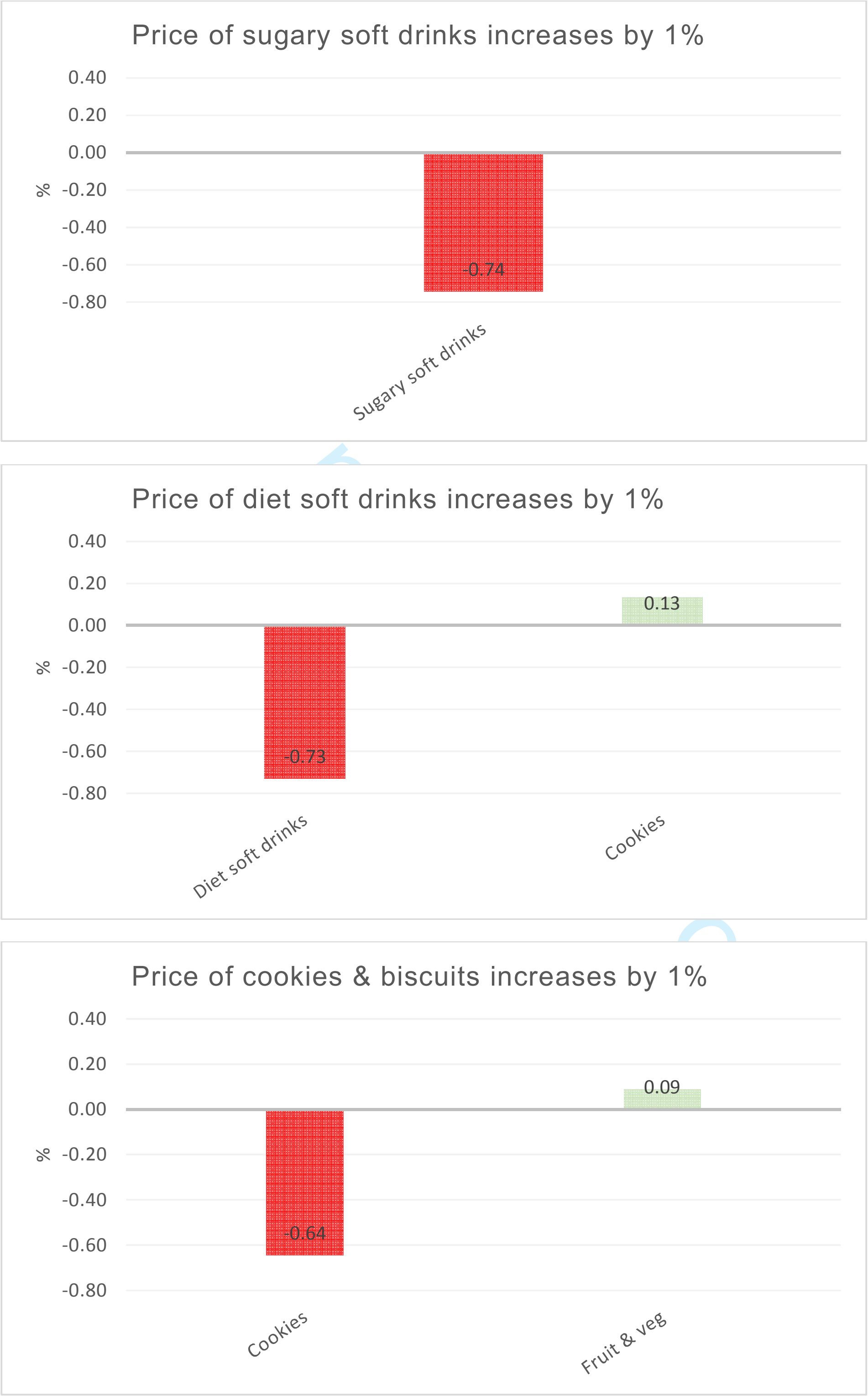

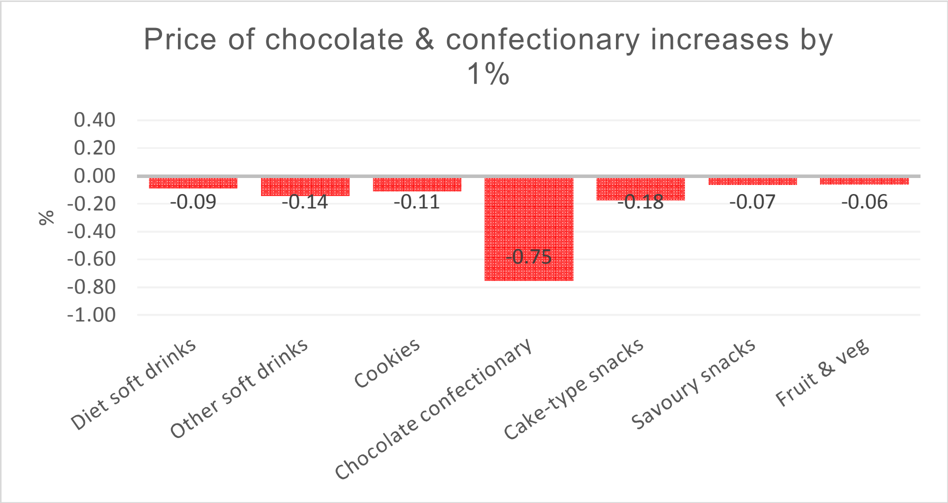

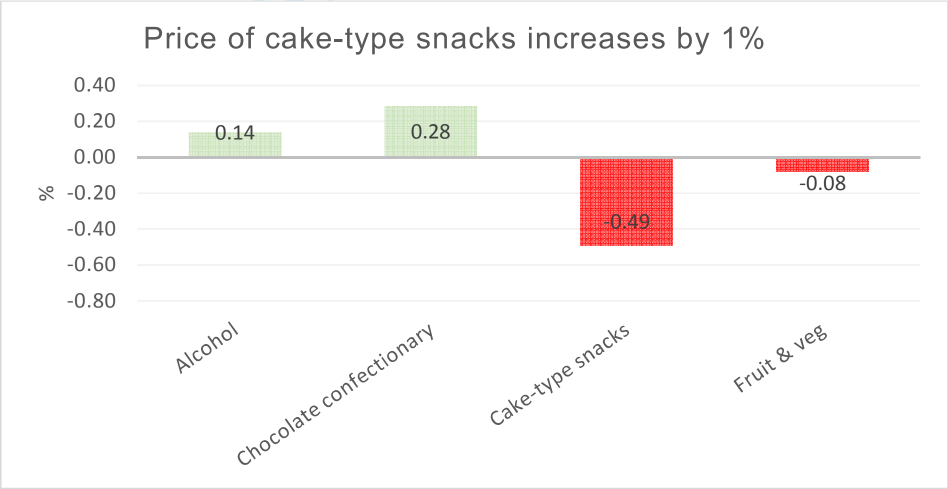

For peer review only - http://bmjopen.bmj.com/site/about/guidelines.xhtml

Appendix 1: Demand modelling strategy

The demand model applied was based on the linear version of Almost Ideal Demand System where expenditure shares are modelled as a function of prices and total expenditure (as an approximation for income) adjusted for all price levels:

$$w_{iht} = \alpha_i + \sum_{j=1}^{N} \gamma_{ij} lnp_{jht} + \beta_i \frac{lnx_{ht}}{P_{ht}} + \varepsilon_{iht} \qquad (1)$$

where:

w_{iht} is expenditure share of group *i* (i=1, 2, …, 12) for household *h* (h=1,2,…31,919) in 4-weekly periods *t* (t=1, 2, …, 26)

lnx_{ht} is the log of total household monthly expenditure on food and beverage per capita

lnp_{jht} is the log of price for category *j* for household *h* in period *t*

P_{ht} is a Laspeyres price index of geometrically weighted average prices defined as $lnP = \sum_i \overline{w}_i lnp_i$

ε_{iht} is a random disturbance

To deal with zero observations that can bias the estimates, we followed a two-step procedure developed in.[1] In the first step, the decision to purchase beverages in any group was modelled as a function of lagged quantity (L) of beverages purchased in that group, household size, age of the main shopper, socio-economic group (A&B, C1&C2 or D&E), whether or not the household owns their house, income group (for the whole sample only), presence of children and time indicators to take into account seasonal trends, using a probit model. From the probit model, we estimated the probability density function (ϕ_i) and cumulative density function (Φ_i) of the predictions of the fitted model. These two variables were applied in the second step of estimating the demand function (1):

$$w_{iht}^{*} = \Phi_{iht}(w_{iht}) + \varphi_i \phi_{iht} + \sum_{t=1}^{13} \rho_{it} T_{it} + v_{ih} + \varepsilon_{it} \qquad (2)$$

T_{it} are indicator variables to capture any seasonal or other time effects (13 four-week periods)

v_{ih} is a fixed household effect

For each of the twelve groups i=1, 2 ,…,12 we estimated (2) equation-by-equation using a fixed effect model with robust clustered standard errors to allow for any misspecification, particularly serial correlation of observations within the households. Clusters were defined at the geographical area used in estimating prices (n=110).

The specification used (2) imposed the restrictions, compatible with the AIDS model, of adding-up $[\sum_{i=1}^{N} \alpha_i = 1; \sum_{i=1}^{N} \beta_i = 0]$ and homogeneity $[\sum_{i=1}^{N} \gamma_{ij} = 0]$.

There are two important sources of potential endogeneity in the model. First, total expenditure enters the model as a proxy for incomes while it is also used to calculate the expenditure shares). Furthermore, total expenditure might be endogenous because of possible correlation with

For peer review only

unobserved characteristics affecting demand behaviour or because of shocks common to total expenditure and expenditure shares. Secondly, unit prices estimated from monthly aggregates of expenditure and volume are likely to be biased due to aggregation effects.[2] If prices or expenditures are correlated with the equation errors, estimators will be both biased and inconsistent.

To deal with quality effects in prices, we took the assumption that in a relatively small geographical area households face the same prices during the same time period. To estimate these geographical average unit values we calculated the monthly average prices for the (n=110) postcode areas which we observe in the data. Where the monthly price was missing (e.g. households did not purchase the products in this beverage group in a particular month), it was replaced by the first non-missing average of the previous and the following monthly prices.

To reduce possible endogeneity between expenditure shares (w_{iht}) and total expenditure (lnx_{ht}) that enters the demand equation in (1) we use the approach developed in [3] and regressed household per capita expenditure (lnx_{ht}) on household socio demographic characteristics (social class, income, income squared (whole sample only), household size and presence of children. The predicted values from the model were used as instruments for total expenditure (lnx_{ht}) in (1).

Uncompensated elasticities were estimated for beverages and individual beverage groups, at sample averages as follows:

$$e_{ij} = \Phi_i * \left(\frac{\gamma_{ij}}{w_i} - \frac{\beta_i w_j}{w_i}\right) - \Delta_{ij} \qquad (3)$$

Where Δ_{ij} is the Kronecker delta which equals 1 when *i=j* and 0 otherwise.

References

1. Shonkwiler JS, Yen ST. Two-Step Estimation of a Censored System of Equations. Am J Agr Econ. 1999 Nov 1;81(4):972–82.

2. Deaton A. Quality, quantity, and spatial variation of price. The American Economic Review. 1988;418–430.

3. Blundell R, Robin JM. Estimation in large and disaggregated demand systems: An estimator for conditionally linear systems. J Appl Econom [Internet]. 1999;14. Available from: http://dx.doi.org/3.0.CO;2-X

Appendix 2: Price elasticities

Table 1. Price elasticities of demand in full sample (n=699,854)

	**Price**											
All sample (n=699,854)	**Sugary soft drinks**	**Diet soft drinks**	**Other soft drinks**	**Alcohol**	**Biscuits & cookies**	**Choc & conf.**	**Cake-type snacks**	**Savoury snacks**	**Meat, fish**	**Dairy & eggs**	**Fruit & veg**	**Rest food & drink**
Sugary soft drinks	**-0.80**	**-0.09**	**-0.12**	**-0.22**	0.05	**-0.05**	-0.02	-0.02	**-0.12**	0.21	0.03	0.09
Diet soft drinks	-0.04	**-0.79**	**-0.12**	**-0.24**	-0.02	**-0.09**	-0.01	-0.02	0.09	**0.13**	0.08	-0.02
Other soft drinks	**0.10**	-0.03	**-0.82**	**-0.17**	-0.01	**-0.09**	0.02	0.03	**-0.15**	**0.15**	0.01	0.01
Alcohol	-0.02	-0.03	**-0.09**	**-0.90**	-0.04	0.02	**0.10**	-0.08	0.09	0.04	-0.06	**-0.31**
Biscuits & cookies	0.03	**0.11**	**0.05**	**0.05**	**-0.69**	**-0.12**	**-0.07**	0.07	**-0.17**	-0.04	0.01	**-0.38**
Chocolate & confectionary	0.06	**0.20**	0.03	**0.07**	**-0.13**	**-0.74**	**0.10**	0.02	**0.45**	**-0.27**	**-0.22**	**-0.85**
Cake-type snacks	**0.11**	**0.17**	**0.11**	-0.02	**-0.19**	**-0.17**	**-0.61**	-0.10	**-0.32**	0.09	-0.04	**-0.50**
Savoury snacks	0.02	0.02	-0.03	-0.03	-0.02	**-0.03**	**-0.05**	**-0.76**	-0.02	-0.01	0.06	**-0.24**
Fresh & frozen meat, fish	0.01	0.01	0.01	**0.03**	**-0.05**	0.00	-0.03	-0.01	**-0.76**	**-0.12**	**-0.09**	-0.03
Dairy & eggs	0.00	0.02	**0.03**	0.01	0.02	**-0.01**	-0.01	0.00	-0.03	**-0.88**	**-0.13**	**-0.09**
Fruit & vegetables	-0.02	**0.03**	-0.01	-0.02	0.03	**-0.07**	**-0.08**	**0.06**	**-0.10**	-0.01	**-0.60**	**-1.06**
Rest food & drink	-0.02	-0.05	0.02	0.00	0.01	0.03	-0.01	-0.01	-0.08	-0.02	-0.03	**-0.66**

Notes: Elasticities in bold are statistically significant at 5% level. Columns indicate the group of price change and rows indicate the group of demand change.

For peer review only

Table 2. Price elasticities of demand in low-income (annual household income £<20,000) sample (n=223,174)

	**Price**											
Low-income **(n=223,174)**	**Sugary soft drinks**	**Diet soft drinks**	**Other soft drinks**	**Alcohol**	**Biscuits & cookies**	**Choc & conf**	**Cake-type snacks**	**Savoury snacks**	**Meat, fish**	**Dairy & eggs**	**Fruit & veg**	**Rest food & drink**
Sugary soft drinks	**-0.83**	**-0.13**	-0.05	**-0.29**	0.04	**-0.07**	-0.06	0.00	0.04	**0.20**	0.01	0.08
Diet soft drinks	0.04	**-0.78**	**-0.17**	**-0.27**	-0.03	**-0.08**	-0.08	0.08	0.10	0.13	**0.20**	-0.20
Other soft drinks	0.10	0.11	**-0.88**	**-0.28**	-0.07	**-0.06**	0.01	0.05	**-0.27**	**0.20**	**0.26**	-0.17
Alcohol	0.02	-0.02	-0.03	**-0.91**	-0.02	0.01	-0.01	-0.06	0.17	**0.14**	-0.06	**-0.42**
Biscuits & cookies	0.00	0.07	0.05	0.00	**-0.73**	**-0.13**	0.00	0.03	-0.15	-0.02	0.04	**-0.31**
Chocolate & confectionary	0.08	**0.19**	0.04	0.07	**-0.23**	**-0.72**	0.06	-0.15	**0.49**	**-0.25**	**-0.31**	**-0.63**
Cake-type snacks	0.02	**0.25**	**0.15**	-0.09	-0.17	**-0.16**	**-0.69**	-0.03	**-0.39**	0.10	-0.06	-0.37
Savoury snacks	0.05	0.01	0.02	-0.04	-0.02	-0.04	-0.08	**-0.75**	0.01	-0.03	0.04	**-0.29**
Fresh & frozen meat, fish	0.02	0.00	0.03	**0.05**	**-0.07**	0.02	-0.01	0.07	**-0.80**	**-0.15**	**-0.11**	**-0.13**
Dairy & eggs	0.00	-0.02	0.03	0.02	0.02	-0.02	0.01	0.02	-0.07	**-0.86**	**-0.14**	-0.06
Fruit & vegetables	**-0.06**	**0.05**	-0.02	-0.02	0.02	**-0.09**	**-0.07**	0.08	-0.07	-0.03	**-0.57**	**-1.13**
Rest food & drink	-0.03	-0.05	-0.01	0.02	0.03	0.03	0.03	-0.04	-0.11	-0.06	-0.03	**-0.56**

Notes: Elasticities in bold are statistically significant at 5% level. Columns indicate the group of price change and rows indicate the group of demand change.

For peer review only

Table 3. Price elasticities of demand in mid-income (annual household income £20,000-£49,000) sample (n=223,174)

	Price											
Mid-income **(n=305,841)**	**Sugary soft drinks**	**Diet soft drinks**	**Other soft drinks**	**Alcohol**	**Biscuits & cookies**	**Choc & conf**	**Cake-type snacks**	**Savoury snacks**	**Meat, fish**	**Dairy & eggs**	**Fruit & veg**	**Rest food & drink**
Sugary soft drinks	**-0.78**	-0.06	**-0.15**	**-0.18**	0.06	**-0.05**	0.01	-0.01	-0.15	**0.18**	0.00	0.10
Diet soft drinks	-0.08	**-0.83**	-0.06	**-0.22**	0.00	**-0.09**	-0.02	-0.09	0.15	**0.15**	0.02	0.01
Other soft drinks	**0.11**	-0.04	**-0.78**	**-0.13**	0.04	**-0.08**	0.00	-0.02	-0.11	**0.15**	-0.06	0.00
Alcohol	-0.01	0.01	**-0.12**	**-0.91**	-0.04	0.02	**0.15**	-0.05	0.05	-0.03	**-0.09**	**-0.24**
Biscuits & cookies	0.06	**0.14**	0.05	**0.09**	**-0.66**	**-0.12**	**-0.12**	0.07	**-0.18**	-0.06	0.00	**-0.44**
Chocolate & confectionary	0.03	**0.22**	0.03	0.07	-0.04	**-0.74**	0.09	0.08	**0.42**	**-0.28**	**-0.16**	**-0.95**
Cake-type snacks	**0.22**	**0.13**	0.10	0.01	**-0.20**	**-0.17**	**-0.58**	-0.21	**-0.25**	0.03	-0.09	**-0.42**
Savoury snacks	0.01	0.02	**-0.06**	-0.03	-0.02	-0.02	-0.03	**-0.73**	-0.03	-0.02	0.04	**-0.25**
Fresh & frozen meat, fish	0.00	0.03	0.01	**0.03**	**-0.06**	0.00	**-0.05**	-0.04	**-0.75**	**-0.12**	**-0.08**	0.03
Dairy & eggs	-0.01	**0.04**	**0.03**	0.00	0.03	-0.01	-0.03	0.00	-0.01	**-0.88**	**-0.13**	-0.09
Fruit & vegetables	0.00	0.02	-0.03	-0.01	0.02	**-0.06**	**-0.08**	0.05	**-0.12**	0.02	**-0.58**	**-1.04**
Rest food & drink	-0.03	-0.08	0.04	-0.02	-0.01	0.03	-0.01	0.00	-0.07	0.01	-0.02	**-0.71**

Notes: Elasticities in bold are statistically significant at 5% level. Columns indicate the group of price change and rows indicate the group of demand change.

For peer review only

Table 4. Price elasticities of demand in high-income (annual household income £20,000-£49,000) sample (n=223,174)

	Price											
**High-income (n=94,444)**	**Sugary soft drinks**	**Diet soft drinks**	**Other soft drinks**	**Alcohol**	**Biscuits & cookies**	**Choc & conf**	**Cake-type snacks**	**Savoury snacks**	**Meat, fish**	**Dairy & eggs**	**Fruit & veg**	**Rest food & drink**
Sugary soft drinks	**-0.74**	-0.13	-0.12	**-0.21**	0.07	-0.03	0.00	-0.04	**-0.39**	**0.32**	0.11	0.17
Diet soft drinks	-0.06	**-0.73**	**-0.19**	**-0.24**	-0.09	**-0.09**	0.11	0.07	-0.18	0.08	0.08	0.26
Other soft drinks	0.07	-0.10	**-0.81**	-0.10	-0.01	**-0.14**	0.10	0.09	-0.18	0.02	-0.14	0.28
Alcohol	-0.08	-0.08	-0.07	**-0.82**	-0.09	0.03	**0.14**	**-0.21**	0.11	0.01	-0.02	-0.25
Biscuits & cookies	0.02	**0.13**	0.06	0.06	**-0.64**	**-0.11**	-0.08	0.15	-0.19	0.01	-0.10	-0.35
Chocolate & confectionary	0.09	0.14	0.02	0.13	-0.15	**-0.75**	**0.28**	**0.29**	**0.42**	**-0.28**	-0.15	**-1.06**
Cake-type snacks	-0.02	0.10	0.01	0.11	-0.20	**-0.18**	**-0.49**	0.16	-0.20	0.26	0.08	**-1.06**
Savoury snacks	-0.04	0.05	-0.07	-0.03	-0.04	**-0.07**	-0.05	**-0.86**	-0.05	0.08	**0.15**	-0.14
Fresh & frozen meat, fish	0.01	-0.02	-0.01	-0.01	-0.01	-0.01	0.00	-0.05	**-0.70**	-0.06	-0.05	-0.03
Dairy & eggs	0.03	0.02	0.04	-0.02	-0.01	-0.03	0.01	-0.10	0.02	**-0.93**	**-0.09**	**-0.23**
Fruit & vegetables	-0.03	0.03	0.04	-0.03	**0.09**	**-0.06**	**-0.08**	0.05	-0.08	-0.03	**-0.70**	**-0.93**
Rest food & drink	0.00	-0.02	0.01	-0.02	0.01	0.04	-0.08	0.05	-0.09	-0.02	-0.06	**-0.71**

Notes: Elasticities in bold are statistically significant at 5% level. Columns indicate the group of price change and rows indicate the group of demand change.

For peer review only

For peer review only - http://bmjopen.bmj.com/site/about/guidelines.xhtml

STROBE Checklist statement
Smith et al. "Reducing sugar consumption: are sweet snacks more sensitive to price increases than sugar-sweetened beverages?"

	Item No	Recommendation
**Title and abstract**	1	(*a*) Indicate the study's design with a commonly used term in the title or the abstract [abstract]
		(*b*) Provide in the abstract an informative and balanced summary of what was done and what was found [included]
**Introduction**		
Background/rationale	2	Explain the scientific background and rationale for the investigation being reported [reported on page 4]
Objectives	3	State specific objectives, including any prespecified hypotheses [reported on page 4]
**Methods**		
Study design	4	Present key elements of study design early in the paper [page 4]
Setting	5	Describe the setting, locations, and relevant dates, including periods of recruitment, exposure, follow-up, and data collection [page 4/5 where relevant; secondary data]
Participants	6	(*a*) Give the eligibility criteria, and the sources and methods of selection of participants
Variables	7	Clearly define all outcomes, exposures, predictors, potential confounders, and effect modifiers. Give diagnostic criteria, if applicable [demand model briefly explained in page 5, and explained in detail in technical appendix]
Data sources/ measurement	8*	For each variable of interest, give sources of data and details of methods of assessment (measurement). Describe comparability of assessment methods if there is more than one group [explained in text on page 5 or in/adjacent tables]
Bias	9	Describe any efforts to address potential sources of bias [described in brief in page 5, in technical appendix in detail]
Study size	10	Explain how the study size was arrived at [explained in page 4]
Quantitative variables	11	Explain how quantitative variables were handled in the analyses. If applicable, describe which groupings were chosen and why [explained in pages 4-5]
Statistical methods	12	(*a*) Describe all statistical methods, including those used to control for confounding [technical appendix]
		(*b*) Describe any methods used to examine subgroups and interactions [page 5]
		(*c*) Explain how missing data were addressed [page 4]
		(*d*) If applicable, describe analytical methods taking account of sampling strategy [n/a]
		(*e*) Describe any sensitivity analyses [n/a]
**Results**		
Participants	13*	(a) Report numbers of individuals at each stage of study—eg numbers potentially eligible, examined for eligibility, confirmed eligible, included in the study, completing follow-up, and analysed [n/a as secondary data]
		(b) Give reasons for non-participation at each stage [n/a]
		(c) Consider use of a flow diagram [n/a]
Descriptive data	14*	(a) Give characteristics of study participants (eg demographic, clinical, social) and information on exposures and potential confounders [table 1]

For peer review only - http://bmjopen.bmj.com/site/about/guidelines.xhtml

For peer review only

		(b) Indicate number of participants with missing data for each variable of interest [page 4 where relevant]
Outcome data	15*	Report numbers of outcome events or summary measures [tables 2-3]
Main results	16	(*a*) Give unadjusted estimates and, if applicable, confounder-adjusted estimates and their precision (eg, 95% confidence interval). Make clear which confounders were adjusted for and why they were included [figures 1-4, appendix 2, pages 6-7]
		(*b*) Report category boundaries when continuous variables were categorized [throughout tables]
		(*c*) If relevant, consider translating estimates of relative risk into absolute risk for a meaningful time period [n/a]
Other analyses	17	Report other analyses done—eg analyses of subgroups and interactions, and sensitivity analyses [pages 6-7]
**Discussion**		
Key results	18	Summarise key results with reference to study objectives [page 7]
Limitations	19	Discuss limitations of the study, taking into account sources of potential bias or imprecision. Discuss both direction and magnitude of any potential bias [page 7]
Interpretation	20	Give a cautious overall interpretation of results considering objectives, limitations, multiplicity of analyses, results from similar studies, and other relevant evidence [page 8]
Generalisability	21	Discuss the generalisability (external validity) of the study results [page 7]
**Other information**		
Funding	22	Give the source of funding and the role of the funders for the present study and, if applicable, for the original study on which the present article is based [page 3]

For peer review only

For peer review only - http://bmjopen.bmj.com/site/about/guidelines.xhtml

BMJ Open

Reducing sugar consumption: are sweet snacks more sensitive to price increases than sugar-sweetened beverages?

Journal:	*BMJ Open*
Manuscript ID	bmjopen-2017-019788.R1
Article Type:	Research
Date Submitted by the Author:	07-Dec-2017
Complete List of Authors:	Smith, Richard ; London School of Hygiene and Tropical Medicine, Faculty of Public Health and Policy<br>Cornelsen, Laura; London School of Hygiene and Tropical Medicine,<br>Quirmbach, Diana<br>Jebb, Susan; Univerof Oxford, Primary Care Health Sciences<br>Marteau, Theresa; University of Cambridge, Behaviour and Health Research Unit
**Primary Subject Heading**:	Health economics
Secondary Subject Heading:	Public health
Keywords:	price elasticity, sweet snacks, sugar-sweetened beverages, fiscal policy

SCHOLARONE™ Manuscripts

Reducing sugar consumption: are sweet snacks more sensitive to price increases than sugar-sweetened beverages?

Richard D Smith (PhD) [1,2]*

Laura Cornelsen (PhD) [1]

Diana Quirmbach (PhD) [1, 2]

Susan A. Jebb (PhD) [2,3]

Theresa Marteau (PhD) [2]

[1] Faculty of Public Health and Policy, London School of Hygiene and Tropical Medicine, 15-17 Tavistock Place, London WC1H 9SH, UK

[2] Behaviour and Health Research Unit, Institute of Public Health, University of Cambridge, Forvie Site, Robinson Way, Cambridge CB2 0SR, UK

[3] Nuffield Department of Primary Care Health Sciences, University of Oxford, Woodstock Rd, Oxford OX2 6GG, UK

*Corresponding author:

Richard.Smith@LSHTM.ac.uk

+44 (0)20 7927 2403

For peer review only

For peer review only - http://bmjopen.bmj.com/site/about/guidelines.xhtml

Abstract

Objectives

Taxing sugars-sweetened beverages (SSBs) is now advocated, and implemented, in many countries as a measure to reduce the purchase and consumption of sugar to tackle obesity. To date there has been little consideration of the potential impact that such a measure could have if extended to other sweet foods, such as confectionery, cakes and biscuits that contribute more sugar and energy to the diet than SSBs. The objective of this study is to compare changes in the demand for sweet snacks and sugar-sweetened beverages arising from potential price increases.

Setting

Secondary data on household itemised purchases of all foods and beverages from 2012-2013.

Participants

Representative sample of 32,249 households in Great Britain.

Primary and secondary outcome measures

Sensitivity of food and beverage purchase to changes in their own price and the price of other foods or beverages estimated for the full sample and by income groups.

Results

Chocolate and confectionery, cakes and biscuits have similar price sensitivity as SSBs, across all income groups. Unlike the case of SSBs, price increases in these categories are also likely to prompt reductions in the purchase of other sweet snacks and SSBs, which magnify the overall impact. The effects of price rises are greatest in the low-income group.

Conclusions

Policies that lead to increases in the price of chocolate and confectionery, cakes, and biscuits may lead to additional and greater health gains than similar increases in the price of SSBs through direct reductions in the purchases of these foods and possible positive multiplier effects that reduce demand for other products. Although some uncertainty remains, the associations found in this analysis are sufficiently robust to suggest that policies – and research – concerning the use of fiscal measures should consider a broader range of products than is currently the case.

Strengths and limitations of this study

- Detailed transaction level data on all food and beverage purchases collected electronically from a representative sample of >30,000 GB households over two years
- Transaction level data allows for separating and analysing demand for ready-to-consume sweet snacks
- Demand analysis accounts for zero-purchases and endogeneity of total food expenditure
- Data excludes purchases of foods and beverages bought and consumed outside homes
- Purchase data does not necessarily amount to consumption due to possible waste

For peer review only - http://bmjopen.bmj.com/site/about/guidelines.xhtml

For peer review only

Copyright
The Corresponding Author has the right to grant on behalf of all authors and does grant on behalf of all authors, a worldwide licence to the Publishers and its licensees in perpetuity, in all forms, formats and media (whether known now or created in the future), to i) publish, reproduce, distribute, display and store the Contribution, ii) translate the Contribution into other languages, create adaptations, reprints, include within collections and create summaries, extracts and/or, abstracts of the Contribution, iii) create any other derivative work(s) based on the Contribution, iv) to exploit all subsidiary rights in the Contribution, v) the inclusion of electronic links from the contribution to third party material where-ever it may be located; and, vi) licence any third party to do any or all of the above.

Competing interest statement
All authors have completed the ICMJE uniform disclosure form at http://www.icmje.org/coi_disclosure.pdf (available from the corresponding author) and declare: no support from any organisation for the submitted work; no financial relationships with any organisations that might have an interest in the submitted work in the previous three years, no other relationships or activities that could appear to have influenced the submitted work.

Author contributions
RS and TM conceived the study. DQ and LC conducted analyses, interpreted results and drafted the paper. RS, TM and SJ helped design the study, interpreted the results and drafted the paper. RS is guarantor of the study and affirms that the manuscript is an honest, accurate, and transparent account of the study being reported; that no important aspects of the study have been omitted; and that any discrepancies from the study as planned (and, if relevant, registered) have been explained.

Ethical approval
Ethical approval was not required as the data used in this study are secondary, anonymised purchase data. No patients were involved in the study.

Funding statement
This study was funded by the Department of Health in England Policy Research Programme (Policy Research Unit in Behaviour and Health (PR-UN-0409-10109)). LC is funded by an MRC Fellowship Grant (MR/P021999/1). Representatives of the Department of Health had no role in the data collection, analysis or interpretation, and no role in the study design or in writing the manuscript. The views expressed in this paper are those of the authors and not necessarily those of the Department of Health in England.

Data sharing statement
The data for this study were purchased from Kantar Worldpanel but its use is restricted to the persons named in the purchase contract which forbids the users to share the data with other potential (unnamed on the contract) users. Data access requests should be directed to Kantar Worldpanel.

For peer review only - http://bmjopen.bmj.com/site/about/guidelines.xhtml

Introduction

With the global prevalence of obesity and associated health risks continuing to increase,[1,2] health-related taxes have become an established policy option intended to reduce energy intake. Most of these have focussed on sugar-sweetened beverages (SSBs), due to their consistent association with energy intake, weight gain, risk of type-2 diabetes, as well as dental caries.[3] In the US, six local jurisdictions have a tax on sugary beverages implemented due to health concerns.[4] Mexico, Finland and France apply different levels of volumetric taxes on SSBs, Hungary has adopted a system of volumetric taxes from products exceeding specified levels of sugar, and Chile taxes drinks with high levels of sugar at a rate 8% higher in comparison to drinks containing less sugar.[4] More recently, Portugal and Catalonia (Spain) implemented a two-tiered tax on sugary drinks, the United Arab Emirates and Saudi Arabia introduced a 50% tax on carbonated drinks, and Brunei and Thailand introduced an excise duty on sugary drinks.[4] There are similar plans across a number of other countries such as Estonia, the Philippines, Indonesia, Israel and South Africa.[5] The UK government has confirmed an industry levy starting in April 2018 to incentivise producers to reformulate their products or, if not, to increase the price of SSBs.[6]

Research to date suggests that increasing the price of SSBs generates a small, but significant, reduction in their purchase (broadly, a 10% price rise reduces purchases by 6-8%), with a more pronounced effect in poorer households, and that substitution towards other soft-drink categories only minimally offsets the energy reductions achieved through decreases in SSBs.[7-17] However, there has been little research on the impact such a price increase could have on other contributors to sugar and energy intake, including alcohol and sweet snack-foods (such as confectionery, cakes and biscuits). With the apparent success of fiscal measures to increase the price of SSBs, it would be useful to establish whether a similar, or possibly greater, effect on consumption of snack-foods could be obtained from a similar price change.

The research presented here is the first to provide a direct analysis of the relationship between price increases and demand for sweet snack foods, within the context of demand for soft- and alcoholic drink purchases, across different income groups.

Methods

The impact, or sensitivity, of demand for sweet snack foods to changes in the price of non-alcoholic drinks is termed the price elasticity of demand. This shows the percent change in the demand for product X if its own price changes (own-price elasticity) or the price of other products (Y, Z) changes (cross-price elasticity). These elasticities are estimated from demand models. We apply a partial demand model, which models household expenditure shares on prices of different products and total expenditure as a proxy for income, adjusted for overall price level. The demand model we use is adapted from the common and widely applied Almost Ideal Demand System (AIDS).

The demand model and price elasticities are estimated from household expenditure data from January 2012 to December 2013, provided by Kantar Worldpanel. The data include information on household expenditures from a sample of British households (~36,000), representative of the population with respect to household size, number of children, social class, geographical region and age group, on food and drink purchases for home consumption made in a variety of outlets, including major retailers, supermarkets, butchers, greengrocers, and corner shops. The dataset consists of individual transactions, providing detailed information on the day of purchase, outlet, amount spent, volume purchased and also nutrient composition of each of the products, including

For peer review only - http://bmjopen.bmj.com/site/about/guidelines.xhtml

sugar. Households record all purchases (barcodes and the receipts) for products brought back into the home with handheld scanners at home. In addition, Kantar Worldpanel annually collects socio-demographic information for each household, such as household size and composition, income group, social class, tenure and geographical location (postcode district), as well as age, gender, ethnicity and highest educational classification of the main shopper. As we are interested in analysing the demand across income groups we excluded households (n=4,075) for which this variable is missing (due to households' preference to not report this).

The full dataset used in the analysis thus consists of 32,249 households, of which 80% appear in both years (25,535), providing ~75 million food and beverage purchases disaggregated at the brand and package level, capturing both cross-sectional and longitudinal variation in household purchases.

For analysis, data were aggregated from all foods and beverages into 13 distinct groups: (i) high-sugar soft drinks, containing more than 8g sugar/100 ml (assuming a dilution rate of 1:4 as used by the British Soft Drinks Association for concentrated SSBs); (ii) medium-sugar soft drinks, with between 5-8g sugar/100 ml; (iii) low-sugar soft drinks with less than 5g of sugar/100ml; (iv) other soft drinks, including fruit juices, milk-based drinks (excluding pure milk) and water[1]; (v) alcohol, including beer, lager, cider, wines and spirits; (vi) cookies, biscuits and cereal bars; (vii) chocolate and confectionary; (viii) cake-type snacks, including cake bars, pastries, muffins, flapjack and mince pies; (ix) savoury snacks, including crisps, popcorn, crackers and savoury assortments; (x) fresh and frozen meat and fish; (xi) dairy; (xii) fruit and vegetables; (xiii) rest of food and drink. Snack foods – defined as foods which are at ambient temperature and able to be consumed on the go without utensils – were the most disaggregated as these were the focus for this study.

As many beverages and snack foods are storable and not purchased very frequently, data were aggregated at 4-week intervals for each household, providing a total of n=623,459 household-month observations. As the data are aggregated to 4-weekly periods and into thirteen groups, we estimate geographical price indices from transaction prices of each individual product, based on the postcode area the households reside (see appendix 1 for further details).

Even at this level of aggregation, a substantial amount of zero-expenditure months remain, as most households do not buy beverages or foods from every category every month and some households never buy certain categories during the whole sample period. A two-step procedure was followed to take account of this censoring of the dependent variable in the estimation strategy. The AIDS approach was adapted for the panel data context to allow control for unobserved household heterogeneity via a fixed effects specification. The full specification, including the procedures for handling censoring, endogeneity of prices and total expenditure, and estimation of price elasticities is provided in appendix 1.

Due to potential differences in purchasing behaviour, the analyses are carried out in the full sample and in subsamples by household annual income (low-income (< £20,000), mid-income (£20,000 - £49,000) and high-income (>£50,000+).

Patient involvement

No patients or public were involved in this research.

---

[1] The categorisation of the non-alcoholic beverages follows the structure in the proposed levy for sugary drinks producers in the UK (effective April 2018)[6] separating drinks that would be levied at higher rate of £0.24 per L for drinks containing more than 8g of sugar per 100ml), at a lower rate of £0.18 per L (drinks containing between 5-8g of sugar/100ml) and not levied (drinks <5g sugar/100ml) and remaining soft drinks (juice with no added sugars, milk-based drinks and water).

For peer review only - http://bmjopen.bmj.com/site/about/guidelines.xhtml

Role of funding source

This study was funded by the Department of Health in England Policy Research Programme (Policy Research Unit in Behaviour and Health (PR-UN-0409-10109)). LC is funded by an MRC Fellowship Grant (MR/P021999/1). Representatives of the Department of Health had no role in the data collection, analysis or interpretation, and no role in the study design or in writing the manuscript. The views expressed in this paper are those of the authors and not necessarily those of the Department of Health in England.

Results

Table 1 presents the socio-demographic profile of the sample. A comparison of Kantar Worldpanel with representative household data from the Living Cost and Food survey (LCF) has found the sociodemographic and regional profiles of the samples to match well, although our sample has a slightly higher share of (i) low-income households, (ii) households that own a computer and/or a car, and (iii) households in the South and Southeast of England.[18]

TABLE 1 ABOUT HERE

Table 2 presents the average sugar content across the food and beverage groups as well as total purchases of sugar (expressed as grams per person per day) that are purchased and brought home (i.e. excluding purchases consumed outside homes), across each of the categories outlined above and split by income level. There is a clear income gradient: those on lower-incomes purchase more sugar per person per day. It is also clear that more sugar is consumed across all income groups from sweet snacks (16.9g), not beverages (including alcoholic and non-alcoholic) (13.9g). In comparison to SSBs, sweet snacks combined contribute more than twice the amount of sugar. It is also evident that sweet snacks have per 100g a considerably higher sugar content in comparison to 100ml of beverages.

TABLE 2 ABOUT HERE

Table 3 presents total expenditure, expenditure shares and average prices across all households and split into three income groups. The critical aspect for analysis here is the expenditure share, where there is a marked income gradient with respect to expenditure on beverages, and a slightly lower gradient for sweet snacks. The low-income group spend 14% of total drink expenditure on the sugary soft drinks, compared with 12% and 10% for medium- and high-income groups, respectively. Similarly, of the total food expenditure, sweet snacks represent 7%, 7% and 6% among the low-, medium- and high-income groups, respectively.

TABLE 3 ABOUT HERE

The full results of the unconditional, uncompensated own- and cross-price elasticities are presented in appendix 2. In sum, the own-price elasticity for alcoholic drinks is higher than for all other categories; that is, alcoholic drinks are more sensitive to price change than any other category. Elasticities for all categories are inelastic (i.e. smaller than 1); this means that there is a less than proportionate decrease in purchase following a price rise for products, indicating that price increases reduce demand for all products, although with differing strength of effect. This pattern is seen across all income groups, with relatively similar absolute elasticity values. Comparing SSB and sweet snack price sensitivity, the elasticity for SSB is on average -0.77 (a 10% increase in price yields a 7.7% reduction in quantity purchased) whereas for chocolate and confectionary it is -0.74, biscuits -0.69

For peer review only - http://bmjopen.bmj.com/site/about/guidelines.xhtml

and cakes -0.66. There is relatively little variance across income groups in the own-price elasticity for chocolate and confectionery whereas for biscuits and cookies and cake-type snacks, low-income households are relatively more price responsive (-0.74 and -0.71, respectively in comparison to -0.64 and -.53 in high-income group). Sweet snack foods, overall, thus appear to have only slightly lower level of price sensitivity in comparison to SSBs

Of interest also is the impact on purchases across other aspects of the diet when the price of SSBs or sweet snacks increases. Figures 1 to 4 present the impacts on purchases as a result of a 1% increase in price of each of the soft-drink and snack categories, to illustrate the variance in these effects (presenting only those effects where confidence intervals exclude zero). This is presented for the total sample (figure 1) and then for each income group (figures 2-4).

FIGURES 1-4 ABOUT HERE

Figure 1. Change in demand (%) as a response to 1% price increase in soft drinks and sweet snacks (all households n=623,459)

Figure 2. Change in demand (%) as a response to 1% price increase in soft drinks and sweet snacks (low-income households n=233,174)

Figure 3. Change in demand (%) as a response to 1% price increase in soft drinks and sweet snacks (mid-income households n=305,841)

Figure 4. Change in demand (%) as a response to 1% price increase in soft drinks and sweet snacks (high-income households n=94,444)

In aggregate across all income groups, (figure 1) clear differences arise from increasing the price of SSBs compared with snacks. Increases in the price of sugary drinks are associated with a decrease in purchases of medium-sugar soft drinks but increased purchases of other soft-drinks and chocolate and confectionary. Increasing the price of diet/low-sugar drinks elicits greater reaction in other soft drink purchases but also some increase in demand for cakes, biscuits and chocolate. Increasing the price of medium-sugar soft drinks, however, only slightly reduces demand for other soft drinks, low-sugar soft drinks and alcohol with no associations observed with demand for snacks.

For sweet snacks, there are considerably more complementary effects, with significant reductions in other categories. A price increase for chocolate and confectionary items is associated with small but significant decreases across all soft-drinks, biscuits and cakes. For biscuits, there are significant reductions in the demand for cakes as well as chocolate and confectionary. Finally for cakes there are smaller changes, with reductions in biscuits and but increases in chocolate and confectionary, and alcohol. Thus, increasing the price of chocolate snacks especially elicits a range of significant reductions in purchases across most categories.

Although many of the associations at the aggregate level are replicated across income groups (figures 2-4), there is some clear variance by income group. An increase in the price of sugary-drinks is associated with a reduction in medium-sugar drinks only within the low-income group (by 3% if price increases by 10%) while an increase in other soft-drinks is observed in medium- and high-income groups (1%). Furthermore, in the high-income group a higher SSB price leads to an increase in purchases of chocolate and confectionary (1-2%) but also a reduction in purchases of cake-type snacks (2% , albeit all with relatively large confidence intervals).

Increasing the price of diet/low sugar drinks seems to be associated with more substitute relationships, with significant increases in sweet snack demand (1-2% increase to a price increase of

For peer review only - http://bmjopen.bmj.com/site/about/guidelines.xhtml

10%), especially for low- and medium-income groups. However, for increases in the price of sweet snacks the differences are more marked. Increasing the price of biscuits generates complementary reductions in the purchase of chocolate and confectionary for the low-income group (by 3% if price increases by 10%), reductions in cake-type snacks for the middle-income group (3%), but no such reductions for the high-income group where a reduction in medium-sugar drinks is observed instead (8%). While a relatively large change, the absolute change would be small as the share of mid-sugar drinks in overall expenditure is very small.

Changes in the price of cake-type snacks has limited impact on other categories for those in the low-income group, but for the middle-income group it reduces purchase of biscuits (1%), but is also associated with a slight increase in purchase of alcohol (1%). For the high-income group this effect is even more pronounced, with increases in purchase of alcohol (1%) and chocolate as substitutes (3%). Increasing the price of chocolate and confectionary has a similar effect across all income groups, with associated reductions in the purchase of most other food and drink categories (1-2% if price increases by 10%).

Discussion

The price elasticity of chocolate & confectionery was highest among the sweet snacks and is almost identical to that for SSBs (although both are lower than alcohol). Further, price increases in SSBs are associated with an increase in purchase of other soft-drinks and chocolate and confectionary, whereas an increase in the price of chocolate is associated with a reduction in purchase of SSBs, as well as a range of other snacks. The differences across food categories, and income groups, indicates the complexity of estimating the impact of a single price increase. Nonetheless, it does suggest that policies to increase the price of sweet snacks could have a far greater impact than that seen thus far for SSBs, not least because chocolate and confectionery alone contribute a similar quantity of sugar per person per day as SSBs in our sample. More over this analysis suggests they have stronger associations with reductions in other categories of foods and SSBs (i.e. complementary relationships), creating a cumulative positive multiplier effect. This appears to be most pronounced in the low- and middle-income groups, as would be expected. The strength of these results suggests that further research is warranted to analyse the impact on diet composition and model the long-term impacts of such interventions on health outcomes.

The extent to which a levy on sugary snacks could yield a lower consumption of sugar is, of course, dependent on the structure of the levy, but considering the relatively high sugar content of these foods (per 100g) even a small levy based on sugar content is likely change prices, assuming it is passed through. Whether a multi-tiered levy based on sugar content, such as proposed for the sugary drinks, would encourage reformulation is another question since there are important differences in the ease of reformulation compared to SSBs and less is known about consumer acceptability of the reformulated snack food products.

Overall, our estimates of price-elasticity for foods and sugary beverages are consistent with the literature. Meta-analyses of price elasticity in broad food groups in high-income countries find these to range between -0.4 to -0.8 and that of sweets, confectionery and sweetened beverages at -0.6.[7,19] Our estimates range between -0.6 to -0.8 but we also use greater disaggregation of food and beverage groups. Another study reports the meta-estimate of price-elasticity of SSBs to be -1.3 that is higher than our estimate of -0.77, however the meta-estimate includes studies from Mexico and Brazil and price-elasticity is dependent on income levels and lower-income populations are likely to

For peer review only - http://bmjopen.bmj.com/site/about/guidelines.xhtml

have greater responsiveness to price changes (i.e. smaller elasticity value) as they spend a greater proportion of their incomes on food and beverages.[20] Equally, such differences may arise from variability in underlying preferences for foods and beverages in different countries. Elsewhere, a US study found, as here, a substitution effect towards juice and milk and a reduction in diet beverages if the price of SSBs increases. This study also estimated price-elasticity for SSBs at -0.8 and a somewhat less price responsive demand for sweets and sugars than our analysis (-0.3).[21]

There are, of course, limitations to the analysis presented here. The data, although large, representative and detailed, may be subject to under-recording; an issue present in all types of survey data. For instance, Kantar Worldpanel data appears to have lower levels of recorded alcohol expenditure than the Living Cost and Food survey.[18] The data also includes foods and beverages purchased and brought home and thus excludes all purchases that are consumed outside the home. Furthermore, the price responsiveness is based on price variations occurring in the market. This implies that any likely effect of the taxes inferred from these elasticities is subject to bias if the taxes, when implemented, have an impact on the demand beyond the direct price change.

Regardless of the models used, estimating demand requires a number of assumptions (see appendix 1), which may have influenced the estimates. We prioritised an approach that allowed controlling for unobservable household heterogeneity, including in the preferences towards different types of drinks and snacks, while also adjusting for non-purchase and endogeneity issues. Overall, own-price elasticities are estimated with greater robustness as an *a priori* expectation of an inverse relationship with price exists and own-price changes have a noticeable impact on purchases. However, the estimation of cross-price elasticities (substitution or complementarity effects) across products are harder to capture, as these are generally much smaller and the direction cannot be assumed *a priori*.[22] As most of cross-price elasticities are estimated close to zero, even small changes in methods can possibly affect the direction and thus interpretation of the effect. Perhaps more critically, although this analysis can highlight significant relationships between products purchased, it cannot explain why these relationships exist. This requires further primary research and research within population subgroups.

Conclusion

Increasing the price of SSBs has become an accepted policy to reduce sugar intake. Analysis presented here suggests that extending fiscal policies to include sweet snacks could lead to larger public health benefits, both directly by reducing purchasing and therefore consumption of these foods, and indirectly by reducing demand for other snack foods and indeed SSBs. Although some uncertainty remains, the associations observed in this analysis are sufficiently robust to suggest that policies – and research – concerning the use of fiscal measures to reduce SSB consumption should consider extending to the more frequently consumed sugar-based snacks including cakes, biscuits and, especially, chocolate and confectionary.

For peer review only - http://bmjopen.bmj.com/site/about/guidelines.xhtml

References

1. OECD. Obesity Update. Paris: OECD; 2014.

2. WHO. Obesity and overweight fact sheet [Internet]. WHO. 2016 [cited 2016 Jun 28]. Available from: http://www.who.int/mediacentre/factsheets/fs311/en/

3. SACN. Carbohydrates and Health [Internet]. London: The Scientific Advisory Committee on Nutrition; 2015 [cited 2016 Aug 17]. Available from: https://www.gov.uk/government/publications/sacn-carbohydrates-and-health-report

4. WCRF. NOURISHING Framework - Use of economic tools to address food affordability, purchase and incentives - Health-related food taxes [Internet] World Cancer Research Fund [cited 2017 Dec 01]. Available from http://www.wcrf.org/int/policy/nourishing-framework.

5. Public Health England. Sugar Reduction: The evidence for action Annexe 2: A mixed method review of behaviour changes resulting from experimental studies that examine the effect of fiscal measures targeted at high sugar food and non-alcoholic drink. London: Public Health England; 2015 Oct.

6. UK Department of Health. Childhood obesity: a plan for action [Internet]. London; 2016 Aug [cited 2016 Aug 22]. Available from: https://www.gov.uk/government/publications/childhood-obesity-a-plan-for-action

7. Cornelsen L, Green R, Turner R, Dangour AD, Shankar B, Mazzocchi M, et al. What Happens to Patterns of Food Consumption when Food Prices Change? Evidence from A Systematic Review and Meta-Analysis of Food Price Elasticities Globally. Health Econ. 2015 Dec 1;24(12):1548–59.

8. Zhen C, Finkelstein EA, Nonnemaker J, Karns S, Todd JE. Predicting the Effects of Sugar-Sweetened Beverage Taxes on Food and Beverage Demand in a Large Demand System. Am J Agric Econ. 2014 Jan 1;96(1):1–25.

9. Briggs AD, Mytton OT, Madden D, O'Shea D, Rayner M, Scarborough P. The potential impact on obesity of a 10% tax on sugar-sweetened beverages in Ireland, an effect assessment modelling study. BMC Public Health. 2013;13:860.

10. Briggs ADM, Mytton OT, Kehlbacher A, Tiffin R, Elhussein A, Rayner M, et al. Health impact assessment of the UK soft drinks industry levy: a comparative risk assessment modelling study. The Lancet Public Health. 2017 Jan;2(1):e15–22.

11. Colchero MA, Popkin BM, Rivera JA, Ng SW. Beverage purchases from stores in Mexico under the excise tax on sugar sweetened beverages: observational study. BMJ. 2016 Jan 6;352:h6704.

12. Colchero MA, Salgado JC, Unar-Munguía M, Hernández-Ávila M, Rivera-Dommarco JA. Price elasticity of the demand for sugar sweetened beverages and soft drinks in Mexico. Economics & Human Biology. 2015 Dec;19:129–37.

13. Dharmasena S, Capps O. Intended and unintended consequences of a proposed national tax on sugar-sweetened beverages to combat the U.S. obesity problem. Health Econ. 2012 Jun 1;21(6):669–94.

14. Paraje G. The Effect of Price and Socio-Economic Level on the Consumption of Sugar-Sweetened Beverages (SSB): The Case of Ecuador. PLOS ONE. 2016 Mar 30;11(3):e0152260.

For peer review only - http://bmjopen.bmj.com/site/about/guidelines.xhtml

15. Smith TA, Lin B-H, Lee J-Y. Taxing caloric sweetened beverages: potential effects on beverage consumption, calorie intake, and obesity. USDA-ERS Economic Research Report [Internet]. 2010 [cited 2016 Sep 14];(100). Available from: http://papers.ssrn.com/sol3/papers.cfm?abstract_id=2118636.

16. Tiffin R, Kehlbacher A, Salois M. The Effects of A Soft Drink Tax in the UK. Health Econ. 2015 May 1;24(5):583–600.

17. Zhen C, Wohlgenant MK, Karns S, Kaufman P. Habit Formation and Demand for Sugar-Sweetened Beverages. Am J Agr Econ. 2011 Jan 1;93(1):175–93.

18. Leicester A. How might in-home scanner technology be used in budget surveys? [Internet]. Institute for Fiscal Studies; 2012 [cited 2017 Feb 20]. Report No.: W12/01. Available from: https://ideas.repec.org/p/ifs/ifsewp/12-01.html.

19. Green R, Cornelsen L, Dangour AD, Turner R, Shankar B, Mazzocchi M, Smith RD. The Effect of Rising Food Prices on Food Consumption: systematic review with meta-regression. BMJ 2013; 346:f3703,p1-9.

20. Cabrera Escobar MA, Veerman LJ, Tollman SM, Bertram MY, Hofman KJ. Evidence that a tax on sugar sweetened beverages reduces the obesity rate: a meta-analysis. BMC Pub Health 2013, 13:1072, p1-10.

21. Andreyeva T, Long, MW, Brownell KD. The Impact of Food Pirces on Consumption: A Systematic Review of Research on the Price Elasticity of Demand for Food. Am J Public Health. 2010; 100:216-222.

22. Cornelsen L, Mazzocchi M, Green R, Dangour AD, Smith RD. Estimating the Relationship between Food Prices and Food Consumption—Methods Matter. Appl Econ Perspect Policy. 2016 Sep 1;38(3):546–61.

For peer review only

For peer review only - http://bmjopen.bmj.com/site/about/guidelines.xhtml

Tables

Table 1 Demographic characteristics of estimation sample

	All households	Low-income	Mid-income	High-income
Number of households	32,249	11,580	15,816	4,853
Number of observations	623,459	223,174	305,841	94,444
Household size (SD)	2.7 (1.3)	2.3 (1.3)	2.9 (1.3)	3.2 (1.2)
Age of main shopper (SD)	47.8 (15.3)	52.4 (17.0)	46.0 (14.3)	42.9 (10.8)
Number of children if have children (SD)	1.7 (0.8)	1.8 (0.9)	1.8 (0.9)	1.7 (0.8)
Share of households that have children	0.4 (0.5)	0.3 (0.5)	0.4 (0.5)	0.5 (0.5)
**Social grade**	%			
Class A&B (highly skilled)	20.2	5.7	20.9	52.5
Class C1	37.5	30.5	43.0	36.2
Class C2	18.0	15.6	22.4	9.2
Class D	13.9	22.0	11.7	1.7
Class E (unskilled)	10.4	26.2	1.9	0.3
**Highest qualification**	%			
Degree or higher	24.1	11.6	25.9	47.8
Higher education	13.5	11.6	15.2	12.1
A Level	11.6	10.0	13.2	10.6
Secondary education (GCSE)	18.8	22.2	18.8	10.8
Other	7.6	11.6	6.0	3.1
None	7.6	15.2	4.1	0.9
Unknown	16.8	17.9	16.7	14.6
**Tenure**	%			
Owned outright	24.2	29.5	22.8	16.2
Mortgaged	40.0	17.1	47.6	69.7
Rented	29.7	46.4	23.6	9.8
Other	1.5	1.8	1.4	0.8
Unknown	4.7	5.2	4.7	3.6

Notes: Low income < £20,000 per year; mid-income £20,000 - £ 49,000; high-income >£50,000+; GCSE – General Certificate of Secondary Education

For peer review only

Table 2 Purchases of sugar (g) per person and day in 2013

		All households	Low-income	Mid-income	High-income
Food group	Average sugar content (SD)[1]	Total sugar purchased per day per person (g)[2]			
High-sugar soft drinks	10.4 (1.7)	6.3	7.6	6.8	4.5
Medium-sugar soft drinks	6.5 (0.8)	0.6	0.7	0.6	0.4
Low-sugar soft drinks	1.0 (1.4)	1.1	1.2	1.2	0.9
Other soft drinks (incl. milk-based drinks)	7.5 (4.7)	3.9	3.8	4.2	4.0
Alcohol	1.4 (1.9)	2.0	2.2	2.3	1.6
Biscuits & cookies (incl. cereal fruit bars)	29.8 (10.5)	7.1	8.8	7.3	4.6
Chocolate & confectionary	48.7 (11.9)	7.7	9.9	7.7	5.2
Cake-type snacks	19.9 (11.4)	2.3	2.8	2.2	1.5
Savoury snacks	5.2 (8.1)	0.6	0.7	0.6	0.5
Fresh & frozen unprocessed meat, fish	1.0 (1.8)	0.5	0.6	0.6	0.4
Dairy & eggs	4.2 (5.0)	15.7	19.6	15.9	11.4
Fruit & Vegetables	6.2 (7.3)	17.6	20.7	17.9	14.2
Rest food & drink	13.2 (19.2)	57.8	74.2	57.4	39.4
Total		123.2	152.8	124.6	88.5

Notes: High-sugar soft drinks: >8g of sugar/100ml; medium-sugar soft drinks: 5-8g of sugar/100ml; low-sugar soft drinks: <5g of sugar/100ml; other soft drinks: water, fruit juice with no added sugars and milk-based drinks. [1] Average sugar content per 100g/100ml or item/unit (cake-type snacks and chocolate & confectionery). [2] Sugar content in purchase data is based on full data set of 2013 only (n=32,620), aggregated to total GB using weights provided by Kantar Worldpanel and divided by number of persons (total GB and by income groups) and days in a year. Total GB population figures are based on Kantar Worldpanel estimates of number of households in income brackets, taking into account the share of household members in each bracket (1, 2, 3 or 4 members and for households that had 5 or more members we used an average size of 5). Total GB population estimate (2013): ~59.5m, from which 27% are in households with annual income <£20,000 (low-income), 40% are in households with income £20,000 - £49,000 (mid-income) and 17% are in households with income >£50,000 (high-income). Households for which income is unknown or unanswered are excluded (14%).

For peer review only

Table 3 Mean total expenditure, expenditure shares and prices

	All households (n=623,459)		Low-income (n=223,174)		Mid-income (n=305,841)		High-income (n=94,444)	
	Mean	SD	Mean	SD	Mean	SD	Mean	SD
**Total 4-weekly expenditure (£)**	183.5	110.6	155.0	96.3	194.1	112.2	211.9	121.3
**Expenditure share**								
High-sugar soft drinks	0.015	0.028	0.015	0.032	0.015	0.027	0.013	0.015
Medium-sugar soft drinks	0.002	0.008	0.002	0.009	0.002	0.008	0.002	0.002
Low-sugar soft drinks	0.023	0.033	0.022	0.033	0.024	0.032	0.026	0.023
Other soft drinks	0.016	0.026	0.013	0.025	0.016	0.025	0.020	0.028
Alcohol	0.079	0.125	0.071	0.127	0.083	0.126	0.087	0.124
Biscuits & cookies (incl. cereal fruit bars)	0.025	0.029	0.026	0.031	0.025	0.028	0.022	0.026
Chocolate & confectionary	0.028	0.041	0.031	0.045	0.027	0.038	0.024	0.037
Cake-type snacks	0.006	0.012	0.007	0.014	0.006	0.011	0.005	0.010
Savoury snacks	0.029	0.030	0.028	0.032	0.029	0.030	0.028	0.028
Fresh & frozen unprocessed meat, fish	0.129	0.092	0.122	0.095	0.130	0.090	0.137	0.092
Dairy & eggs	0.131	0.068	0.136	0.073	0.129	0.065	0.125	0.063
Fruit & vegetables	0.130	0.088	0.124	0.090	0.129	0.085	0.142	0.088
Rest food & drink	0.389	0.120	0.403	0.127	0.385	0.116	0.370	0.114
**Price per volume unit (L, Kg)[1]**	**Mean**	**SD**	**Mean**	**SD**	**Mean**	**SD**	**Mean**	**SD**
High-sugar soft drinks	0.92	0.74	0.91	1.06	0.92	1.06	0.93	1.07
Medium-sugar soft drinks	0.95	0.49	0.95	1.17	0.95	1.18	0.97	1.18
Low-sugar soft drinks	0.69	0.50	0.69	1.10	0.69	1.10	0.71	1.11
Other soft drinks	0.86	1.08	0.86	1.08	0.86	1.08	0.87	1.08
Alcohol	4.67	1.13	4.65	1.13	4.67	1.13	4.75	1.13
Biscuits & cookies (incl. cereal fruit bars)	3.77	1.07	3.76	1.06	3.77	1.07	3.80	1.07
Chocolate & confectionary	0.77	1.33	0.77	1.33	0.77	1.33	0.78	1.33
Cake-type snacks	1.00	1.06	0.99	1.06	1.00	1.06	1.00	1.06
Savoury snacks	6.46	5.39	6.44	1.04	6.46	1.04	6.51	1.05
Fresh & frozen unprocessed meat, fish	5.65	4.62	5.62	1.06	5.65	1.06	5.71	1.07
Dairy & eggs	0.98	0.78	0.98	1.07	0.98	1.07	0.99	1.07
Fruit & vegetables	1.66	1.30	1.65	1.09	1.66	1.09	1.69	1.10
Rest food & drink	2.26	1.91	2.25	1.05	2.26	1.06	2.29	1.06

Notes: [1] average unit prices (£) over geographical areas (n=110); volume of cakes and chocolate & confectionery is measured by items; Low income < £20,000 per year; mid-income £20,000 - £ 49,000; high-income >£50,000+; High-sugar soft drinks: >8g of sugar/100ml; medium-sugar soft drinks: 5-8g of sugar/100ml; low-sugar soft drinks: <5g of sugar/100ml; other soft drinks: water, fruit juice with no added sugars and milk-based drinks.

For peer review only - http://bmjopen.bmj.com/site/about/guidelines.xhtml

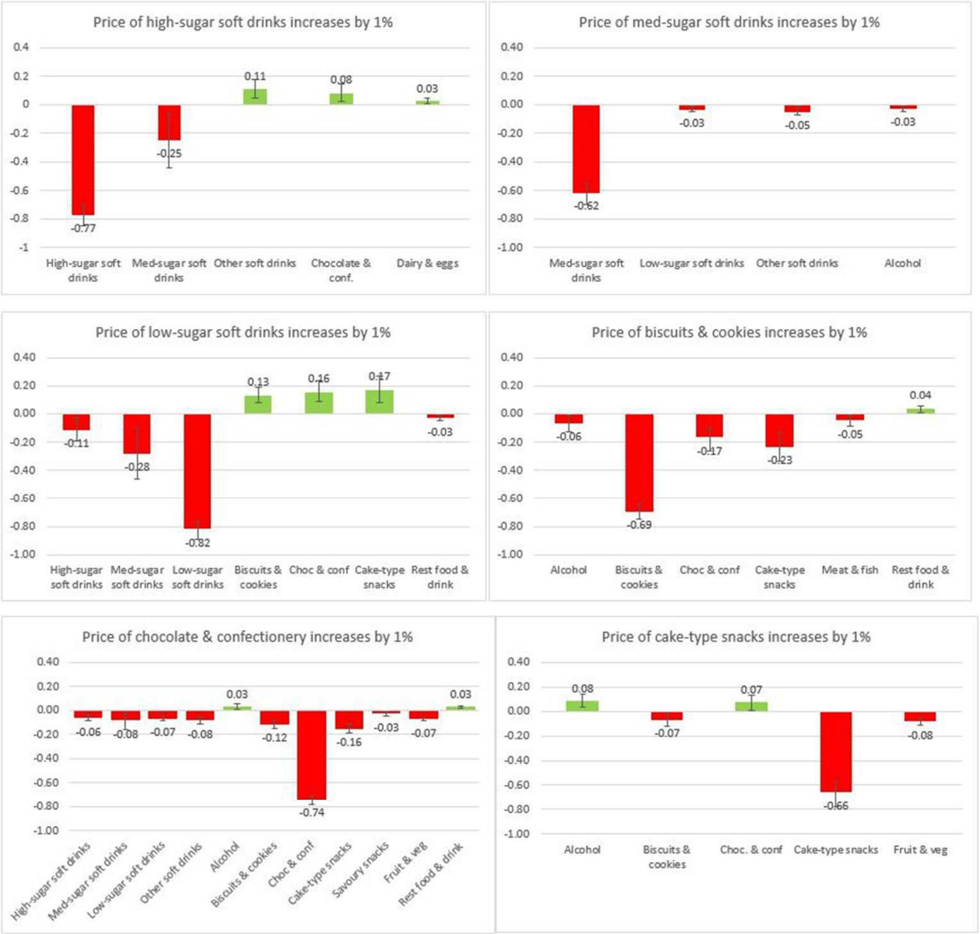

Notes: High-sugar soft drinks: >8g of sugar/100ml; medium-sugar soft drinks: 5-8g of sugar/100ml; low-sugar soft drinks: <5g of sugar/100ml; other soft drinks: water, fruit juice with no added sugars and milk-based drinks; 95% bias-corrected confidence intervals based on n=250 bootstrap replications.
Figures show elasticities for which the 95% CI excluded zero. For full set of elasticity, estimates see appendix 2.

Figure 1. Change in demand (%) as a response to 1% price increase in soft drinks and sweet snacks (all households n=623,459)

75x75mm (300 x 300 DPI)

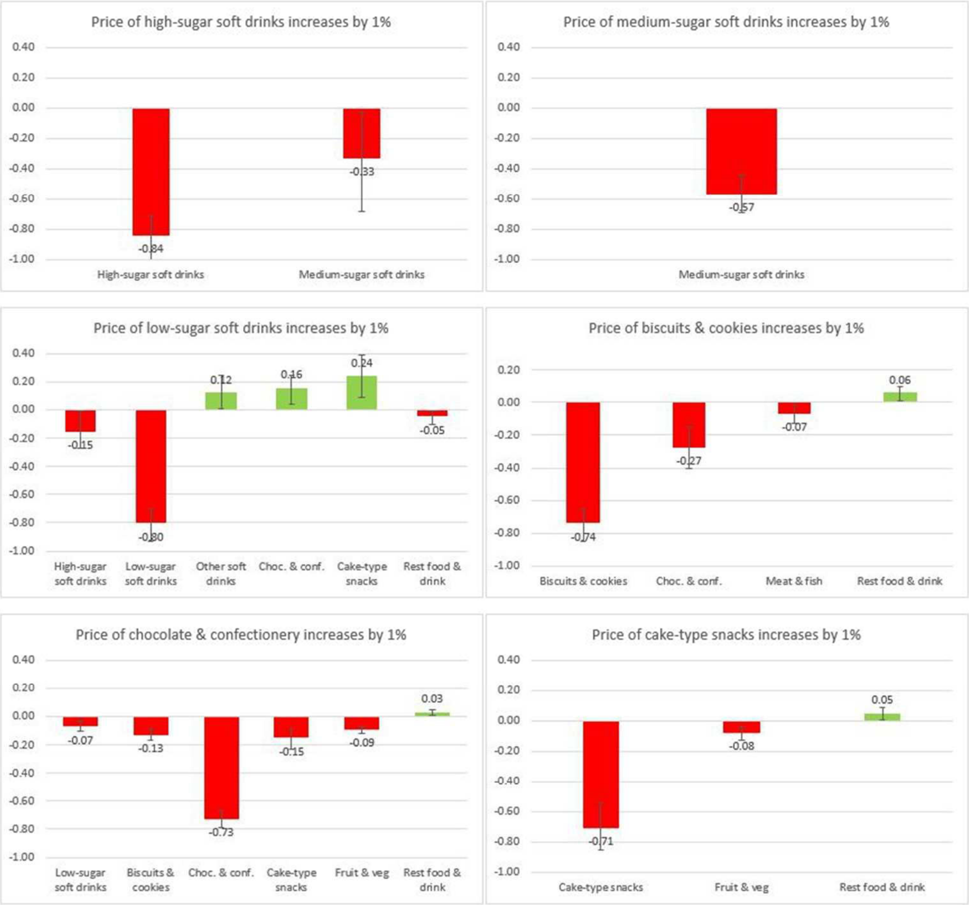

Notes: High-sugar soft drinks: >8g of sugar/100ml; medium-sugar soft drinks: 5-8g of sugar/100ml; low-sugar soft drinks: <5g of sugar/100ml; other soft drinks: water, fruit juice with no added sugars and milk-based drinks; 95% bias-corrected confidence intervals based on n=250 bootstrap replications.
Figures show elasticities for which the 95% CI excluded zero. For full set of elasticity, estimates see appendix 2.

Figure 2. Change in demand (%) as a response to 1% price increase in soft drinks and sweet snacks (low-income households n=233,174)

75x73mm (300 x 300 DPI)

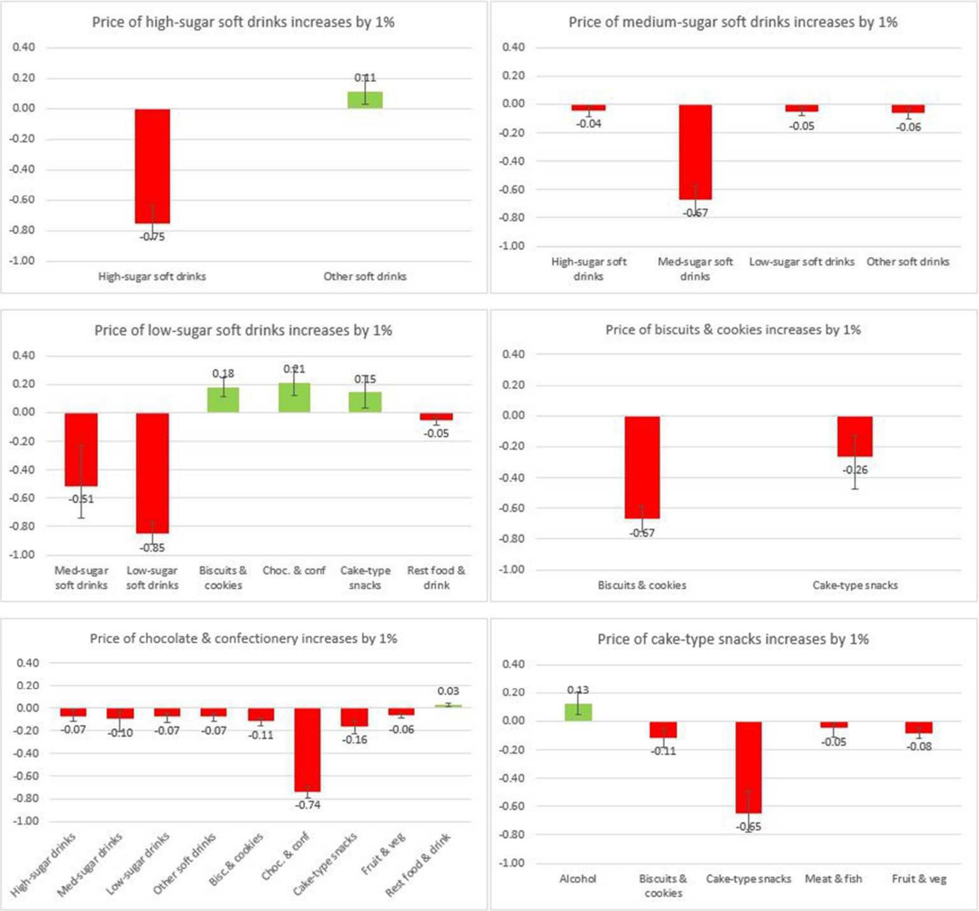

Notes: High-sugar soft drinks: >8g of sugar/100ml; medium-sugar soft drinks: 5-8g of sugar/100ml; low-sugar soft drinks: <5g of sugar/100ml; other soft drinks: water, fruit juice with no added sugars and milk-based drinks; 95% bias-corrected confidence intervals based on n=250 bootstrap replications.
Figures show elasticities for which the 95% CI excluded zero. For full set of elasticity, estimates see appendix 2.

Figure 3. Change in demand (%) as a response to 1% price increase in soft drinks and sweet snacks (mid-income households n=305,841)

74x73mm (300 x 300 DPI)

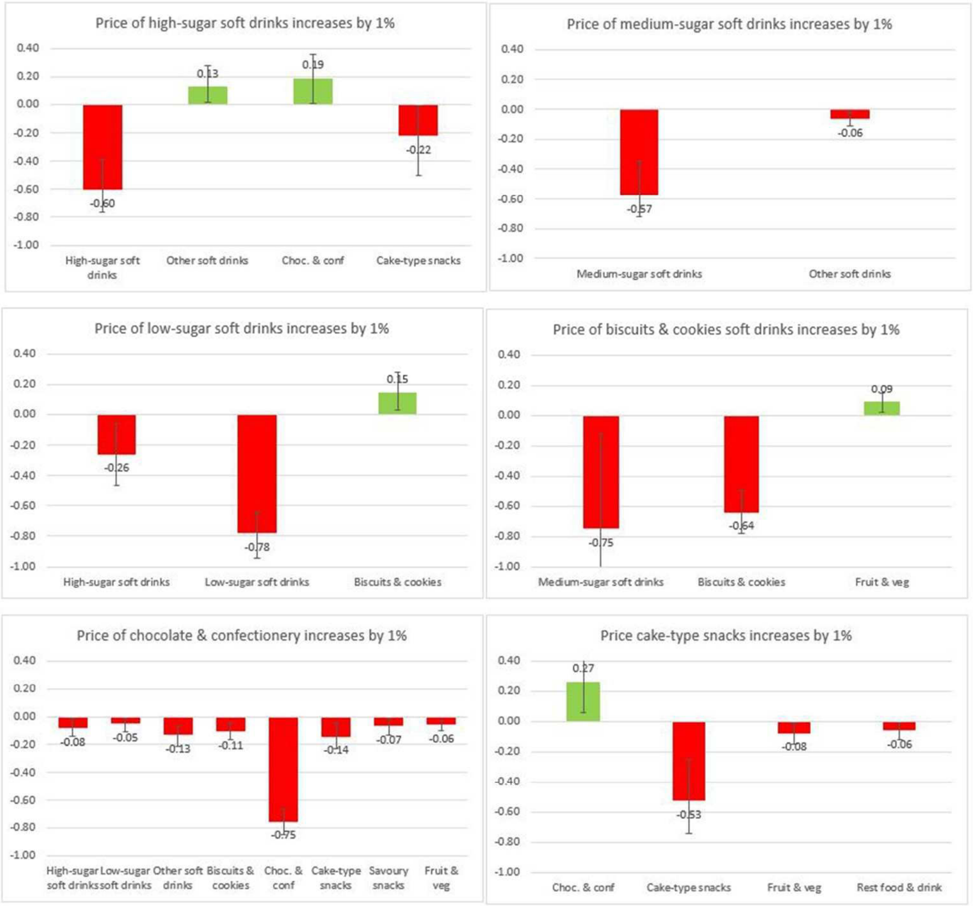

Notes: High-sugar soft drinks: >8g of sugar/100ml; medium-sugar soft drinks: 5-8g of sugar/100ml; low-sugar soft drinks: <5g of sugar/100ml; other soft drinks: water, fruit juice with no added sugars and milk-based drinks; 95% bias-corrected confidence intervals based on n=250 bootstrap replications.
Figures show elasticities for which the 95% CI excluded zero. For full set of elasticity, estimates see appendix 2.

Figure 4. Change in demand (%) as a response to 1% price increase in soft drinks and sweet snacks (high-income households n=94,444)

75x73mm (300 x 300 DPI)

Appendix 1: Demand modelling strategy

The demand model applied was based on the linear version of Almost Ideal Demand System where expenditure shares are modelled as a function of prices and total expenditure (as approximation for income) adjusted for all price levels:

$$w_{iht} = \alpha_i + \sum_{j=1}^{N} \gamma_{ij} lnp_{jht} + \beta_i \frac{lnx_{ht}}{P_{ht}} + \varepsilon_{iht} \quad (1)$$

Where:

w_{iht} is expenditure share of group *i* (i=1, 2, …, 13) for household *h* (h=1,2,…32,249) in 4-weekly periods *t* (t=1, 2, …, 26)

lnx_{ht} is the log of total household monthly expenditure on food and beverage per capita

lnp_{jht} is the log of price for category *j* for household *h* in period *t*

$P_{ht}$is a Laspeyres price index of geometrically weighted average prices defined as $lnP = \sum_i \overline{w}_i lnp_i$

α_i is a constant for group *i*

γ_{ij} and β_i are parameters to be estimated

ε_{iht} is a random disturbance

As not all households purchase items from each of the food and beverage groups in each period, the data includes zero-observations. These were more likely to occur in more disaggregated groups (e.g. 45% of observations among other soft drinks, 73% in cake-type snacks were zeroes). To deal with these zero observations that can bias the estimates, we followed a two-step procedure developed by Shonkweiler and Yen (1999).[1] In the first step, the decision to purchase beverages in any group was modelled as a function of lagged quantity of foods/beverages purchased in that group, household size, age of the main shopper, socio-economic group (A&B, C1&C2 or D&E), whether or not the household owns their house, income group (for the whole sample only), presence of children and time indicators to take into account seasonal trends, using a probit model. From the probit model, we estimated the probability density function (ϕ_i) and cumulative density function (Φ_i) of the predictions of the fitted model. These two variables were applied in the second step of estimating the demand function (1):

$$w_{iht}^{*} = \Phi_{iht}(w_{iht}) + \varphi_{iht}\phi_{iht} + \sum_{t=1}^{13} \rho_{it} T_{it} + v_{ih} + \varepsilon_{it} \quad (2)$$

Where:

T_{it} are indicator variables to capture any seasonal or other time effects (13 four-week periods)

v_{ih} is a fixed household effect

We estimated (2) equation-by-equation using a fixed effect model with robust clustered standard errors to allow for any misspecification, particularly serial correlation of observations within the households. Clusters were defined at the geographical area used in estimating prices (n=110).

The specification used (2) imposed the restrictions, compatible with the AIDS model, of adding-up $[\sum_{i=1}^{N} \alpha_i = 1; \sum_{i=1}^{N} \beta_i = 0]$ and homogeneity $[\sum_{i=1}^{N} \gamma_{ij} = 0]$.

For peer review only

There are two important sources of potential endogeneity in the model. First, total expenditure enters the model as a proxy for incomes while it is also used to calculate the expenditure shares. Furthermore, total expenditure might be endogenous because of possible correlation with unobserved characteristics affecting demand behaviour or because of shocks common to total expenditure and expenditure shares. Secondly, unit prices estimated from monthly aggregates of expenditure and volume are likely to be biased due to aggregation effects.[2] If prices or expenditures are correlated with the equation errors, estimators will be both biased and inconsistent.

To deal with quality effects in prices, we took the assumption that in a relatively small geographical area households face the same prices during the same time period. To estimate these geographical average unit values we calculated the monthly average prices for the (n=110) postcode areas which we observe in the data. Where the monthly price was missing (e.g. households did not purchase the products in this beverage group in a particular month), it was replaced by the first non-missing average of the previous and the following monthly prices.

To reduce possible endogeneity between expenditure shares (w_{iht}) and total expenditure (lnx_{ht}) that enters the demand equation in (1) we use the approach developed Blundell et al. (1999)[3] and regressed household per capita expenditure (lnx_{ht}) on household socio demographic characteristics (social class, income, income squared (whole sample only), household size and presence of children. The predicted values from the model were used as instruments for total expenditure (lnx_{ht}) in (1).

Uncompensated (Marshallian) elasticities were estimated for each beverage and food group, at sample averages (w and Φ) as follows:

$$e_{ij} = \Phi_i * \left(\frac{\gamma_{ij}}{w_i} - \frac{\beta_i w_j}{w_i}\right) - \Delta_{ij} \quad (3)$$

Where Δ_{ij} is the Kronecker delta which equals 1 when *i*=*j* and 0 otherwise.

Expenditure share equations in (2) are estimated with clustered (geographical area) robust standard errors and standard errors of the unconditional elasticities (3) are bootstrapped (250 replications) to account for possible bias arising from two-step procedure. Elasticities are reported with bias-corrected confidence intervals.

References

1. Shonkwiler JS, Yen ST. Two-Step Estimation of a Censored System of Equations. Am J Agr Econ. 1999 Nov 1;81(4):972–82.

2. Deaton A. Quality, quantity, and spatial variation of price. The Am Econ Rev. 1988;418–430.

3. Blundell R, Robin JM. Estimation in large and disaggregated demand systems: An estimator for conditionally linear systems. J Appl Econom. 1999;14(3):209-32.

Appendix 2: Price elasticities

Table 1. Price elasticities of demand in full sample (n=623,459)

	High-sugar soft drinks	Medium-sugar soft drinks	Low-sugar soft drinks	Other soft drinks	Alcohol	Biscuits & cookies	Chocolate & conf.	Cake-type snacks	Savoury snacks	Meat & fish	Dairy & eggs	Fruit & vegetables	Rest food & drink
High-sugar soft drinks	**-0.77 [-0.85;-0.70]**	-0.03 [-0.06;0]	**-0.11 [-0.19;-0.02]**	-0.05 [-0.11;0.02]	**-0.19 [-0.24;-0.14]**	0.02 [-0.07;0.10]	**-0.06 [-0.09;-0.04]**	-0.02 [-0.11;0.06]	**0.22 [0.11;0.35]**	**-0.29 [-0.41;-0.15]**	**0.26 [0.18;0.34]**	-0.08 [-0.17;0.01]	0 [-0.17;0.17]
Medium-sugar soft drinks	**-0.25 [-0.44;-0.06]**	**-0.62 [-0.70;-0.55]**	**-0.28 [-0.46;-0.10]**	**-0.33 [-0.53;-0.16]**	**-0.20 [-0.33;-0.06]**	-0.19 [-0.44;0.03]	**-0.08 [-0.16;-0.02]**	-0.06 [-0.28;0.14]	**-0.34 [-0.63;-0.07]**	**1.10 [0.76;1.38]**	**-0.22 [-0.43;-0.01]**	**0.37 [0.13;0.60]**	-0.08 [-0.51;0.41]
Low-sugar soft drinks	-0.01 [-0.06;0.05]	**-0.03 [-0.05;-0.01]**	**-0.82 [-0.89;-0.76]**	**-0.13 [-0.17;-0.09]**	**-0.25 [-0.29;-0.21]**	-0.02 [-0.08;0.05]	**-0.07 [-0.09;-0.05]**	0 [-0.05;0.06]	-0.01 [-0.10;0.07]	0.05 [-0.04;0.14]	**0.15 [0.09;0.21]**	**0.12 [0.07;0.19]**	-0.04 [-0.18;0.08]
Other soft drinks	**0.11 [0.05;0.18]**	**-0.05 [-0.07;-0.02]**	0 [-0.08;0.05]	**-0.83 [-0.88;-0.77]**	**-0.17 [-0.21;-0.12]**	-0.02 [-0.10;0.05]	**-0.08 [-0.11;-0.06]**	0.01 [-0.05;0.08]	0.06 [-0.04;0.17]	**-0.17 [-0.27;-0.07]**	**0.13 [0.06;0.21]**	0.03 [-0.04;0.10]	0.01 [-0.14;0.14]
Alcohol	-0.04 [-0.10;0.00]	**-0.03 [-0.05;-0.01]**	-0.02 [-0.08;0.03]	**-0.10 [-0.14;-0.05]**	**-0.90 [-0.94;-0.86]**	**-0.06 [-0.13;-0.01]**	**0.03 [0.01;0.05]**	**0.08 [0.03;0.14]**	**0.11 [0.04;0.19]**	0.08 [-0.01;0.17]	0.02 [-0.03;0.08]	**-0.07 [-0.14;-0.01]**	**-0.37 [-0.47;-0.25]**
Biscuits & cookies	0 [-0.06;0.04]	-0.01 [-0.02;0.02]	**0.13 [0.08;0.19]**	**0.05 [0.01;0.09]**	**0.06 [0.02;0.10]**	**-0.69 [-0.75;-0.64]**	**-0.12 [-0.15;-0.09]**	**-0.07 [-0.12;-0.02]**	**0.13 [0.03;0.20]**	**-0.18 [-0.28;-0.10]**	-0.04 [-0.11;0.01]	0 [-0.06;0.07]	**-0.40 [-0.51;-0.27]**
Chocolate & conf.	**0.08 [0.02;0.15]**	0.02 [-0.01;0.04]	**0.16 [0.09;0.24]**	0.01 [-0.04;0.07]	**0.07 [0.01;0.12]**	**-0.17 [-0.26;-0.10]**	**-0.74 [-0.78;-0.71]**	**0.07 [0.01;0.13]**	**0.27 [0.17;0.39]**	**0.44 [0.30;0.54]**	**-0.30 [-0.38;-0.22]**	**-0.26 [-0.35;-0.19]**	**-0.94 [-1.10;-0.77]**
Cake-type snacks	-0.02 [-0.11;0.06]	0 [-0.03;0.03]	**0.17 [0.08;0.27]**	0.09 [0;0.16]	-0.02 [-0.09;0.05]	**-0.23 [-0.33;-0.13]**	**-0.16 [-0.19;-0.11]**	**-0.66 [-0.78;-0.57]**	**0.31 [0.14;0.44]**	**-0.32 [-0.45;-0.14]**	0.06 [-0.04;0.17]	-0.08 [-0.17;0.02]	-0.08 [-0.17;0.02]
Savoury snacks	0 [-0.05;0.04]	0.01 [0;0.03]	0 [-0.05;0.05]	-0.04 [-0.07;0]	-0.03 [-0.06;0]	-0.02 [-0.07;0.04]	**-0.03 [-0.05;-0.01]**	-0.04 [-0.10;0.01]	**-0.75 [-0.82;-0.67]**	-0.03 [-0.12;0.05]	0 [-0.05;0.05]	0 [-0.05;0.05]	**-0.23 [-0.32;-0.11]**
Meat & fish	0 [-0.03;0.03]	0 [-0.01;0.01]	0.01 [-0.03;0.04]	0.01 [-0.01;0.04]	0.03 [0;0.05]	**-0.05 [-0.09;-0.01]**	0 [-0.01;0.02]	-0.02 [-0.05;0]	-0.04 [-0.10;0.01]	**-0.76 [-0.81;-0.70]**	**-0.12 [-0.15;-0.08]**	**-0.08 [-0.12;-0.04]**	-0.02 [-0.08;0.06]
Dairy & eggs	**0.03 [0.01;0.05]**	-0.01 [-0.02;0]	0.01 [-0.01;0.03]	**0.04 [0.02;0.06]**	0.01 [-0.01;0.02]	0.02 [0;0.05]	-0.01 [-0.03;0]	-0.01 [-0.04;0.01]	-0.02 [-0.06;0.02]	-0.03 [-0.06;0.02]	**-0.88 [-0.91;-0.85]**	**-0.12 [-0.14;-0.09]**	**-0.09 [-0.14;-0.03]**
Fruit & veg	-0.01 [-0.03;0.02]	0.00 [-0.01;0.01]	0 [-0.03;0.02]	-0.01 [-0.03;0]	-0.02 [-0.04;0.01]	0.03 [0;0.06]	**-0.07 [-0.09;-0.06]**	**-0.08 [-0.11;-0.06]**	**0.07 [0.03;0.12]**	**-0.10 [-0.14;-0.05]**	-0.01 [-0.04;0.02]	**-0.60 [-0.63;-0.57]**	-0.06 [-0.13;0.01]
Rest food & drink	-0.01 [-0.04;0.01]	0 [-0.01;0.01]	**-0.03 [-0.05;-0.01]**	**0.04 [0.02;0.06]**	0 [-0.01;0.02]	**0.04 [0.01;0.06]**	**0.03 [0.02;0.04]**	0 [-0.02;0.02]	**-0.13 [-0.16;-0.03]**	**-0.1 [-0.14;-0.06]**	-0.01 [-0.04;0.02]	-0.03 [-0.06;0]	**-0.66 [-0.71;-0.61]**

Notes: Elasticities in bold indicate those where 95% confidence intervals do not include zero. Columns indicate the group of price change and rows indicate the group of demand change. High-sugar soft drinks: >8g of sugar/100ml; medium-sugar soft drinks: 5-8g of sugar/100ml; low-sugar soft drinks: <5g of sugar/100ml; other soft drinks: water, fruit juice with no added sugars and milk-based drinks.

For peer review only - http://bmjopen.bmj.com/site/about/guidelines.xhtml

Table 2. Price elasticities of demand in low-income (annual household income £<20,000) sample (n=223,174)

	High-sugar soft drinks	Medium-sugar soft drinks	Low-sugar soft drinks	Other soft drinks	Alcohol	Biscuits & cookies	Chocolate & conf.	Cake-type snacks	Savoury snacks	Meat & fish	Dairy & eggs	Fruit & vegetables	Rest food & drink
High-sugar soft drinks	**-0.84 [-1.00;-0.71]**	-0.03 [-0.09;0.02]	**-0.15 [-0.27;-0.01]**	0.00 [-0.11;0.12]	**-0.25 [-0.36;-0.16]**	0.02 [-0.10;0.19]	-0.06 [-0.11;0]	-0.07 [-0.24;0.11]	0.17 [0;0.38]	-0.02 [-0.28;0.21]	**0.30 [0.15;0.44]**	-0.07 [-0.24;0.10]	-0.08 [-0.43;0.23]
Medium-sugar soft drinks	**-0.33 [-0.68;-0.02]**	**-0.57 [-0.69;-0.44]**	-0.05 [-0.38;0.26]	**-0.31 [-0.62;-0.07]**	-0.25 [-0.46;0]	-0.08 [-0.49;0.29]	-0.10 [-0.23;0.01]	-0.17 [-0.64;0.22]	-0.39 [-0.89;0.31]	**0.98 [0.39;1.46]**	**-0.39 [-0.71;-0.03]**	**0.43 [0.02;0.83]**	-0.06 [-0.87;0.73]
Low-sugar soft drinks	0 [-0.10;0.09]	-0.02 [-0.05;0.02]	**-0.80 [-0.93;-0.70]**	**-0.13 [-0.22;-0.05]**	**-0.28 [-0.35;-0.21]**	-0.01 [-0.13;0.10]	**-0.07 [-0.10;-0.03]**	-0.03 [-0.15;0.06]	0.05 [-0.18;0.17]	0.03 [-0.16;0.21]	**0.14 [0.03;0.25]**	**0.21 [0.10;0.33]**	-0.14 [-0.44;0.06]
Other soft drinks	0.08 [-0.04;0.23]	-0.01 [-0.05;0.05]	**0.12 [0.01;0.25]**	**-0.89 [-0.98;-0.80]**	**-0.27 [-0.37;-0.17]**	-0.08 [-0.22;0.08]	-0.06 [-0.11;-0.02]	0 [-0.12;0.13]	0.09 [-0.08;0.27]	**-0.27 [-0.51;-0.07]**	**0.19 [0.05;0.32]**	**0.26 [0.11;0.38]**	-0.17 [-0.41;0.06]
Alcohol	-0.01 [-0.12;0.09]	-0.04 [-0.08;0]	0.01 [-0.06;0.11]	-0.05 [-0.14;0.02]	**-0.92 [-0.98;-0.85]**	-0.04 [-0.15;0.06]	0.02 [-0.01;0.06]	-0.04 [-0.13;0.06]	**0.17 [0.05;0.33]**	0.15 [-0.02;0.30]	0.12 [-0.01;0.21]	-0.08 [-0.17;0.06]	**-0.50 [-0.69;-0.28]**
Biscuits & cookies	0.01 [-0.09;0.10]	-0.03 [-0.06;0]	0.07 [0;0.16]	0.04 [-0.03;0.11]	0 [-0.06;0.06]	**-0.74 [-0.85;-0.65]**	**-0.13 [-0.17;-0.09]**	-0.01 [-0.11;0.10]	0.12 [-0.02;0.26]	-0.16 [-0.30;0]	-0.03 [-0.12;0.07]	0.04 [-0.07;0.14]	**-0.34 [-0.60;-0.18]**
Chocolate & conf.	0.08 [-0.03;0.19]	0.01 [-0.03;0.04]	**0.16 [0.04;0.25]**	0.02 [-0.06;0.10]	0.07 [0;0.16]	**-0.27 [-0.40;-0.15]**	**-0.73 [-0.79;-0.67]**	0.02 [-0.09;0.15]	**0.19 [0.02;0.35]**	**0.49 [0.31;0.67]**	**-0.29 [-0.42;-0.16]**	**-0.35 [-0.46;-0.21]**	**-0.74 [-1.00;-0.48]**
Cake-type snacks	-0.12 [-0.27;0.04]	-0.04 [-0.10;0.02]	**0.24 [0.09;0.39]**	**0.14 [0.03;0.27]**	-0.09 [-0.20;0.01]	-0.19 [-0.35;0.02]	**-0.15 [-0.23;-0.09]**	**-0.71 [-0.85;-0.54]**	**0.27 [0.06;0.47]**	**-0.38 [-0.68;-0.11]**	0.10 [-0.05;0.29]	-0.07 [-0.23;0.11]	**-0.44 [-0.83;-0.12]**
Savoury snacks	0.02 [-0.07;0.09]	0.02 [-0.01;0.05]	-0.02 [-0.10;0.06]	0.02 [-0.05;0.08]	-0.03 [-0.10;0.02]	-0.02 [-0.11;0.07]	-0.03 [-0.08;0]	-0.07 [-0.16;0]	**-0.71 [-0.83;-0.59]**	0 [-0.14;0.12]	-0.02 [-0.10;0.07]	-0.02 [-0.10;0.07]	**-0.27 [-0.48;-0.09]**
Meat & fish	0.02 [-0.04;0.07]	0 [-0.02;0.02]	-0.01 [-0.06;0.04]	0.03 [-0.02;0.07]	**0.05 [0.01;0.09]**	**-0.07 [-0.13;-0.01]**	0.02 [0;0.05]	-0.01 [-0.06;0.06]	0.01 [-0.06;0.10]	**-0.80 [-0.90;-0.70]**	**-0.15 [-0.21;-0.08]**	**-0.11 [-0.17;-0.04]**	-0.10 [-0.22;0.02]
Dairy & eggs	0.02 [-0.01;0.06]	-0.01 [-0.02;0.01]	-0.01 [-0.05;0.03]	0.03 [0;0.07]	0.02 [-0.01;0.05]	0.02 [-0.02;0.07]	-0.02 [-0.04;0.01]	0.01 [-0.03;0.06]	-0.05 [-0.10;0.02]	-0.07 [-0.13;0]	**-0.86 [-0.90;-0.81]**	**-0.12 [-0.17;-0.08]**	-0.05 [-0.15;0.05]
Fruit & veg	-0.03 [-0.08;0.01]	0 [-0.02;0.02]	0.02 [-0.02;0.07]	-0.03 [-0.06;0.01]	-0.02 [-0.06;0.02]	0.01 [-0.04;0.07]	**-0.09 [-0.12;-0.07]**	**-0.08 [-0.13;-0.04]**	**0.13 [0.05;0.20]**	-0.08 [-0.16;0]	-0.03 [-0.09;0.02]	**-0.58 [-0.64;-0.53]**	**-0.15 [-0.26;-0.04]**
Rest food & drink	-0.01 [-0.04;0.04]	0.01 [-0.01;0.02]	**-0.05 [-0.1;-0.01]**	0.01 [-0.02;0.05]	0.02 [0;0.05]	**0.06 [0.01;0.10]**	**0.03 [0.01;0.05]**	**0.05 [0.01;0.09]**	-0.18 [-0.24;0]	**-0.12 [-0.18;-0.06]**	-0.04 [-0.08;0.01]	-0.03 [-0.08;0.01]	**-0.57 [-0.66;-0.48]**

Notes: Elasticities in bold indicate those where 95% confidence intervals do not include zero. Columns indicate the group of price change and rows indicate the group of demand change. High-sugar soft drinks: >8g of sugar/100ml; medium-sugar soft drinks: 5-8g of sugar/100ml; low-sugar soft drinks: <5g of sugar/100ml; other soft drinks: water, fruit juice with no added sugars and milk-based drinks.

For peer review only

Table 3. Price elasticities of demand in mid-income (annual household income £20,000-£49,000) sample (n=305,841)

	High-sugar soft drinks	Medium-sugar soft drinks	Low-sugar soft drinks	Other soft drinks	Alcohol	Biscuits & cookies	Chocolate & conf.	Cake-type snacks	Savoury snacks	Meat & fish	Dairy & eggs	Fruit & vegetables	Rest food & drink
High-sugar soft drinks	**-0.75**<br>**[-0.85;-0.64]**	**-0.04**<br>**[-0.09;-0.01]**	-0.06<br>[-0.16;0.04]	-0.07<br>[-0.17;0.02]	**-0.15**<br>**[-0.22;-0.06]**	0.05<br>[-0.07;0.18]	**-0.07**<br>**[-0.11;-0.02]**	-0.02<br>[-0.14;0.11]	**0.27**<br>**[0.10;0.42]**	**-0.36**<br>**[-0.53;-0.17]**	**0.20**<br>**[0.08;0.33]**	-0.10<br>[-0.22;0.03]	0.01<br>[-0.27;0.22]
Medium-sugar soft drinks	-0.20<br>[-0.43;0.05]	**-0.67**<br>**[-0.78;-0.57]**	**-0.51**<br>**[-0.74;-0.23]**	**-0.36**<br>**[-0.60;-0.15]**	-0.12<br>[-0.30;0.13]	-0.06<br>[-0.40;0.27]	**-0.10**<br>**[-0.20;-0.01]**	0.00<br>[-0.27;0.27]	**-0.48**<br>**[-0.94;-0.11]**	**1.24**<br>**[0.72;1.69]**	-0.19<br>[-0.44;0.08]	**0.41**<br>**[0.10;0.76]**	-0.06<br>[-0.77;0.55]
Low-sugar soft drinks	-0.01<br>[-0.08;0.06]	**-0.05**<br>**[-0.08;-0.02]**	**-0.85**<br>**[-0.92;-0.77]**	**-0.09**<br>**[-0.14;-0.02]**	**-0.23**<br>**[-0.28;-0.18]**	-0.01<br>[-0.09;0.07]	**-0.07**<br>**[-0.12;-0.04]**	0.00<br>[-0.08;0.10]	-0.07<br>[-0.20;0.04]	**0.13**<br>**[0.01;0.25]**	**0.16**<br>**[0.08;0.26]**	0.07<br>[-0.02;0.15]	-0.01<br>[-0.14;0.17]
Other soft drinks	**0.11**<br>**[0.03;0.22]**	**-0.06**<br>**[-0.10;-0.03]**	0.02<br>[-0.07;0.12]	**-0.79**<br>**[-0.87;-0.73]**	**-0.13**<br>**[-0.19;-0.07]**	0.03<br>[-0.08;0.14]	**-0.07**<br>**[-0.11;-0.04]**	-0.01<br>[-0.11;0.09]	0.03<br>[-0.10;0.18]	-0.13<br>[-0.25;0.06]	0.13<br>[0;0.22]	-0.04<br>[-0.14;0.06]	-0.02<br>[-0.23;0.20]
Alcohol	-0.04<br>[-0.12;0.03]	-0.02<br>[-0.05;0]	0.01<br>[-0.05;0.09]	**-0.13**<br>**[-0.20;-0.08]**	**-0.91**<br>**[-0.95;-0.85]**	-0.05<br>[-0.15;0.03]	0.03<br>[0;0.05]	**0.13**<br>**[0.05;0.21]**	**0.13**<br>**[0.01;0.23]**	0.04<br>[-0.08;0.17]	-0.04<br>[-0.11;0.04]	**-0.10**<br>**[-0.18;-0.02]**	**-0.29**<br>**[-0.46;-0.14]**
Biscuits & cookies	-0.01<br>[-0.08;0.07]	0<br>[-0.02;0.03]	**0.18**<br>**[0.11;0.25]**	0.05<br>[0;0.11]	**0.09**<br>**[0.02;0.13]**	**-0.67**<br>**[-0.75;-0.58]**	**-0.11**<br>**[-0.15;-0.08]**	**-0.11**<br>**[-0.18;-0.05]**	**0.14**<br>**[0.03;0.24]**	**-0.19**<br>**[-0.33;-0.08]**	-0.06<br>[-0.16;0.01]	-0.01<br>[-0.09;0.08]	**-0.45**<br>**[-0.62;-0.29]**
Chocolate & conf.	0.06<br>[-0.05;0.17]	0.01<br>[-0.02;0.04]	**0.21**<br>**[0.12;0.32]**	0.02<br>[-0.07;0.09]	0.07<br>[0;0.14]	-0.07<br>[-0.18;0.03]	**-0.74**<br>**[-0.79;-0.69]**	0.07<br>[-0.02;0.19]	**0.27**<br>**[0.12;0.42]**	**0.41**<br>**[0.24;0.58]**	**-0.30**<br>**[-0.42;-0.19]**	**-0.20**<br>**[-0.31;-0.09]**	**-1.03**<br>**[-1.32;-0.81]**
Cake-type snacks	0.12<br>[-0.01;0.26]	0.05<br>[0;0.09]	**0.15**<br>**[0.03;0.26]**	0.06<br>[-0.05;0.17]	0.01<br>[-0.09;0.09]	**-0.26**<br>**[-0.47;-0.13]**	**-0.16**<br>**[-0.22;-0.11]**	**-0.65**<br>**[-0.78;-0.49]**	**0.25**<br>**[0.08;0.45]**	**-0.24**<br>**[-0.42;-0.04]**	-0.02<br>[-0.17;0.11]	**-0.16**<br>**[-0.31;-0.01]**	**-0.55**<br>**[-0.82;-0.23]**
Savoury snacks	-0.02<br>[-0.08;0.03]	0.01<br>[-0.01;0.04]	0.03<br>[-0.04;0.08]	**-0.06**<br>**[-0.11;-0.01]**	-0.02<br>[-0.08;0.02]	-0.01<br>[-0.10;0.05]	-0.02<br>[-0.05;0.02]	-0.02<br>[-0.11;0.04]	**-0.75**<br>**[-0.84;-0.65]**	-0.05<br>[-0.16;0.05]	-0.01<br>[-0.09;0.07]	-0.01<br>[-0.09;0.07]	**-0.23**<br>**[-0.35;-0.10]**
Meat & fish	-0.01<br>[-0.05;0.03]	0.01<br>[0;0.03]	0.03<br>[-0.02;0.07]	0.01<br>[-0.03;0.04]	0.03<br>[0;0.06]	-0.05<br>[-0.10;0]	0<br>[-0.02;0.02]	**-0.05**<br>**[-0.11;-0.01]**	-0.06<br>[-0.14;0]	**-0.75**<br>**[-0.81;-0.67]**	**-0.12**<br>**[-0.17;-0.07]**	**-0.08**<br>**[-0.13;-0.02]**	0.04<br>[-0.05;0.14]
Dairy & eggs	0.03<br>[-0.01;0.06]	-0.01<br>[-0.02;0]	0.03<br>[-0.01;0.07]	**0.04**<br>**[0.01;0.06]**	0<br>[-0.02;0.03]	0.03<br>[-0.01;0.07]	-0.01<br>[-0.03;0.01]	-0.03<br>[-0.07;0.01]	-0.02<br>[-0.09;0.03]	-0.01<br>[-0.07;0.04]	**-0.89**<br>**[-0.94;-0.84]**	**-0.13**<br>**[-0.16;-0.08]**	**-0.09**<br>**[-0.19;-0.01]**
Fruit & veg	0.01<br>[-0.02;0.05]	0.01<br>[0;0.02]	-0.02<br>[-0.05;0.02]	-0.03<br>[-0.05;0]	0<br>[-0.03;0.02]	0.02<br>[-0.02;0.06]	**-0.06**<br>**[-0.08;-0.04]**	**-0.08**<br>**[-0.12;-0.05]**	0.05<br>[0;0.11]	**-0.12**<br>**[-0.18;-0.05]**	0.02<br>[-0.02;0.06]	**-0.58**<br>**[-0.62;-0.54]**	-0.04<br>[-0.12;0.04]
Rest food & drink	0<br>[-0.05;0.05]	0<br>[-0.01;0.02]	**-0.05**<br>**[-0.09;-0.03]**	**0.05**<br>**[0.03;0.08]**	-0.02<br>[-0.04;0.01]	0.01<br>[-0.03;0.05]	**0.03**<br>**[0.01;0.04]**	0<br>[-0.04;0.03]	**-0.11**<br>**[-0.15;-0.03]**	**-0.09**<br>**[-0.14;-0.04]**	0.02<br>[-0.01;0.06]	-0.02<br>[-0.05;0.01]	**-0.7**<br>**[-0.76; -0.63]**

Notes: Elasticities in bold indicate those where 95% confidence intervals do not include zero. Columns indicate the group of price change and rows indicate the group of demand change. High-sugar soft drinks: >8g of sugar/100ml; medium-sugar soft drinks: 5-8g of sugar/100ml; low-sugar soft drinks: <5g of sugar/100ml; other soft drinks: water, fruit juice with no added sugars and milk-based drinks.

For peer review only

Table 4. Price elasticities of demand in high-income (annual household income > £50,000) sample (n=94,444)

	High-sugar soft drinks	Medium-sugar soft drinks	Low-sugar soft drinks	Other soft drinks	Alcohol	Biscuits & cookies	Chocolate & conf.	Cake-type snacks	Savoury snacks	Meat & fish	Dairy & eggs	Fruit & veg	Rest food & drink
High-sugar soft drinks	**-0.60 [-0.76;-0.39]**	-0.02 [-0.10;0.05]	**-0.26 [-0.46;-0.06]**	-0.03 [-0.19;0.12]	**-0.18 [-0.30;-0.04]**	0.03 [-0.18;0.33]	**-0.08 [-0.14;-0.01]**	0.06 [-0.15;0.27]	**0.32 [0.03;0.58]**	**-0.73 [-1.05;-0.34]**	**0.31 [0.14;0.55]**	-0.05 [-0.27;0.16]	0.19 [-0.34;0.60]
Medium-sugar soft drinks	-0.19 [-0.63;0.40]	**-0.57 [-0.72;-0.34]**	0.01 [-0.42;0.46]	**-0.39 [-0.76;-0.02]**	-0.26 [-0.66;0.03]	**-0.75 [-1.23;-0.13]**	0.01 [-0.15;0.17]	-0.16 [-0.65;0.34]	-0.08 [-0.75;0.59]	**0.90 [0.24;1.70]**	0.29 [-0.14;0.88]	0.12 [-0.43;0.72]	0.09 [-1.19;0.91]
Low-sugar soft drinks	-0.01 [-0.13;0.11]	-0.01 [-0.07;0.03]	**-0.78 [-0.94;-0.64]**	**-0.23 [-0.33;-0.11]**	**-0.22 [-0.29;-0.11]**	-0.06 [-0.21;0.07]	**-0.05 [-0.11;-0.01]**	0.07 [-0.07;0.22]	0.05 [-0.15;0.23]	-0.15 [-0.43;0.07]	0.13 [-0.02;0.27]	**0.16 [0.02;0.30]**	0.14 [-0.15;0.41]
Other soft drinks	**0.13 [0.02;0.28]**	**-0.06 [-0.11;-0.02]**	-0.13 [-0.28;0.02]	**-0.83 [-0.96;-0.71]**	-0.09 [-0.20;0.01]	-0.02 [-0.17;0.15]	**-0.13 [-0.21;-0.07]**	0.09 [-0.05;0.25]	0.12 [-0.08;0.36]	-0.19 [-0.45;0.08]	0.01 [-0.14;0.18]	-0.11 [-0.27;0.11]	0.29 [-0.04;0.58]
Alcohol	-0.10 [-0.21;0.05]	-0.04 [-0.09;0]	-0.07 [-0.21;0.07]	-0.08 [-0.19;0.01]	**-0.82 [-0.93;-0.74]**	-0.12 [-0.29;0.03]	0.04 [0;0.09]	0.11 [-0.05;0.25]	0.01 [-0.18;0.22]	0.11 [-0.10;0.30]	0.00 [-0.15;0.15]	-0.01 [-0.17;0.12]	-0.33 [-0.61;0]
Biscuits & cookies	0 [-0.14;0.12]	0.02 [-0.02;0.06]	**0.15 [0.03;0.28]**	0.06 [-0.05;0.17]	0.06 [-0.03;0.15]	**-0.64 [-0.78;-0.50]**	**-0.11 [-0.16;-0.04]**	-0.07 [-0.23;0.07]	0.13 [-0.09;0.31]	**-0.19 [-0.43;-0.01]**	0.02 [-0.10;0.20]	-0.12 [-0.26;0.08]	-0.35 [-0.62;0.01]
Chocolate & conf.	**0.19 [0.01;0.36]**	0.05 [-0.02;0.13]	-0.01 [-0.19;0.23]	-0.01 [-0.15;0.15]	0.13 [0;0.27]	-0.18 [-0.39;0.01]	**-0.75 [-0.85;-0.66]**	**0.27 [0.06;0.42]**	**0.49 [0.24;0.72]**	**0.41 [0.11;0.73]**	**-0.30 [-0.48;-0.08]**	**-0.22 [-0.44;-0.03]**	**-1.10 [-1.44;-0.72]**
Cake-type snacks	**-0.22 [-0.50;-0.01]**	-0.08 [-0.16;0.02]	0.12 [-0.08;0.37]	-0.02 [-0.19;0.18]	0.12 [-0.05;0.30]	-0.25 [-0.52;0.02]	**-0.14 [-0.23;-0.04]**	**-0.53 [-0.74;-0.25]**	**0.70 [0.30;1.03]**	-0.24 [-0.63;0.20]	0.25 [-0.06;0.51]	0.04 [-0.23;0.32]	**-1.18 [-1.71;-0.60]**
Savoury snacks	0.03 [-0.07;0.16]	0.01 [-0.04;0.04]	-0.03 [-0.15;0.06]	-0.07 [-0.15;0.04]	-0.03 [-0.11;0.05]	-0.04 [-0.17;0.09]	**-0.07 [-0.13;-0.01]**	-0.05 [-0.15;0.07]	**-0.83 [-0.98;-0.68]**	-0.05 [-0.25;0.15]	0.08 [-0.06;0.21]	0.07 [-0.06;0.21]	-0.14 [-0.36;0.11]
Meat & fish	-0.01 [-0.10;0.06]	0 [-0.03;0.02]	0.01 [-0.07;0.09]	0 [-0.07;0.05]	-0.01 [-0.07;0.04]	0 [-0.07;0.08]	-0.01 [-0.04;0.03]	0.01 [-0.08;0.07]	-0.11 [-0.24;0.01]	**-0.70 [-0.81;-0.57]**	-0.06 [-0.14;0.03]	-0.04 [-0.12;0.05]	-0.02 [-0.17;0.14]
Dairy & eggs	0.02 [-0.05;0.07]	0 [-0.02;0.02]	0.01 [-0.04;0.06]	0.04 [-0.01;0.10]	-0.02 [-0.06;0.03]	-0.01 [-0.07;0.06]	-0.03 [-0.06;0]	0 [-0.05;0.08]	-0.02 [-0.12;0.08]	0.02 [-0.06;0.12]	**-0.93 [-1.01;-0.86]**	**-0.09 [-0.17;-0.03]**	**-0.23 [-0.39;-0.06]**
Fruit & veg	0 [-0.06;0.07]	-0.02 [-0.04;0]	0.01 [-0.06;0.07]	0.04 [-0.01;0.09]	-0.03 [-0.07;0.01]	**0.09 [0.02;0.15]**	**-0.06 [-0.10;-0.03]**	**-0.08 [-0.15;-0.01]**	0.03 [-0.06;0.12]	-0.08 [-0.19;0.03]	-0.03 [-0.11;0.06]	**-0.69 [-0.75;-0.58]**	0.07 [-0.12;0.21]
Rest food & drink	-0.02 [-0.05;0.09]	0.01 [-0.01;0.03]	0.01 [-0.04;0.07]	0.03 [-0.01;0.07]	-0.01 [-0.05;0.02]	0.05 [-0.02;0.11]	0.04 [0;0.06]	**-0.06 [-0.12;-0.01]**	**-0.1 [-0.18;-0.01]**	**-0.1 [-0.19;-0.01]**	-0.02 [-0.08;0.05]	-0.06 [-0.13;0]	**-0.7 [-0.83;-0.58]**

Notes: Elasticities in bold indicate those where 95% confidence intervals do not include zero. Columns indicate the group of price change and rows indicate the group of demand change. High-sugar soft drinks: >8g of sugar/100ml; medium-sugar soft drinks: 5-8g of sugar/100ml; low-sugar soft drinks: <5g of sugar/100ml; other soft drinks: water, fruit juice with no added sugars and milk-based drinks.

STROBE Checklist statement

Smith et al. "Reducing sugar consumption: are sweet snacks more sensitive to price increases than sugar-sweetened beverages?"

	Item No	Recommendation
**Title and abstract**	1	(*a*) Indicate the study's design with a commonly used term in the title or the abstract [abstract]
		(*b*) Provide in the abstract an informative and balanced summary of what was done and what was found [included]
**Introduction**		
Background/rationale	2	Explain the scientific background and rationale for the investigation being reported [reported on page 4]
Objectives	3	State specific objectives, including any prespecified hypotheses [reported on page 4]
**Methods**		
Study design	4	Present key elements of study design early in the paper [page 4]
Setting	5	Describe the setting, locations, and relevant dates, including periods of recruitment, exposure, follow-up, and data collection [page 4/5 where relevant; secondary data]
Participants	6	(*a*) Give the eligibility criteria, and the sources and methods of selection of participants
Variables	7	Clearly define all outcomes, exposures, predictors, potential confounders, and effect modifiers. Give diagnostic criteria, if applicable [demand model briefly explained in page 5, and explained in detail in technical appendix]
Data sources/ measurement	8*	For each variable of interest, give sources of data and details of methods of assessment (measurement). Describe comparability of assessment methods if there is more than one group [explained in text on page 5 or in/adjacent tables]
Bias	9	Describe any efforts to address potential sources of bias [described in brief in page 5, in technical appendix in detail]
Study size	10	Explain how the study size was arrived at [explained in page 4]
Quantitative variables	11	Explain how quantitative variables were handled in the analyses. If applicable, describe which groupings were chosen and why [explained in pages 4-5]
Statistical methods	12	(*a*) Describe all statistical methods, including those used to control for confounding [technical appendix]
		(*b*) Describe any methods used to examine subgroups and interactions [page 5]
		(*c*) Explain how missing data were addressed [page 4]
		(*d*) If applicable, describe analytical methods taking account of sampling strategy [n/a]
		(*e*) Describe any sensitivity analyses [n/a]
**Results**		
Participants	13*	(a) Report numbers of individuals at each stage of study—eg numbers potentially eligible, examined for eligibility, confirmed eligible, included in the study, completing follow-up, and analysed [n/a as secondary data]
		(b) Give reasons for non-participation at each stage [n/a]
		(c) Consider use of a flow diagram [n/a]
Descriptive data	14*	(a) Give characteristics of study participants (eg demographic, clinical, social) and information on exposures and potential confounders [table 1]

For peer review only - http://bmjopen.bmj.com/site/about/guidelines.xhtml

		(b) Indicate number of participants with missing data for each variable of interest [page 4 where relevant]
Outcome data	15*	Report numbers of outcome events or summary measures [tables 2-3]
Main results	16	(*a*) Give unadjusted estimates and, if applicable, confounder-adjusted estimates and their precision (eg, 95% confidence interval). Make clear which confounders were adjusted for and why they were included [figures 1-4, appendix 2, pages 6-7]
		(*b*) Report category boundaries when continuous variables were categorized [throughout tables]
		(*c*) If relevant, consider translating estimates of relative risk into absolute risk for a meaningful time period [n/a]
Other analyses	17	Report other analyses done—eg analyses of subgroups and interactions, and sensitivity analyses [pages 6-7]
**Discussion**		
Key results	18	Summarise key results with reference to study objectives [page 7]
Limitations	19	Discuss limitations of the study, taking into account sources of potential bias or imprecision. Discuss both direction and magnitude of any potential bias [page 7]
Interpretation	20	Give a cautious overall interpretation of results considering objectives, limitations, multiplicity of analyses, results from similar studies, and other relevant evidence [page 8]
Generalisability	21	Discuss the generalisability (external validity) of the study results [page 7]
**Other information**		
Funding	22	Give the source of funding and the role of the funders for the present study and, if applicable, for the original study on which the present article is based [page 3]

For peer review only

BMJ Open

Are sweet snacks more sensitive to price increases than sugar-sweetened beverages: analysis of British food purchase data

Journal:	*BMJ Open*
Manuscript ID	bmjopen-2017-019788.R2
Article Type:	Research
Date Submitted by the Author:	29-Jan-2018
Complete List of Authors:	Smith, Richard ; London School of Hygiene and Tropical Medicine, Faculty of Public Health and Policy<br>Cornelsen, Laura; London School of Hygiene and Tropical Medicine, Faculty of Public Health and Policy<br>Quirmbach, Diana; London School of Hygiene and Tropical Medicine, Faculty of Public Health and Policy<br>Jebb, Susan; Univerof Oxford, Primary Care Health Sciences<br>Marteau, Theresa; University of Cambridge, Behaviour and Health Research Unit
**Primary Subject Heading**:	Health economics
Secondary Subject Heading:	Public health
Keywords:	price elasticity, sweet snacks, sugar-sweetened beverages, fiscal policy

SCHOLARONE™ Manuscripts

Are sweet snacks more sensitive to price increases than sugar-sweetened beverages: analysis of British food purchase data

Richard D Smith (PhD) [1,2]*

Laura Cornelsen (PhD) [1]

Diana Quirmbach (PhD) [1,2]

Susan A. Jebb (PhD) [2,3]

Theresa Marteau (PhD) [2]

[1] Faculty of Public Health and Policy, London School of Hygiene and Tropical Medicine, 15-17 Tavistock Place, London WC1H 9SH, UK

[2] Behaviour and Health Research Unit, Institute of Public Health, University of Cambridge, Forvie Site, Robinson Way, Cambridge CB2 0SR, UK

[3] Nuffield Department of Primary Care Health Sciences, University of Oxford, Woodstock Rd, Oxford OX2 6GG, UK

*Corresponding author:

Richard.Smith@LSHTM.ac.uk

+44 (0)20 7927 2403

For peer review only

For peer review only - http://bmjopen.bmj.com/site/about/guidelines.xhtml

Abstract

Objectives

Taxing sugars-sweetened beverages (SSBs) is now advocated, and implemented, in many countries as a measure to reduce the purchase and consumption of sugar to tackle obesity. To date there has been little consideration of the potential impact that such a measure could have if extended to other sweet foods, such as confectionery, cakes and biscuits that contribute more sugar to the diet than SSBs. The objective of this study is to compare changes in the demand for sweet snacks and sugar-sweetened beverages arising from potential price increases.

Setting

Secondary data on household itemised purchases of all foods and beverages from 2012-2013.

Participants

Representative sample of 32,249 households in Great Britain.

Primary and secondary outcome measures

Change in food and beverage purchases due to changes in their own price and the price of other foods or beverages measured as price elasticity of demand for the full sample and by income groups.

Results

Chocolate and confectionery, cakes and biscuits have similar price sensitivity as SSBs, across all income groups. Unlike the case of SSBs, price increases in these categories are also likely to prompt reductions in the purchase of other sweet snacks and SSBs, which magnify the overall impact. The effects of price rises are greatest in the low-income group.

Conclusions

Policies that lead to increases in the price of chocolate and confectionery, cakes, and biscuits may lead to additional and greater health gains than similar increases in the price of SSBs through direct reductions in the purchases of these foods and possible positive multiplier effects that reduce demand for other products. Although some uncertainty remains, the associations found in this analysis are sufficiently robust to suggest that policies – and research – concerning the use of fiscal measures should consider a broader range of products than is currently the case.

Strengths and limitations of this study

- Detailed transaction level data on all food and beverage purchases collected electronically from a representative sample of >30,000 GB households over two years
- Transaction level data allows for separating and analysing demand for ready-to-consume sweet snacks
- Demand analysis accounts for zero-purchases and endogeneity of total food expenditure
- Data excludes purchases of foods and beverages bought and consumed outside homes
- Purchase data does not necessarily amount to consumption due to possible waste

For peer review only - http://bmjopen.bmj.com/site/about/guidelines.xhtml

Copyright
The Corresponding Author has the right to grant on behalf of all authors and does grant on behalf of all authors, a worldwide licence to the Publishers and its licensees in perpetuity, in all forms, formats and media (whether known now or created in the future), to i) publish, reproduce, distribute, display and store the Contribution, ii) translate the Contribution into other languages, create adaptations, reprints, include within collections and create summaries, extracts and/or, abstracts of the Contribution, iii) create any other derivative work(s) based on the Contribution, iv) to exploit all subsidiary rights in the Contribution, v) the inclusion of electronic links from the contribution to third party material where-ever it may be located; and, vi) licence any third party to do any or all of the above.

Competing interest statement
All authors have completed the ICMJE uniform disclosure form at http://www.icmje.org/coi_disclosure.pdf (available from the corresponding author) and declare: no support from any organisation for the submitted work; no financial relationships with any organisations that might have an interest in the submitted work in the previous three years, no other relationships or activities that could appear to have influenced the submitted work.

Author contributions
RS and TM conceived the study. DQ and LC conducted analyses, interpreted results and drafted the paper. RS, TM and SJ helped design the study, interpreted the results and drafted the paper. RS is guarantor of the study and affirms that the manuscript is an honest, accurate, and transparent account of the study being reported; that no important aspects of the study have been omitted; and that any discrepancies from the study as planned (and, if relevant, registered) have been explained.

Ethical approval
Ethical approval was not required as the data used in this study are secondary, anonymised purchase data. No patients were involved in the study.

Patient involvement

No patients or public were involved in this research.

Funding statement
This study was funded by the Department of Health in England Policy Research Programme (Policy Research Unit in Behaviour and Health (PR-UN-0409-10109)). LC is funded by an MRC Fellowship Grant (MR/P021999/1). Representatives of the Department of Health had no role in the data collection, analysis or interpretation, and no role in the study design or in writing the manuscript. The views expressed in this paper are those of the authors and not necessarily those of the Department of Health in England.

Data sharing statement
The data for this study were purchased from Kantar Worldpanel but its use is restricted to the persons named in the purchase contract which forbids the users to share the data with other potential (unnamed on the contract) users. Data access requests should be directed to Kantar Worldpanel.

For peer review only - http://bmjopen.bmj.com/site/about/guidelines.xhtml

Introduction

With the global prevalence of obesity and associated health risks continuing to increase,[1,2] health-related taxes have become an established policy option intended to reduce energy intake. Most of these have focussed on sugar-sweetened beverages (SSBs), due to their consistent association with energy intake, weight gain, risk of type-2 diabetes, as well as dental caries.[3] In the US, six local jurisdictions have a tax on sugary beverages implemented due to health concerns.[4] Mexico, Finland and France apply different levels of volumetric taxes on SSBs, Hungary has adopted a system of volumetric taxes from products exceeding specified levels of sugar, and Chile taxes drinks with high levels of sugar at a rate 8% higher in comparison to drinks containing less sugar.[4] More recently, Portugal and Catalonia (Spain) implemented a two-tiered tax on sugary drinks, the United Arab Emirates and Saudi Arabia introduced a 50% tax on carbonated drinks, and Brunei and Thailand introduced an excise duty on sugary drinks.[4] There are similar plans across a number of other countries such as Estonia, the Philippines, Indonesia, Israel and South Africa.[5] The UK government has confirmed an industry levy starting in April 2018 to incentivise producers to reformulate their products or, if not, to increase the price of SSBs.[6]

Research to date suggests that increasing the price of SSBs generates a small, but significant, reduction in their purchase (broadly, a 10% price rise reduces purchases by 6-8%), with a more pronounced effect in poorer households, and that substitution towards other soft-drink categories only minimally offsets the energy reductions achieved through decreases in SSBs.[7-18] However, there has been little research on the impact such a price increase could have on other contributors to sugar and energy intake, including alcohol[18] and sweet snack-foods (such as confectionery, cakes and biscuits). With the apparent success of fiscal measures to increase the price of SSBs, it would be useful to establish whether a similar, or possibly greater, effect on consumption of snack-foods could be obtained from a similar price change.

The research presented here is the first to provide a direct analysis of the relationship between price increases and demand for sweet snack foods, within the context of demand for soft- and alcoholic drink purchases, across different income groups.

Methods

The impact, or sensitivity, of demand for a product to price changes is termed the price elasticity of demand. This shows the percent change in the demand for product X if its own price changes (own-price elasticity) or the price of other products (Y, Z) changes (cross-price elasticity). These elasticities are estimated from demand models. We apply a partial demand model, which models household expenditure shares on prices of different products and total expenditure, adjusted for overall price level. The demand model we use is adapted from the common and widely applied Almost Ideal Demand System (AIDS).

The demand model and price elasticities are estimated from household expenditure data from January 2012 to December 2013, provided by Kantar Worldpanel. The data include information on household expenditures from a sample of British households (~36,000), representative of the population with respect to household size, number of children, social class, geographical region and age group, on food and drink purchases for home consumption made in a variety of outlets, including major retailers, supermarkets, butchers, greengrocers, and corner shops. The dataset consists of individual transactions, providing detailed information on the day of purchase, outlet, amount spent, volume purchased and also nutrient composition of each of the products, including

For peer review only - http://bmjopen.bmj.com/site/about/guidelines.xhtml

sugar. Households record all purchases (barcodes and the receipts) for products brought back into the home with handheld scanners at home. In addition, Kantar Worldpanel annually collects socio-demographic information for each household, such as household size and composition, income group, social class, tenure and geographical location (postcode district), as well as age, gender, ethnicity and highest educational classification of the main shopper. As we are interested in analysing the demand across income groups we excluded households (n=4,075) for which this variable is missing (due to households' preference to not report this).

The full dataset used in the analysis thus consists of 32,249 households, of which 80% appear in both years (25,535), providing ~75 million food and beverage purchases disaggregated at the brand and package level, capturing both cross-sectional and longitudinal variation in household purchases.

For analysis, data were aggregated from all foods and beverages into 13 distinct groups: (i) high-sugar soft drinks, containing more than 8g sugar/100 ml (assuming a dilution rate of 1:4 as used by the British Soft Drinks Association for concentrated SSBs); (ii) medium-sugar soft drinks, with between 5-8g sugar/100 ml; (iii) low-sugar soft drinks with less than 5g of sugar/100ml; (iv) other soft drinks, including fruit juices, milk-based drinks (excluding pure milk) and water[1]; (v) alcohol, including beer, lager, cider, wines and spirits; (vi) cookies, biscuits and cereal bars; (vii) chocolate and confectionary; (viii) cake-type snacks, including cake bars, pastries, muffins, flapjack and mince pies; (ix) savoury snacks, including crisps, popcorn, crackers and savoury assortments; (x) fresh and frozen meat and fish; (xi) dairy; (xii) fruit and vegetables; (xiii) rest of food and drink. Sweet snack foods – defined as foods which are at ambient temperature and able to be consumed on the go without utensils – were the most disaggregated as these were the focus for this study.

As many beverages and snack foods are storable and not purchased very frequently, data were aggregated at 4-week intervals for each household, providing a total of n=623,459 household-month observations. As the data are aggregated to 4-weekly periods (n=26) and into thirteen groups, we estimate geographical price indices from transaction prices of each individual product, based on the postcode area the households reside (see appendix 1 for further details).

Even at this level of aggregation, a substantial amount of zero-expenditure months remain, as most households do not buy beverages or foods from every category every month and some households never buy certain categories during the whole sample period. A two-step procedure was followed to take account of this censoring of the dependent variable in the estimation strategy. The AIDS approach was adapted for the panel data context to allow control for unobserved household heterogeneity via a fixed effects specification. The full specification, including the procedures for handling censoring, endogeneity of prices and total expenditure, and estimation of price elasticities is provided in appendix 1.

Due to potential differences in purchasing behaviour, the analyses are carried out in the full sample and in subsamples by household annual income (low-income (< £20,000), mid-income (£20,000 - £49,000) and high-income (>£50,000+).

Results

[1] The categorisation of the non-alcoholic beverages follows the structure in the proposed levy for sugary drinks producers in the UK (effective April 2018)[6] separating drinks that would be levied at higher rate of £0.24 per L for drinks containing more than 8g of sugar per 100ml), at a lower rate of £0.18 per L (drinks containing between 5-8g of sugar/100ml) and not levied (drinks <5g sugar/100ml) and remaining soft drinks (juice with no added sugars, milk-based drinks and water).

For peer review only - http://bmjopen.bmj.com/site/about/guidelines.xhtml

Table 1 presents the socio-demographic profile of the sample. A comparison of Kantar Worldpanel with representative household data from the Living Cost and Food survey (LCF)[2] has found the sociodemographic and regional profiles of the samples to match well, although our sample has a slightly higher share of (i) low-income households, (ii) households that own a computer and/or a car, and (iii) households in the South and Southeast of England.[19]

TABLE 1 ABOUT HERE

Table 2 (top panel) presents the average sugar content across the food and beverage groups as well as total purchases of sugar (expressed as grams per person per day) that are purchased and brought home (i.e. excluding purchases consumed outside homes), across each of the categories outlined above and split by income level. There is a clear income gradient: those on lower-incomes purchase more sugar per person per day. It is also clear that more sugar is consumed across all income groups from sweet snacks (17.1g) than all beverages combined (alcoholic and non-alcoholic) (13.9g). In comparison to SSBs in particular (6.9g), sweet snacks combined contribute more than twice the amount of sugar. It is also evident that sweet snacks have per 100g a considerably higher sugar content in comparison to 100ml of beverages.

The bottom panel of table 2 shows the share of households that purchase products from each of the food groups during the 26 4-week periods. A higher share of non-purchases (e.g. only 13% of households purchase medium-sugar soft drinks across the periods) has implications for methodology which are discussed in appendix, but also provides an overview of the regularity of purchases. Approximately half of the households (49%) purchase high-sugar soft drinks across the 26 4-week periods. Low-sugar soft drinks are bought more frequently (69% of observations are positive across household-periods). In comparison, cookies and biscuits as well as chocolate and confectionary are bought more frequently (77% and 69%) and cake-type snacks are bought less frequently (37%). In comparison to low- and high-income households, middle-income households have a slightly higher frequency of purchase of high-sugar soft drinks and sweet snacks.

TABLE 2 ABOUT HERE

Table 3 presents total expenditure, expenditure shares and average prices across all households and split into three income groups. The critical aspect for analysis here is the expenditure share, where there is a marked income gradient with respect to expenditure on beverages, and a slightly lower gradient for sweet snacks. The low-income group spend 14% of total drink expenditure on the high- and medium-sugar soft drinks, compared with 12% and 10% for medium- and high-income groups, respectively. Similarly, of the total food expenditure, sweet snacks represent 7%, 7% and 6% among the low-, medium- and high-income groups, respectively.

TABLE 3 ABOUT HERE

The full results of the unconditional, uncompensated own- and cross-price elasticities are presented in appendix 2. In sum, the own-price elasticity for alcoholic drinks is higher than for all other categories; that is, alcoholic drinks are more sensitive to price change than any other category. Elasticities for all categories are inelastic (i.e. smaller than 1); this means that there is a less than proportionate decrease in purchase following a price rise for products, indicating that price increases reduce demand for all products, although with differing strength of effect. This pattern is seen across all income groups, with relatively similar absolute elasticity values. Comparing SSB and sweet

[2] LCF is a survey of household spending and the cost of living in the UK reflecting household budgets and is conducted by the UK Office for National Statistics.

For peer review only - http://bmjopen.bmj.com/site/about/guidelines.xhtml

snack price sensitivity, the elasticity for SSB is on average -0.77 (a 10% increase in price yields a 7.7% reduction in quantity purchased) whereas for chocolate and confectionary it is -0.74, biscuits -0.69 and cakes -0.66. There is relatively little variance across income groups in the own-price elasticity for chocolate and confectionery whereas for biscuits and cookies and cake-type snacks, low-income households are relatively more price responsive (-0.74 and -0.71, respectively in comparison to -0.64 and -0.53 in high-income group). Sweet snack foods, overall, thus appear to have only slightly lower level of price sensitivity in comparison to SSBs.

Of interest also is the impact on purchases across other aspects of the diet when the price of SSBs or sweet snacks increases. Figures 1 to 4 present the impacts on purchases as a result of a 1% increase in price of each of the soft-drink and snack categories, to illustrate the variance in these effects (presenting only those effects where confidence intervals exclude zero). This is presented for the total sample (figure 1) and then for each income group (figures 2-4).

FIGURES 1-4 ABOUT HERE

In aggregate across all income groups, (figure 1) clear differences arise from increasing the price of SSBs compared with sweet snacks. Increases in the price of high-sugar soft drinks are associated with a decrease in purchases of medium-sugar soft drinks (2.5% reduction in purchase if the price of high-sugar drinks increases by 10%) but increased purchases of other soft-drinks (1.1%), and chocolate and confectionary (0.08%). Increasing the price of diet/low-sugar drinks elicits greater reaction in other soft drink purchases (1.1% decrease in purchase of high-sugar drinks and 2.8% decrease in purchase of medium-sugar drinks for a 10% increase in price of low-sugar drinks) but also some increase in demand for cakes, biscuits and chocolate (1.3-1.7%). Increasing the price of medium-sugar soft drinks, however, only reduces demand for other soft drinks (by 0.5%), low-sugar soft drinks (0.3%) and alcohol (0.3%) with no associations observed with demand for snacks.

For sweet snacks, there are considerably more complementary effects, with significant reductions in other categories. A price increase for chocolate and confectionary items is associated with small but significant decreases across all soft-drinks (reductions in purchase of 0.6-0.8% for a 10% price increase) as well as biscuits and cakes (by 1.2%), and savoury snacks (1.6%). For biscuits, there are significant reductions in the demand for cakes (2.3%) as well as chocolate and confectionary (1.7%). Finally, for a price increase in cakes there are smaller changes, with reductions in purchases of biscuits (by 0.7%) but increases in the purchase of chocolate and confectionary (0.7%), and alcohol (0.8%). Thus, increasing the price of chocolate snacks especially elicits a range of significant reductions in purchases across most categories.

Although many of the associations at the aggregate level are replicated across income groups (figures 2-4), there is some clear variance by income group. An increase in the price of sugary-drinks is associated with a reduction in medium-sugar drinks only within the low-income group (by 3% if price increases by 10%) while an increase in other soft-drinks is observed in medium- and high-income groups (1%). Furthermore, in the high-income group a higher SSB price leads to an increase in purchases of chocolate and confectionary (1-2%) but also a reduction in purchases of cake-type snacks (2% , albeit all with relatively large confidence intervals).

Increasing the price of diet/low sugar drinks seems to be associated with more substitute relationships, with significant increases in sweet snack demand (1-2% increase to a price increase of 10%), especially for low- and medium-income groups. However, for increases in the price of sweet snacks the differences are more marked. Increasing the price of biscuits generates complementary reductions in the purchase of chocolate and confectionary for the low-income group (by 3% if price

For peer review only - http://bmjopen.bmj.com/site/about/guidelines.xhtml

increases by 10%), reductions in cake-type snacks for the middle-income group (3%), but no such reductions for the high-income group where a reduction in medium-sugar drinks is observed instead (8%). While a relatively large change, the absolute change would be small as the share of mid-sugar drinks in overall expenditure is very small.

Changes in the price of cake-type snacks has limited impact on other categories for those in the low-income group, but for the middle-income group it reduces purchase of biscuits (1%), but is also associated with a slight increase in purchase of alcohol (1%). For the high-income group this effect is even more pronounced, with increases in purchase of alcohol (1%) and chocolate as substitutes (3%). Increasing the price of chocolate and confectionary has a similar effect across all income groups, with associated reductions in the purchase of most other food and drink categories (1-2% if price increases by 10%).

Discussion

The price elasticity of chocolate & confectionery was highest among the sweet snacks and is almost identical to that for SSBs (although both are lower than alcohol). Further, price increases in SSBs are associated with an increase in purchase of other soft-drinks and chocolate and confectionary, whereas an increase in the price of chocolate is associated with a reduction in purchase of SSBs, as well as a range of other snacks. The differences across food categories, and income groups, indicates the complexity of estimating the impact of a single price increase. Nonetheless, it does suggest that policies to increase the price of sweet snacks could have a greater impact than that seen thus far for SSBs, not least because chocolate and confectionery alone contribute a similar quantity of sugar per person per day as SSBs in our sample. Moreover this analysis suggests they have stronger associations with reductions in other categories of foods and SSBs (i.e. complementary relationships), creating a cumulative positive multiplier effect. This appears to be most pronounced in the low- and middle-income groups, as would be expected. The strength of these results suggests that further research is warranted to analyse the impact on diet composition and model the long-term impacts of such interventions on health outcomes.

The extent to which a levy on sugary snacks could yield a lower consumption of sugar is, of course, dependent on the structure of the levy, but considering the relatively high sugar content of these foods (per 100g) even a small levy based on sugar content is likely to change prices, assuming it is passed through. Whether a multi-tiered levy based on sugar content, such as proposed for the sugary drinks, would encourage reformulation is another question since there are important differences in the ease of reformulation compared to SSBs and less is known about consumer acceptability of the reformulated snack food products.

Overall, our estimates of price-elasticity for foods and sugary beverages are consistent with the literature. Meta-analyses of price elasticity in broad food groups in high-income countries find these to range between -0.4 to -0.8 and that of sweets, confectionery and sweetened beverages at -0.6.[7,20] Our estimates range between -0.6 to -0.8 but we also use greater disaggregation of food and beverage groups. Another study reports the meta-estimate of price-elasticity of SSBs to be -1.3 that is higher than our estimate of -0.77, however the meta-estimate includes studies from Mexico and Brazil and price-elasticity is dependent on income levels and lower-income populations are likely to have greater responsiveness to price changes (i.e. smaller elasticity value) as they spend a greater proportion of their incomes on food and beverages.[21] Two studies from Chile, also suggest somewhat more responsive demand (SSBs: -1.3 to -1.4, sweets and desserts -0.8 to -1.2).[22-23]

For peer review only - http://bmjopen.bmj.com/site/about/guidelines.xhtml

Elsewhere, a US study found, as here, a substitution effect towards juice and milk and a reduction in diet beverages if the price of SSBs increases. This study also estimated price-elasticity for SSBs at -0.8 and a somewhat less price responsive demand for sweets and sugars than our analysis (-0.3).[24] It has to be noted however, that we cannot impose *a priori* expectations for underlying preferences for foods and beverages to be the same in different populations and over time so some variance in elasticity estimates would be natural even if methods applied by the studies are similar.

There are, of course, limitations to the analysis presented here. The data, although large, representative and detailed, may be subject to under-recording; an issue present in all types of survey data. For instance, Kantar Worldpanel data appears to have lower levels of recorded alcohol expenditure than the Living Cost and Food survey.[19] The data also includes foods and beverages purchased and brought home and thus excludes all purchases that are consumed outside the home which are likely to be higher among more affluent households. Furthermore, the price responsiveness is based on price variations occurring in the market. This implies that any likely effect of the taxes inferred from these elasticities is subject to bias if the taxes, when implemented, have an impact on the demand beyond the direct price change.

Regardless of the models used, estimating demand requires a number of assumptions (see appendix 1), which may have influenced the estimates. We prioritised an approach that allowed controlling for unobservable household heterogeneity, including in the preferences towards different types of drinks and snacks, while also adjusting for non-purchase and endogeneity issues. Overall, own-price elasticities are estimated with greater robustness as an *a priori* expectation of an inverse relationship with price exists and own-price changes have a noticeable impact on purchases. However, the estimation of cross-price elasticities (substitution or complementarity effects) across products are harder to capture, as these are generally much smaller and the direction cannot be assumed *a priori*.[25] As most of cross-price elasticities are estimated close to zero, even small changes in methods can possibly affect the direction and thus interpretation of the effect. In addition, price elasticities are interpreted individually (i.e. allowing one price change at a time) but categories defined in this study might be taxed simultaneously (e.g. high- and medium-sugar soft drinks) which means that the policy impact may vary. Perhaps more critically, although this analysis can highlight significant relationships between products purchased, it cannot explain why these relationships exist. This requires further primary research and research within population subgroups.

Conclusion

Increasing the price of SSBs has become an accepted policy to reduce sugar intake. Analysis presented here based on data from Britain suggests that extending fiscal policies to include sweet snacks could lead to larger public health benefits, both directly by reducing purchasing and therefore consumption of these foods, and indirectly by reducing demand for other snack foods and indeed SSBs. Although some uncertainty remains, the associations observed in this analysis are sufficiently robust to suggest that policies – and research – concerning the use of fiscal measures to reduce intake of free sugars and improve diet quality should consider extending beyond SSBs to include the more frequently consumed sugar-based snacks including cakes, biscuits and, especially, chocolate and confectionary.

For peer review only - http://bmjopen.bmj.com/site/about/guidelines.xhtml

References

1. OECD. Obesity Update. Paris: OECD; 2014.

2. WHO. Obesity and overweight fact sheet [Internet]. WHO. 2016 [cited 2016 Jun 28]. Available from: http://www.who.int/mediacentre/factsheets/fs311/en/

3. SACN. Carbohydrates and Health [Internet]. London: The Scientific Advisory Committee on Nutrition; 2015 [cited 2016 Aug 17]. Available from: https://www.gov.uk/government/publications/sacn-carbohydrates-and-health-report

4. WCRF. NOURISHING Framework - Use of economic tools to address food affordability, purchase and incentives - Health-related food taxes [Internet] World Cancer Research Fund [cited 2017 Dec 01]. Available from http://www.wcrf.org/int/policy/nourishing-framework.

5. Public Health England. Sugar Reduction: The evidence for action Annexe 2: A mixed method review of behaviour changes resulting from experimental studies that examine the effect of fiscal measures targeted at high sugar food and non-alcoholic drink. London: Public Health England; 2015 Oct.

6. UK Department of Health. Childhood obesity: a plan for action [Internet]. London; 2016 Aug [cited 2016 Aug 22]. Available from: https://www.gov.uk/government/publications/childhood-obesity-a-plan-for-action

7. Cornelsen L, Green R, Turner R, Dangour AD, Shankar B, Mazzocchi M, et al. What Happens to Patterns of Food Consumption when Food Prices Change? Evidence from A Systematic Review and Meta-Analysis of Food Price Elasticities Globally. Health Econ. 2015 Dec 1;24(12):1548–59.

8. Zhen C, Finkelstein EA, Nonnemaker J, Karns S, Todd JE. Predicting the Effects of Sugar-Sweetened Beverage Taxes on Food and Beverage Demand in a Large Demand System. Am J Agric Econ. 2014 Jan 1;96(1):1–25.

9. Briggs AD, Mytton OT, Madden D, O'Shea D, Rayner M, Scarborough P. The potential impact on obesity of a 10% tax on sugar-sweetened beverages in Ireland, an effect assessment modelling study. BMC Public Health. 2013;13:860.

10. Briggs ADM, Mytton OT, Kehlbacher A, Tiffin R, Elhussein A, Rayner M, et al. Health impact assessment of the UK soft drinks industry levy: a comparative risk assessment modelling study. The Lancet Public Health. 2017 Jan;2(1):e15–22.

11. Colchero MA, Popkin BM, Rivera JA, Ng SW. Beverage purchases from stores in Mexico under the excise tax on sugar sweetened beverages: observational study. BMJ. 2016 Jan 6;352:h6704.

12. Colchero MA, Salgado JC, Unar-Munguía M, Hernández-Ávila M, Rivera-Dommarco JA. Price elasticity of the demand for sugar sweetened beverages and soft drinks in Mexico. Economics & Human Biology. 2015 Dec;19:129–37.

13. Dharmasena S, Capps O. Intended and unintended consequences of a proposed national tax on sugar-sweetened beverages to combat the U.S. obesity problem. Health Econ. 2012 Jun 1;21(6):669–94.

14. Paraje G. The Effect of Price and Socio-Economic Level on the Consumption of Sugar-Sweetened Beverages (SSB): The Case of Ecuador. PLOS ONE. 2016 Mar 30;11(3):e0152260.

For peer review only

15. Smith TA, Lin B-H, Lee J-Y. Taxing caloric sweetened beverages: potential effects on beverage consumption, calorie intake, and obesity. USDA-ERS Economic Research Report [Internet]. 2010 [cited 2016 Sep 14];(100). Available from: http://papers.ssrn.com/sol3/papers.cfm?abstract_id=2118636.

16. Tiffin R, Kehlbacher A, Salois M. The Effects of A Soft Drink Tax in the UK. Health Econ. 2015 May 1;24(5):583–600.

17. Zhen C, Wohlgenant MK, Karns S, Kaufman P. Habit Formation and Demand for Sugar-Sweetened Beverages. Am J Agr Econ. 2011 Jan 1;93(1):175–93.

18. Quirmbach D, Cornelsen L, Jebb SA, Marteau T, Smith R. Effect of increasing the price of sugar-sweetened beverages on alcoholic beverage purchases: an economic analysis of sales data. J - Epidemiol Community Health; doi:10.1136/jech-2017-209791.

19. Leicester A. How might in-home scanner technology be used in budget surveys? [Internet]. Institute for Fiscal Studies; 2012 [cited 2017 Feb 20]. Report No.: W12/01. Available from: https://ideas.repec.org/p/ifs/ifsewp/12-01.html.

20. Green R, Cornelsen L, Dangour AD, Turner R, Shankar B, Mazzocchi M, Smith RD. The Effect of Rising Food Prices on Food Consumption: systematic review with meta-regression. BMJ 2013; 346:f3703,p1-9.

21. Cabrera Escobar MA, Veerman LJ, Tollman SM, Bertram MY, Hofman KJ. Evidence that a tax on sugar sweetened beverages reduces the obesity rate: a meta-analysis. BMC Pub Health 2013, 13:1072, p1-10.

22. Guerrero-Lopez CM, Unar-Munguia M, Colchero MA. Price elasticity of the demand for soft drinks, other sugar-sweetened beverages and energy dense foods in Chile. BMC Pub Health 2017, 17: 180, p 1-8.

23. Caro JC, Ng SW, Smith Taillie L, Popking BM. Designing a tax to discourage unhealthy food and beverage purchases: The case of Chile. Food Policy 2017; 71:86-100.

24. Andreyeva T, Long, MW, Brownell KD. The Impact of Food Pirces on Consumption: A Systematic Review of Research on the Price Elasticity of Demand for Food. Am J Public Health. 2010; 100:216-222.

25. Cornelsen L, Mazzocchi M, Green R, Dangour AD, Smith RD. Estimating the Relationship between Food Prices and Food Consumption—Methods Matter. Appl Econ Perspect Policy. 2016 Sep 1;38(3):546–61.

For peer review only - http://bmjopen.bmj.com/site/about/guidelines.xhtml

Tables

Table 1 Demographic characteristics of estimation sample

	All households	Low-income	Mid-income	High-income
Number of households	32,249	11,580	15,816	4,853
Number of observations	623,459	223,174	305,841	94,444
Household size (SD)	2.7 (1.3)	2.3 (1.3)	2.9 (1.3)	3.2 (1.2)
Age of main shopper (SD)	47.8 (15.3)	52.4 (17.0)	46.0 (14.3)	42.9 (10.8)
Number of children if have children (SD)	1.7 (0.8)	1.8 (0.9)	1.8 (0.9)	1.7 (0.8)
Share of households that have children	0.4 (0.5)	0.3 (0.5)	0.4 (0.5)	0.5 (0.5)
**Social grade**	%			
Class A&B (highly skilled)	20.2	5.7	20.9	52.5
Class C1	37.5	30.5	43.0	36.2
Class C2	18.0	15.6	22.4	9.2
Class D	13.9	22.0	11.7	1.7
Class E (unskilled)	10.4	26.2	1.9	0.3
**Highest qualification**	%			
Degree or higher	24.1	11.6	25.9	47.8
Higher education	13.5	11.6	15.2	12.1
A Level	11.6	10.0	13.2	10.6
Secondary education (GCSE)	18.8	22.2	18.8	10.8
Other	7.6	11.6	6.0	3.1
None	7.6	15.2	4.1	0.9
Unknown	16.8	17.9	16.7	14.6
**Tenure**	%			
Owned outright	24.2	29.5	22.8	16.2
Mortgaged	40.0	17.1	47.6	69.7
Rented	29.7	46.4	23.6	9.8
Other	1.5	1.8	1.4	0.8
Unknown	4.7	5.2	4.7	3.6

Notes: Low income < £20,000 per year; mid-income £20,000 - £ 49,000; high-income >£50,000+; GCSE – General Certificate of Secondary Education

For peer review only - http://bmjopen.bmj.com/site/about/guidelines.xhtml

Table 2 Purchases of sugar (g) per person and day in 2013 and share (%) of non-zero observations across the food groups

		Average sugar content [1]	All households	Low-income	Mid-income	High-income
	Food group	***g (SD)***	***Total sugar purchased per day per person (g)*** [2]			
SSB	High-sugar soft drinks	10.4 (1.7)	6.3	7.6	6.8	4.5
	Medium-sugar soft drinks	6.5 (0.8)	0.6	0.7	0.6	0.4
	Low-sugar soft drinks	1.0 (1.4)	1.1	1.2	1.2	0.9
	Other soft drinks (incl. milk-based)	7.5 (4.7)	3.9	3.8	4.2	4.0
	Alcohol	1.4 (1.9)	2.0	2.2	2.3	1.6
Sweet snacks	Biscuits & cookies (incl. cereal fruit bars)	29.8 (10.5)	7.1	8.8	7.3	4.6
	Chocolate & confectionary	48.7 (11.9)	7.7	9.9	7.7	5.2
	Cake-type snacks	19.9 (11.4)	2.3	2.8	2.2	1.5
	Savoury snacks	5.2 (8.1)	0.6	0.7	0.6	0.5
	Fresh & frozen unprocessed meat, fish	1.0 (1.8)	0.5	0.6	0.6	0.4
	Dairy & eggs	4.2 (5.0)	15.7	19.6	15.9	11.4
	Fruit & Vegetables	6.2 (7.3)	17.6	20.7	17.9	14.2
	Rest food & drink	13.2 (19.2)	57.8	74.2	57.4	39.4
	Total		123.2	152.8	124.6	88.5
	Food group		***% of households that purchased products across the 4-week periods (non-zero observations)***			
SSB	High-sugar soft drinks		49%	45%	51%	48%
	Medium-sugar soft drinks		13%	13%	14%	14%
	Low-sugar soft drinks		69%	64%	72%	72%
	Other soft drinks (incl. milk-based)		55%	47%	58%	65%
	Alcohol		51%	43%	54%	59%
Sweet snacks	Biscuits & cookies (incl. cereal fruit bars)		77%	76%	78%	74%
	Chocolate & confectionary		69%	69%	70%	67%
	Cake-type snacks		37%	37%	38%	35%
	Savoury snacks		80%	75%	82%	82%
	Fresh & frozen unprocessed meat, fish		91%	89%	92%	92%
	Dairy & eggs		99%	99%	99%	99%
	Fruit & Vegetables		97%	96%	98%	98%
	Rest food & drink		99%	99%	99%	99%

Notes: SSB – sugar sweetened beverages; High-sugar soft drinks: >8g of sugar/100ml; medium-sugar soft drinks: 5-8g of sugar/100ml; low-sugar soft drinks: <5g of sugar/100ml; other soft drinks: water, fruit juice with no added sugars and milk-based drinks. [1] Average sugar content per 100g/100ml or item/unit (cake-type snacks and chocolate & confectionery) as reported in data. [2] Sugar purchases per person across the food groups are based on full data set of 2013 only (n=32,620), aggregated first to total GB using weights provided by Kantar Worldpanel and divided by number of persons (total GB and by income groups) and days in a year. Total GB population figures are based on Kantar Worldpanel estimates of the number of households in income brackets, taking into account the share of households of different sizes (1, 2, 3 or 4 members and for households that had 5 or more members we used an average size of 5). Total GB population estimate (2013): ~59.5m, from which 27% are in households with annual income <£20,000 (low-income), 40% are in households with income £20,000 - £49,000 (mid-income) and 17% are in

For peer review only - http://bmjopen.bmj.com/site/about/guidelines.xhtml

households with income >£50,000 (high-income). Households for which income is unknown or unanswered are excluded (14%).

For peer review only

For peer review only - http://bmjopen.bmj.com/site/about/guidelines.xhtml

Table 3 Mean total expenditure, expenditure shares and prices

		All households (n=623,459)		Low-income (n=223,174)		Mid-income (n=305,841)		High-income (n=94,444)	
		**Mean**	**SD**	**Mean**	**SD**	**Mean**	**SD**	**Mean**	**SD**
	Total 4-weekly expenditure (£)	183.5	110.6	155.0	96.3	194.1	112.2	211.9	121.3
	Expenditure share								
SSB	High-sugar soft drinks	0.015	0.028	0.015	0.032	0.015	0.027	0.013	0.015
	Medium-sugar soft drinks	0.002	0.008	0.002	0.009	0.002	0.008	0.002	0.002
	Low-sugar soft drinks	0.023	0.033	0.022	0.033	0.024	0.032	0.026	0.023
	Other soft drinks	0.016	0.026	0.013	0.025	0.016	0.025	0.020	0.028
	Alcohol	0.079	0.125	0.071	0.127	0.083	0.126	0.087	0.124
Sweet snacks	Biscuits & cookies (incl. cereal fruit bars)	0.025	0.029	0.026	0.031	0.025	0.028	0.022	0.026
	Chocolate & confectionary	0.028	0.041	0.031	0.045	0.027	0.038	0.024	0.037
	Cake-type snacks	0.006	0.012	0.007	0.014	0.006	0.011	0.005	0.010
	Savoury snacks	0.029	0.030	0.028	0.032	0.029	0.030	0.028	0.028
	Fresh & frozen unprocessed meat, fish	0.129	0.092	0.122	0.095	0.130	0.090	0.137	0.092
	Dairy & eggs	0.131	0.068	0.136	0.073	0.129	0.065	0.125	0.063
	Fruit & vegetables	0.130	0.088	0.124	0.090	0.129	0.085	0.142	0.088
	Rest food & drink	0.389	0.120	0.403	0.127	0.385	0.116	0.370	0.114
	All drinks	0.134		0.123		0.140		0.147	
	All food	0.866		0.877		0.860		0.853	
	% of drinks expenditure spent on SSB	12%		14%		12%		10%	
	% of food expenditure spent on sweet snacks	7%		7%		7%		6%	
	Price per volume unit (L, Kg)[1]	**Mean**	**SD**	**Mean**	**SD**	**Mean**	**SD**	**Mean**	**SD**
SSB	High-sugar soft drinks	0.92	0.74	0.91	1.06	0.92	1.06	0.93	1.07
	Medium-sugar soft drinks	0.95	0.49	0.95	1.17	0.95	1.18	0.97	1.18
	Low-sugar soft drinks	0.69	0.50	0.69	1.10	0.69	1.10	0.71	1.11
	Other soft drinks	0.86	1.08	0.86	1.08	0.86	1.08	0.87	1.08
	Alcohol	4.67	1.13	4.65	1.13	4.67	1.13	4.75	1.13
Sweet snacks	Biscuits & cookies (incl. cereal fruit bars)	3.77	1.07	3.76	1.06	3.77	1.07	3.80	1.07
	Chocolate & confectionary	0.77	1.33	0.77	1.33	0.77	1.33	0.78	1.33
	Cake-type snacks	1.00	1.06	0.99	1.06	1.00	1.06	1.00	1.06
	Savoury snacks	6.46	5.39	6.44	1.04	6.46	1.04	6.51	1.05
	Fresh & frozen unprocessed meat, fish	5.65	4.62	5.62	1.06	5.65	1.06	5.71	1.07
	Dairy & eggs	0.98	0.78	0.98	1.07	0.98	1.07	0.99	1.07
	Fruit & vegetables	1.66	1.30	1.65	1.09	1.66	1.09	1.69	1.10
	Rest food & drink	2.26	1.91	2.25	1.05	2.26	1.06	2.29	1.06

Notes: SSB – sugar sweetened beverages; [1] average unit prices (£) over geographical areas (n=110); volume of cakes and chocolate & confectionery is measured by items; Low income < £20,000 per year; mid-income £20,000 - £ 49,000; high-income >£50,000+; High-sugar soft drinks: >8g of sugar/100ml; medium-sugar soft drinks: 5-8g of sugar/100ml; low-sugar soft drinks: <5g of sugar/100ml; other soft drinks: water, fruit juice with no added sugars and milk-based drinks.

For peer review only - http://bmjopen.bmj.com/site/about/guidelines.xhtml

Figure Legends

Figure 1. Change in demand (%) as a response to 1% price increase in soft drinks and sweet snacks (all households n=623,459)

Figure 2. Change in demand (%) as a response to 1% price increase in soft drinks and sweet snacks (low-income households n=223,174)

Figure 3. Change in demand (%) as a response to 1% price increase in soft drinks and sweet snacks (mid-income households n=305,841)

Figure 4. Change in demand (%) as a response to 1% price increase in soft drinks and sweet snacks (high-income households n=94,444)

For peer review only

For peer review only - http://bmjopen.bmj.com/site/about/guidelines.xhtml

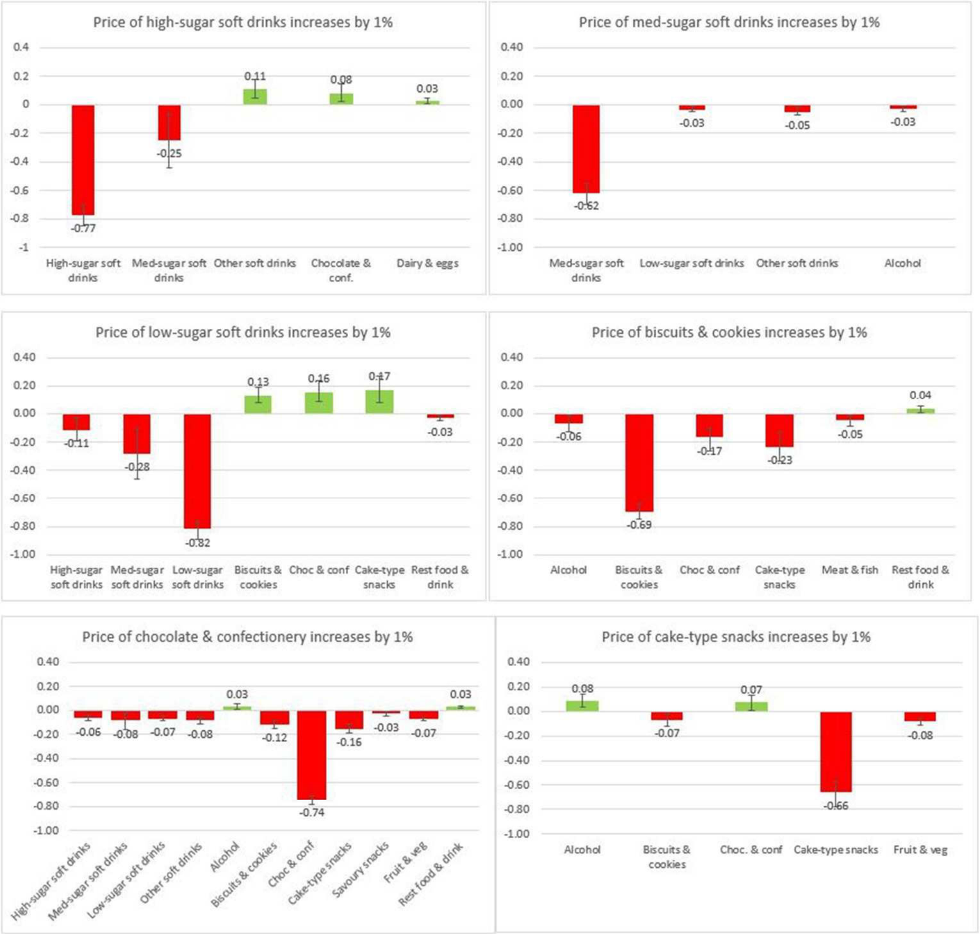

Notes: High-sugar soft drinks: >8g of sugar/100ml; medium-sugar soft drinks: 5-8g of sugar/100ml; low-sugar soft drinks: <5g of sugar/100ml; other soft drinks: water, fruit juice with no added sugars and milk-based drinks; 95% bias-corrected confidence intervals based on n=250 bootstrap replications.
Figures show elasticities for which the 95% CI excluded zero. For full set of elasticity, estimates see appendix 2.

Figure 1. Change in demand (%) as a response to 1% price increase in soft drinks and sweet snacks (all households n=623,459)

75x75mm (300 x 300 DPI)

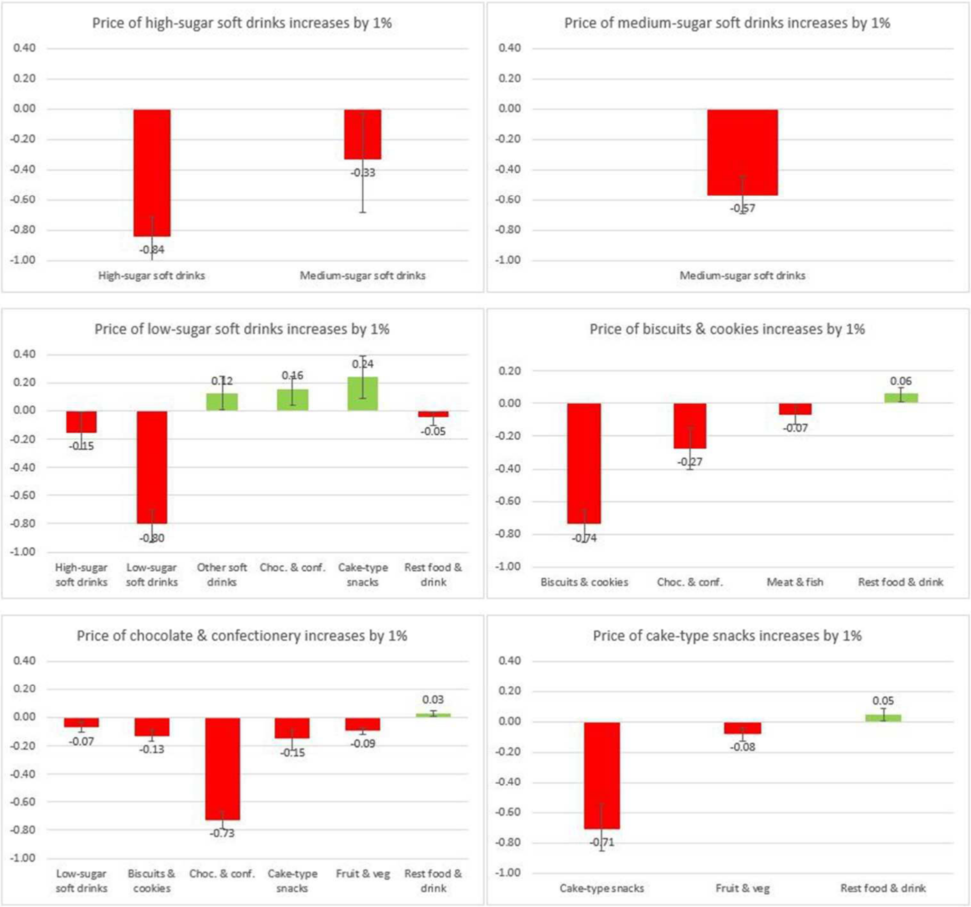

Notes: High-sugar soft drinks: >8g of sugar/100ml; medium-sugar soft drinks: 5-8g of sugar/100ml; low-sugar soft drinks: <5g of sugar/100ml; other soft drinks: water, fruit juice with no added sugars and milk-based drinks; 95% bias-corrected confidence intervals based on n=250 bootstrap replications.
Figures show elasticities for which the 95% CI excluded zero. For full set of elasticity, estimates see appendix 2.

Figure 2. Change in demand (%) as a response to 1% price increase in soft drinks and sweet snacks (low-income households n=223,174)

75x73mm (300 x 300 DPI)

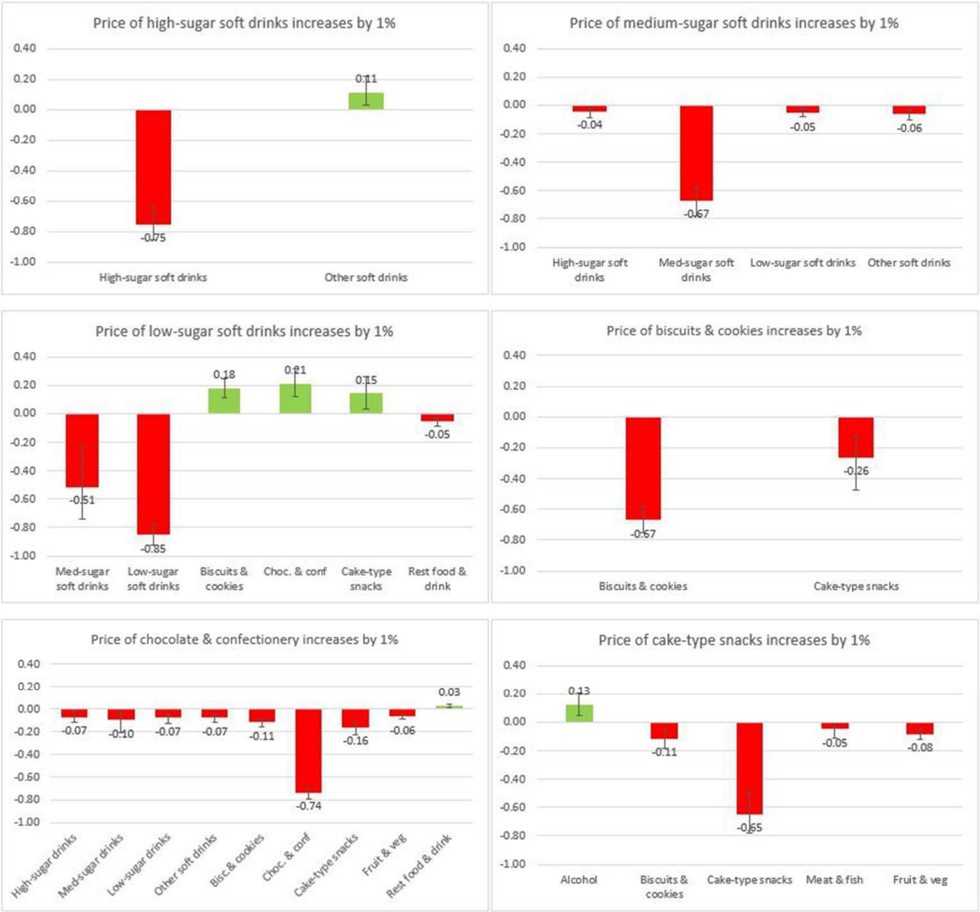

Notes: High-sugar soft drinks: >8g of sugar/100ml; medium-sugar soft drinks: 5-8g of sugar/100ml; low-sugar soft drinks: <5g of sugar/100ml; other soft drinks: water, fruit juice with no added sugars and milk-based drinks; 95% bias-corrected confidence intervals based on n=250 bootstrap replications.
Figures show elasticities for which the 95% CI excluded zero. For full set of elasticity, estimates see appendix 2.

Figure 3. Change in demand (%) as a response to 1% price increase in soft drinks and sweet snacks (mid-income households n=305,841)

74x73mm (300 x 300 DPI)

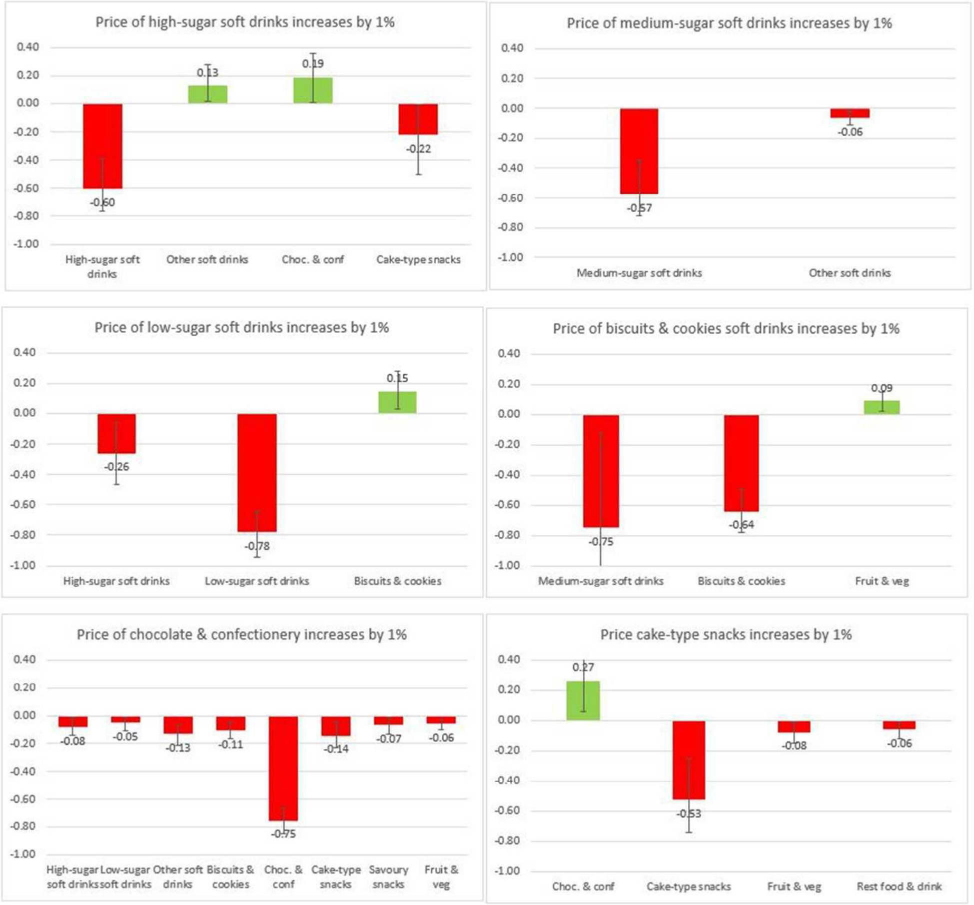

Notes: High-sugar soft drinks: >8g of sugar/100ml; medium-sugar soft drinks: 5-8g of sugar/100ml; low-sugar soft drinks: <5g of sugar/100ml; other soft drinks: water, fruit juice with no added sugars and milk-based drinks; 95% bias-corrected confidence intervals based on n=250 bootstrap replications.
Figures show elasticities for which the 95% CI excluded zero. For full set of elasticity, estimates see appendix 2.

Figure 4. Change in demand (%) as a response to 1% price increase in soft drinks and sweet snacks (high-income households n=94,444)

75x73mm (300 x 300 DPI)

Appendix 1: Demand modelling strategy

The demand model applied was based on the linear version of Almost Ideal Demand System where expenditure shares are modelled as a function of prices and total expenditure adjusted for all price levels:

$$w_{iht} = \alpha_i + \sum_{j=1}^{N} \gamma_{ij} lnp_{jht} + \beta_i \frac{lnx_{ht}}{P_{ht}} + \varepsilon_{iht} \quad (1)$$

Where:

w_{iht} is expenditure share of group *i* (i=1, 2, …, 13) for household *h* (h=1,2,…32,249) in 4-weekly periods *t* (t=1, 2, …, 26)

lnx_{ht} is the log of total household monthly expenditure on food and beverage per capita

lnp_{jht} is the log of price for category *j* for household *h* in period *t*

$P_{ht}$is a Laspeyres price index of geometrically weighted average prices defined as $lnP = \sum_i \overline{w}_i lnp_i$

α_i is a constant for group *i*

γ_{ij} and β_i are parameters to be estimated

ε_{iht} is a random disturbance

As not all households purchase items from each of the food and beverage groups in each period, the data includes zero-observations. These were more likely to occur in more disaggregated groups (e.g. 45% of observations among other soft drinks, 73% in cake-type snacks were zeroes). To deal with these zero observations that can bias the estimates, we followed a two-step procedure developed by Shonkweiler and Yen (1999).[1] In the first step, the decision to purchase beverages in any group was modelled as a function of lagged quantity of foods/beverages purchased in that group, household size, age of the main shopper, socio-economic group (A&B, C1&C2 or D&E), whether or not the household owns their house, income group (for the whole sample only), presence of children and time indicators to take into account seasonal trends, using a probit model. From the probit model, we estimated the probability density function (ϕ_i) and cumulative density function (Φ_i) of the predictions of the fitted model. These two variables were applied in the second step of estimating the demand function (1):

$$w_{iht}^{*} = \Phi_{iht}(w_{iht}) + \varphi_{iht}\phi_{iht} + \sum_{t=1}^{13} \rho_{it} T_{it} + v_{ih} + \varepsilon_{it} \quad (2)$$

Where:

T_{it} are indicator variables to capture any seasonal or other time effects (13 four-week periods)

v_{ih} is a fixed household effect

We estimated (2) equation-by-equation using a fixed effect model with robust clustered standard errors to allow for any misspecification, particularly serial correlation of observations within the households. Clusters were defined at the geographical area used in estimating prices (n=110).

The specification used (2) imposed the restrictions, compatible with the AIDS model, of adding-up $[\sum_{i=1}^{N} \alpha_i = 1; \sum_{i=1}^{N} \beta_i = 0]$ and homogeneity $[\sum_{i=1}^{N} \gamma_{ij} = 0]$.

For peer review only

There are two important sources of potential endogeneity in the model. First, total expenditure enters the model as a proxy for incomes while it is also used to calculate the expenditure shares. Furthermore, total expenditure might be endogenous because of possible correlation with unobserved characteristics affecting demand behaviour or because of shocks common to total expenditure and expenditure shares. Secondly, unit prices estimated from monthly aggregates of expenditure and volume are likely to be biased due to aggregation effects.[2] If prices or expenditures are correlated with the equation errors, estimators will be both biased and inconsistent.

To deal with quality effects in prices, we took the assumption that in a relatively small geographical area households face the same prices during the same time period. To estimate these geographical average unit values we calculated the monthly average prices for the (n=110) postcode areas which we observe in the data. Where the monthly price was missing (e.g. households did not purchase the products in this beverage group in a particular month), it was replaced by the first non-missing average of the previous and the following monthly prices.

To reduce possible endogeneity between expenditure shares (w_{iht}) and total expenditure (lnx_{ht}) that enters the demand equation in (1) we use the approach developed Blundell et al. (1999)[3] and regressed household per capita expenditure (lnx_{ht}) on household socio demographic characteristics (social class, income, income squared (whole sample only), household size and presence of children. The predicted values from the model were used as instruments for total expenditure (lnx_{ht}) in (1).

Uncompensated (Marshallian) elasticities were estimated for each beverage and food group, at sample averages (w and Φ) as follows:

$$e_{ij} = \Phi_i * (\frac{\gamma_{ij}}{w_i} - \frac{\beta_i w_j}{w_i}) - \Delta_{ij} \quad (3)$$

Where Δ_{ij} is the Kronecker delta which equals 1 when *i=j* and 0 otherwise.

Expenditure share equations in (2) are estimated with clustered (geographical area) robust standard errors and standard errors of the unconditional elasticities (3) are bootstrapped (250 replications) to account for possible bias arising from two-step procedure. Elasticities are reported with bias-corrected confidence intervals.

References

1. Shonkwiler JS, Yen ST. Two-Step Estimation of a Censored System of Equations. Am J Agr Econ. 1999 Nov 1;81(4):972–82.

2. Deaton A. Quality, quantity, and spatial variation of price. The Am Econ Rev. 1988;418–430.

3. Blundell R, Robin JM. Estimation in large and disaggregated demand systems: An estimator for conditionally linear systems. J Appl Econom. 1999;14(3):209-32.

For peer review only

Appendix 2: Price elasticities

Table 1. Price elasticities of demand in full sample (n=623,459)

	High-sugar soft drinks	Medium-sugar soft drinks	Low-sugar soft drinks	Other soft drinks	Alcohol	Biscuits & cookies	Chocolate & conf.	Cake-type snacks	Savoury snacks	Meat & fish	Dairy & eggs	Fruit & vegetables	Rest food & drink
High-sugar soft drinks	**-0.77<br>[-0.85;-0.70]**	-0.03<br>[-0.06;0]	**-0.11<br>[-0.19;-0.02]**	-0.05<br>[-0.11;0.02]	**-0.19<br>[-0.24;-0.14]**	0.02<br>[-0.07;0.10]	**-0.06<br>[-0.09;-0.04]**	-0.02<br>[-0.11;0.06]	**0.22<br>[0.11;0.35]**	**-0.29<br>[-0.41;-0.15]**	**0.26<br>[0.18;0.34]**	-0.08<br>[-0.17;0.01]	0<br>[-0.17;0.17]
Medium-sugar soft drinks	**-0.25<br>[-0.44;-0.06]**	**-0.62<br>[-0.70;-0.55]**	**-0.28<br>[-0.46;-0.10]**	**-0.33<br>[-0.53;-0.16]**	**-0.20<br>[-0.33;-0.06]**	-0.19<br>[-0.44;0.03]	**-0.08<br>[-0.16;-0.02]**	-0.06<br>[-0.28;0.14]	**-0.34<br>[-0.63;-0.07]**	**1.10<br>[0.76;1.38]**	**-0.22<br>[-0.43;-0.01]**	**0.37<br>[0.13;0.60]**	-0.08<br>[-0.51;0.41]
Low-sugar soft drinks	-0.01<br>[-0.06;0.05]	**-0.03<br>[-0.05;-0.01]**	**-0.82<br>[-0.89;-0.76]**	**-0.13<br>[-0.17;-0.09]**	**-0.25<br>[-0.29;-0.21]**	-0.02<br>[-0.08;0.05]	**-0.07<br>[-0.09;-0.05]**	0<br>[-0.05;0.06]	-0.01<br>[-0.10;0.07]	0.05<br>[-0.04;0.14]	**0.15<br>[0.09;0.21]**	**0.12<br>[0.07;0.19]**	-0.04<br>[-0.18;0.08]
Other soft drinks	**0.11<br>[0.05;0.18]**	**-0.05<br>[-0.07;-0.02]**	0<br>[-0.08;0.05]	**-0.83<br>[-0.88;-0.77]**	**-0.17<br>[-0.21;-0.12]**	-0.02<br>[-0.10;0.05]	**-0.08<br>[-0.11;-0.06]**	0.01<br>[-0.05;0.08]	0.06<br>[-0.04;0.17]	**-0.17<br>[-0.27;-0.07]**	**0.13<br>[0.06;0.21]**	0.03<br>[-0.04;0.10]	0.01<br>[-0.14;0.14]
Alcohol	-0.04<br>[-0.10;0.00]	**-0.03<br>[-0.05;-0.01]**	-0.02<br>[-0.08;0.03]	**-0.10<br>[-0.14;-0.05]**	**-0.90<br>[-0.94;-0.86]**	**-0.06<br>[-0.13;-0.01]**	**0.03<br>[0.01;0.05]**	**0.08<br>[0.03;0.14]**	**0.11<br>[0.04;0.19]**	0.08<br>[-0.01;0.17]	0.02<br>[-0.03;0.08]	**-0.07<br>[-0.14;-0.01]**	**-0.37<br>[-0.47;-0.25]**
Biscuits & cookies	0<br>[-0.06;0.04]	-0.01<br>[-0.02;0.02]	**0.13<br>[0.08;0.19]**	**0.05<br>[0.01;0.09]**	**0.06<br>[0.02;0.10]**	**-0.69<br>[-0.75;-0.64]**	**-0.12<br>[-0.15;-0.09]**	**-0.07<br>[-0.12;-0.02]**	**0.13<br>[0.03;0.20]**	**-0.18<br>[-0.28;-0.10]**	-0.04<br>[-0.11;0.01]	0<br>[-0.06;0.07]	**-0.40<br>[-0.51;-0.27]**
Chocolate & conf.	**0.08<br>[0.02;0.15]**	0.02<br>[-0.01;0.04]	**0.16<br>[0.09;0.24]**	0.01<br>[-0.04;0.07]	**0.07<br>[0.01;0.12]**	**-0.17<br>[-0.26;-0.10]**	**-0.74<br>[-0.78;-0.71]**	**0.07<br>[0.01;0.13]**	**0.27<br>[0.17;0.39]**	**0.44<br>[0.30;0.54]**	**-0.30<br>[-0.38;-0.22]**	**-0.26<br>[-0.35;-0.19]**	**-0.94<br>[-1.10;-0.77]**
Cake-type snacks	-0.02<br>[-0.11;0.06]	0<br>[-0.03;0.03]	**0.17<br>[0.08;0.27]**	0.09<br>[0;0.16]	-0.02<br>[-0.09;0.05]	**-0.23<br>[-0.33;-0.13]**	**-0.16<br>[-0.19;-0.11]**	**-0.66<br>[-0.78;-0.57]**	**0.31<br>[0.14;0.44]**	**-0.32<br>[-0.45;-0.14]**	0.06<br>[-0.04;0.17]	-0.08<br>[-0.17;0.02]	-0.08<br>[-0.17;0.02]
Savoury snacks	0<br>[-0.05;0.04]	0.01<br>[0;0.03]	0<br>[-0.05;0.05]	-0.04<br>[-0.07;0]	-0.03<br>[-0.06;0]	-0.02<br>[-0.07;0.04]	**-0.03<br>[-0.05;-0.01]**	-0.04<br>[-0.10;0.01]	**-0.75<br>[-0.82;-0.67]**	-0.03<br>[-0.12;0.05]	0<br>[-0.05;0.05]	0<br>[-0.05;0.05]	**-0.23<br>[-0.32;-0.11]**
Meat & fish	0<br>[-0.03;0.03]	0<br>[-0.01;0.01]	0.01<br>[-0.03;0.04]	0.01<br>[-0.01;0.04]	0.03<br>[0;0.05]	**-0.05<br>[-0.09;-0.01]**	0<br>[-0.01;0.02]	-0.02<br>[-0.05;0]	-0.04<br>[-0.10;0.01]	**-0.76<br>[-0.81;-0.70]**	**-0.12<br>[-0.15;-0.08]**	**-0.08<br>[-0.12;-0.04]**	-0.02<br>[-0.08;0.06]
Dairy & eggs	**0.03<br>[0.01;0.05]**	-0.01<br>[-0.02;0]	0.01<br>[-0.01;0.03]	**0.04<br>[0.02;0.06]**	0.01<br>[-0.01;0.02]	0.02<br>[0;0.05]	-0.01<br>[-0.03;0]	-0.01<br>[-0.04;0.01]	-0.02<br>[-0.06;0.02]	-0.03<br>[-0.06;0.02]	**-0.88<br>[-0.91;-0.85]**	**-0.12<br>[-0.14;-0.09]**	**-0.09<br>[-0.14;-0.03]**
Fruit & veg	-0.01<br>[-0.03;0.02]	0.00<br>[-0.01;0.01]	0<br>[-0.03;0.02]	-0.01<br>[-0.03;0]	-0.02<br>[-0.04;0.01]	0.03<br>[0;0.06]	**-0.07<br>[-0.09;-0.06]**	**-0.08<br>[-0.11;-0.06]**	**0.07<br>[0.03;0.12]**	**-0.10<br>[-0.14;-0.05]**	-0.01<br>[-0.04;0.02]	**-0.60<br>[-0.63;-0.57]**	-0.06<br>[-0.13;0.01]
Rest food & drink	-0.01<br>[-0.04;0.01]	0<br>[-0.01;0.01]	**-0.03<br>[-0.05;-0.01]**	**0.04<br>[0.02;0.06]**	0<br>[-0.01;0.02]	**0.04<br>[0.01;0.06]**	**0.03<br>[0.02;0.04]**	0<br>[-0.02;0.02]	**-0.13<br>[-0.16;-0.03]**	**-0.1<br>[-0.14;-0.06]**	-0.01<br>[-0.04;0.02]	-0.03<br>[-0.06;0]	**-0.66<br>[-0.71;-0.61]**

Notes: Elasticities in bold indicate those where 95% confidence intervals do not include zero. Columns indicate the group of price change and rows indicate the group of demand change. High-sugar soft drinks: >8g of sugar/100ml; medium-sugar soft drinks: 5-8g of sugar/100ml; low-sugar soft drinks: <5g of sugar/100ml; other soft drinks: water, fruit juice with no added sugars and milk-based drinks.

For peer review only

Table 2. Price elasticities of demand in low-income (annual household income £<20,000) sample (n=223,174)

	High-sugar soft drinks	Medium-sugar soft drinks	Low-sugar soft drinks	Other soft drinks	Alcohol	Biscuits & cookies	Chocolate & conf.	Cake-type snacks	Savoury snacks	Meat & fish	Dairy & eggs	Fruit & vegetables	Rest food & drink
High-sugar soft drinks	**-0.84 [-1.00;-0.71]**	-0.03 [-0.09;0.02]	**-0.15 [-0.27;-0.01]**	0.00 [-0.11;0.12]	**-0.25 [-0.36;-0.16]**	0.02 [-0.10;0.19]	-0.06 [-0.11;0]	-0.07 [-0.24;0.11]	0.17 [0;0.38]	-0.02 [-0.28;0.21]	**0.30 [0.15;0.44]**	-0.07 [-0.24;0.10]	-0.08 [-0.43;0.23]
Medium-sugar soft drinks	**-0.33 [-0.68;-0.02]**	**-0.57 [-0.69;-0.44]**	-0.05 [-0.38;0.26]	**-0.31 [-0.62;-0.07]**	-0.25 [-0.46;0]	-0.08 [-0.49;0.29]	-0.10 [-0.23;0.01]	-0.17 [-0.64;0.22]	-0.39 [-0.89;0.31]	**0.98 [0.39;1.46]**	**-0.39 [-0.71;-0.03]**	**0.43 [0.02;0.83]**	-0.06 [-0.87;0.73]
Low-sugar soft drinks	0 [-0.10;0.09]	-0.02 [-0.05;0.02]	**-0.80 [-0.93;-0.70]**	**-0.13 [-0.22;-0.05]**	**-0.28 [-0.35;-0.21]**	-0.01 [-0.13;0.10]	**-0.07 [-0.10;-0.03]**	-0.03 [-0.15;0.06]	0.05 [-0.18;0.17]	0.03 [-0.16;0.21]	**0.14 [0.03;0.25]**	**0.21 [0.10;0.33]**	-0.14 [-0.44;0.06]
Other soft drinks	0.08 [-0.04;0.23]	-0.01 [-0.05;0.05]	**0.12 [0.01;0.25]**	**-0.89 [-0.98;-0.80]**	**-0.27 [-0.37;-0.17]**	-0.08 [-0.22;0.08]	-0.06 [-0.11;-0.02]	0 [-0.12;0.13]	0.09 [-0.08;0.27]	**-0.27 [-0.51;-0.07]**	**0.19 [0.05;0.32]**	**0.26 [0.11;0.38]**	-0.17 [-0.41;0.06]
Alcohol	-0.01 [-0.12;0.09]	-0.04 [-0.08;0]	0.01 [-0.06;0.11]	-0.05 [-0.14;0.02]	**-0.92 [-0.98;-0.85]**	-0.04 [-0.15;0.06]	0.02 [-0.01;0.06]	-0.04 [-0.13;0.06]	**0.17 [0.05;0.33]**	0.15 [-0.02;0.30]	0.12 [-0.01;0.21]	-0.08 [-0.17;0.06]	**-0.50 [-0.69;-0.28]**
Biscuits & cookies	0.01 [-0.09;0.10]	-0.03 [-0.06;0]	0.07 [0;0.16]	0.04 [-0.03;0.11]	0 [-0.06;0.06]	**-0.74 [-0.85;-0.65]**	**-0.13 [-0.17;-0.09]**	-0.01 [-0.11;0.10]	0.12 [-0.02;0.26]	-0.16 [-0.30;0]	-0.03 [-0.12;0.07]	0.04 [-0.07;0.14]	**-0.34 [-0.60;-0.18]**
Chocolate & conf.	0.08 [-0.03;0.19]	0.01 [-0.03;0.04]	**0.16 [0.04;0.25]**	0.02 [-0.06;0.10]	0.07 [0;0.16]	**-0.27 [-0.40;-0.15]**	**-0.73 [-0.79;-0.67]**	0.02 [-0.09;0.15]	**0.19 [0.02;0.35]**	**0.49 [0.31;0.67]**	**-0.29 [-0.42;-0.16]**	**-0.35 [-0.46;-0.21]**	**-0.74 [-1.00;-0.48]**
Cake-type snacks	-0.12 [-0.27;0.04]	-0.04 [-0.10;0.02]	**0.24 [0.09;0.39]**	**0.14 [0.03;0.27]**	-0.09 [-0.20;0.01]	-0.19 [-0.35;0.02]	**-0.15 [-0.23;-0.09]**	**-0.71 [-0.85;-0.54]**	**0.27 [0.06;0.47]**	**-0.38 [-0.68;-0.11]**	0.10 [-0.05;0.29]	-0.07 [-0.23;0.11]	**-0.44 [-0.83;-0.12]**
Savoury snacks	0.02 [-0.07;0.09]	0.02 [-0.01;0.05]	-0.02 [-0.10;0.06]	0.02 [-0.05;0.08]	-0.03 [-0.10;0.02]	-0.02 [-0.11;0.07]	-0.03 [-0.08;0]	-0.07 [-0.16;0]	**-0.71 [-0.83;-0.59]**	0 [-0.14;0.12]	-0.02 [-0.10;0.07]	-0.02 [-0.10;0.07]	**-0.27 [-0.48;-0.09]**
Meat & fish	0.02 [-0.04;0.07]	0 [-0.02;0.02]	-0.01 [-0.06;0.04]	0.03 [-0.02;0.07]	**0.05 [0.01;0.09]**	**-0.07 [-0.13;-0.01]**	0.02 [0;0.05]	-0.01 [-0.06;0.06]	0.01 [-0.06;0.10]	**-0.80 [-0.90;-0.70]**	**-0.15 [-0.21;-0.08]**	**-0.11 [-0.17;-0.04]**	-0.10 [-0.22;0.02]
Dairy & eggs	0.02 [-0.01;0.06]	-0.01 [-0.02;0.01]	-0.01 [-0.05;0.03]	0.03 [0;0.07]	0.02 [-0.01;0.05]	0.02 [-0.02;0.07]	-0.02 [-0.04;0.01]	0.01 [-0.03;0.06]	-0.05 [-0.10;0.02]	-0.07 [-0.13;0]	**-0.86 [-0.90;-0.81]**	**-0.12 [-0.17;-0.08]**	-0.05 [-0.15;0.05]
Fruit & veg	-0.03 [-0.08;0.01]	0 [-0.02;0.02]	0.02 [-0.02;0.07]	-0.03 [-0.06;0.01]	-0.02 [-0.06;0.02]	0.01 [-0.04;0.07]	**-0.09 [-0.12;-0.07]**	**-0.08 [-0.13;-0.04]**	**0.13 [0.05;0.20]**	-0.08 [-0.16;0]	-0.03 [-0.09;0.02]	**-0.58 [-0.64;-0.53]**	**-0.15 [-0.26;-0.04]**
Rest food & drink	-0.01 [-0.04;0.04]	0.01 [-0.01;0.02]	**-0.05 [-0.1;-0.01]**	0.01 [-0.02;0.05]	0.02 [0;0.05]	**0.06 [0.01;0.10]**	**0.03 [0.01;0.05]**	**0.05 [0.01;0.09]**	-0.18 [-0.24;0]	**-0.12 [-0.18;-0.06]**	-0.04 [-0.08;0.01]	-0.03 [-0.08;0.01]	**-0.57 [-0.66;-0.48]**

Notes: Elasticities in bold indicate those where 95% confidence intervals do not include zero. Columns indicate the group of price change and rows indicate the group of demand change. High-sugar soft drinks: >8g of sugar/100ml; medium-sugar soft drinks: 5-8g of sugar/100ml; low-sugar soft drinks: <5g of sugar/100ml; other soft drinks: water, fruit juice with no added sugars and milk-based drinks.

Table 3. Price elasticities of demand in mid-income (annual household income £20,000-£49,000) sample (n=305,841)

	High-sugar soft drinks	Medium-sugar soft drinks	Low-sugar soft drinks	Other soft drinks	Alcohol	Biscuits & cookies	Chocolate & conf.	Cake-type snacks	Savoury snacks	Meat & fish	Dairy & eggs	Fruit & vegetables	Rest food & drink
High-sugar soft drinks	**-0.75 [-0.85;-0.64]**	**-0.04 [-0.09;-0.01]**	-0.06 [-0.16;0.04]	-0.07 [-0.17;0.02]	**-0.15 [-0.22;-0.06]**	0.05 [-0.07;0.18]	**-0.07 [-0.11;-0.02]**	-0.02 [-0.14;0.11]	**0.27 [0.10;0.42]**	**-0.36 [-0.53;-0.17]**	**0.20 [0.08;0.33]**	-0.10 [-0.22;0.03]	0.01 [-0.27;0.22]
Medium-sugar soft drinks	-0.20 [-0.43;0.05]	**-0.67 [-0.78;-0.57]**	**-0.51 [-0.74;-0.23]**	**-0.36 [-0.60;-0.15]**	-0.12 [-0.30;0.13]	-0.06 [-0.40;0.27]	**-0.10 [-0.20;-0.01]**	0.00 [-0.27;0.27]	**-0.48 [-0.94;-0.11]**	**1.24 [0.72;1.69]**	-0.19 [-0.44;0.08]	**0.41 [0.10;0.76]**	-0.06 [-0.77;0.55]
Low-sugar soft drinks	-0.01 [-0.08;0.06]	**-0.05 [-0.08;-0.02]**	**-0.85 [-0.92;-0.77]**	**-0.09 [-0.14;-0.02]**	**-0.23 [-0.28;-0.18]**	-0.01 [-0.09;0.07]	**-0.07 [-0.12;-0.04]**	0.00 [-0.08;0.10]	-0.07 [-0.20;0.04]	**0.13 [0.01;0.25]**	**0.16 [0.08;0.26]**	0.07 [-0.02;0.15]	-0.01 [-0.14;0.17]
Other soft drinks	**0.11 [0.03;0.22]**	**-0.06 [-0.10;-0.03]**	0.02 [-0.07;0.12]	**-0.79 [-0.87;-0.73]**	**-0.13 [-0.19;-0.07]**	0.03 [-0.08;0.14]	**-0.07 [-0.11;-0.04]**	-0.01 [-0.11;0.09]	0.03 [-0.10;0.18]	-0.13 [-0.25;0.06]	0.13 [0;0.22]	-0.04 [-0.14;0.06]	-0.02 [-0.23;0.20]
Alcohol	-0.04 [-0.12;0.03]	-0.02 [-0.05;0]	0.01 [-0.05;0.09]	**-0.13 [-0.20;-0.08]**	**-0.91 [-0.95;-0.85]**	-0.05 [-0.15;0.03]	0.03 [0;0.05]	**0.13 [0.05;0.21]**	**0.13 [0.01;0.23]**	0.04 [-0.08;0.17]	-0.04 [-0.11;0.04]	**-0.10 [-0.18;-0.02]**	**-0.29 [-0.46;-0.14]**
Biscuits & cookies	-0.01 [-0.08;0.07]	0 [-0.02;0.03]	**0.18 [0.11;0.25]**	0.05 [0;0.11]	**0.09 [0.02;0.13]**	**-0.67 [-0.75;-0.58]**	**-0.11 [-0.15;-0.08]**	**-0.11 [-0.18;-0.05]**	**0.14 [0.03;0.24]**	**-0.19 [-0.33;-0.08]**	-0.06 [-0.16;0.01]	-0.01 [-0.09;0.08]	**-0.45 [-0.62;-0.29]**
Chocolate & conf.	0.06 [-0.05;0.17]	0.01 [-0.02;0.04]	**0.21 [0.12;0.32]**	0.02 [-0.07;0.09]	0.07 [0;0.14]	-0.07 [-0.18;0.03]	**-0.74 [-0.79;-0.69]**	0.07 [-0.02;0.19]	**0.27 [0.12;0.42]**	**0.41 [0.24;0.58]**	**-0.30 [-0.42;-0.19]**	**-0.20 [-0.31;-0.09]**	**-1.03 [-1.32;-0.81]**
Cake-type snacks	0.12 [-0.01;0.26]	0.05 [0;0.09]	**0.15 [0.03;0.26]**	0.06 [-0.05;0.17]	0.01 [-0.09;0.09]	**-0.26 [-0.47;-0.13]**	**-0.16 [-0.22;-0.11]**	**-0.65 [-0.78;-0.49]**	**0.25 [0.08;0.45]**	**-0.24 [-0.42;-0.04]**	-0.02 [-0.17;0.11]	**-0.16 [-0.31;-0.01]**	**-0.55 [-0.82;-0.23]**
Savoury snacks	-0.02 [-0.08;0.03]	0.01 [-0.01;0.04]	0.03 [-0.04;0.08]	**-0.06 [-0.11;-0.01]**	-0.02 [-0.08;0.02]	-0.01 [-0.10;0.05]	-0.02 [-0.05;0.02]	-0.02 [-0.11;0.04]	**-0.75 [-0.84;-0.65]**	-0.05 [-0.16;0.05]	-0.01 [-0.09;0.07]	-0.01 [-0.09;0.07]	**-0.23 [-0.35;-0.10]**
Meat & fish	-0.01 [-0.05;0.03]	0.01 [0;0.03]	0.03 [-0.02;0.07]	0.01 [-0.03;0.04]	0.03 [0;0.06]	-0.05 [-0.10;0]	0 [-0.02;0.02]	**-0.05 [-0.11;-0.01]**	-0.06 [-0.14;0]	**-0.75 [-0.81;-0.67]**	**-0.12 [-0.17;-0.07]**	**-0.08 [-0.13;-0.02]**	0.04 [-0.05;0.14]
Dairy & eggs	0.03 [-0.01;0.06]	-0.01 [-0.02;0]	0.03 [-0.01;0.07]	**0.04 [0.01;0.06]**	0 [-0.02;0.03]	0.03 [-0.01;0.07]	-0.01 [-0.03;0.01]	-0.03 [-0.07;0.01]	-0.02 [-0.09;0.03]	-0.01 [-0.07;0.04]	**-0.89 [-0.94;-0.84]**	**-0.13 [-0.16;-0.08]**	**-0.09 [-0.19;-0.01]**
Fruit & veg	0.01 [-0.02;0.05]	0.01 [0;0.02]	-0.02 [-0.05;0.02]	-0.03 [-0.05;0]	0 [-0.03;0.02]	0.02 [-0.02;0.06]	**-0.06 [-0.08;-0.04]**	**-0.08 [-0.12;-0.05]**	0.05 [0;0.11]	**-0.12 [-0.18;-0.05]**	0.02 [-0.02;0.06]	**-0.58 [-0.62;-0.54]**	-0.04 [-0.12;0.04]
Rest food & drink	0 [-0.05;0.05]	0 [-0.01;0.02]	**-0.05 [-0.09;-0.03]**	**0.05 [0.03;0.08]**	-0.02 [-0.04;0.01]	0.01 [-0.03;0.05]	**0.03 [0.01;0.04]**	0 [-0.04;0.03]	**-0.11 [-0.15;-0.03]**	**-0.09 [-0.14;-0.04]**	0.02 [-0.01;0.06]	-0.02 [-0.05;0.01]	**-0.7 [-0.76; -0.63]**

Notes: Elasticities in bold indicate those where 95% confidence intervals do not include zero. Columns indicate the group of price change and rows indicate the group of demand change. High-sugar soft drinks: >8g of sugar/100ml; medium-sugar soft drinks: 5-8g of sugar/100ml; low-sugar soft drinks: <5g of sugar/100ml; other soft drinks: water, fruit juice with no added sugars and milk-based drinks.

For peer review only

Table 4. Price elasticities of demand in high-income (annual household income > £50,000) sample (n=94,444)

	High-sugar soft drinks	Medium-sugar soft drinks	Low-sugar soft drinks	Other soft drinks	Alcohol	Biscuits & cookies	Chocolate & conf.	Cake-type snacks	Savoury snacks	Meat & fish	Dairy & eggs	Fruit & veg	Rest food & drink
High-sugar soft drinks	**-0.60<br>[-0.76;-0.39]**	-0.02<br>[-0.10;0.05]	**-0.26<br>[-0.46;-0.06]**	-0.03<br>[-0.19;0.12]	**-0.18<br>[-0.30;-0.04]**	0.03<br>[-0.18;0.33]	**-0.08<br>[-0.14;-0.01]**	0.06<br>[-0.15;0.27]	**0.32<br>[0.03;0.58]**	**-0.73<br>[-1.05;-0.34]**	**0.31<br>[0.14;0.55]**	-0.05<br>[-0.27;0.16]	0.19<br>[-0.34;0.60]
Medium-sugar soft drinks	-0.19<br>[-0.63;0.40]	**-0.57<br>[-0.72;-0.34]**	0.01<br>[-0.42;0.46]	**-0.39<br>[-0.76;-0.02]**	-0.26<br>[-0.66;0.03]	**-0.75<br>[-1.23;-0.13]**	0.01<br>[-0.15;0.17]	-0.16<br>[-0.65;0.34]	-0.08<br>[-0.75;0.59]	**0.90<br>[0.24;1.70]**	0.29<br>[-0.14;0.88]	0.12<br>[-0.43;0.72]	0.09<br>[-1.19;0.91]
Low-sugar soft drinks	-0.01<br>[-0.13;0.11]	-0.01<br>[-0.07;0.03]	**-0.78<br>[-0.94;-0.64]**	**-0.23<br>[-0.33;-0.11]**	**-0.22<br>[-0.29;-0.11]**	-0.06<br>[-0.21;0.07]	**-0.05<br>[-0.11;-0.01]**	0.07<br>[-0.07;0.22]	0.05<br>[-0.15;0.23]	-0.15<br>[-0.43;0.07]	0.13<br>[-0.02;0.27]	**0.16<br>[0.02;0.30]**	0.14<br>[-0.15;0.41]
Other soft drinks	**0.13<br>[0.02;0.28]**	**-0.06<br>[-0.11;-0.02]**	-0.13<br>[-0.28;0.02]	**-0.83<br>[-0.96;-0.71]**	-0.09<br>[-0.20;0.01]	-0.02<br>[-0.17;0.15]	**-0.13<br>[-0.21;-0.07]**	0.09<br>[-0.05;0.25]	0.12<br>[-0.08;0.36]	-0.19<br>[-0.45;0.08]	0.01<br>[-0.14;0.18]	-0.11<br>[-0.27;0.11]	0.29<br>[-0.04;0.58]
Alcohol	-0.10<br>[-0.21;0.05]	-0.04<br>[-0.09;0]	-0.07<br>[-0.21;0.07]	-0.08<br>[-0.19;0.01]	**-0.82<br>[-0.93;-0.74]**	-0.12<br>[-0.29;0.03]	0.04<br>[0;0.09]	0.11<br>[-0.05;0.25]	0.01<br>[-0.18;0.22]	0.11<br>[-0.10;0.30]	0.00<br>[-0.15;0.15]	-0.01<br>[-0.17;0.12]	-0.33<br>[-0.61;0]
Biscuits & cookies	0<br>[-0.14;0.12]	0.02<br>[-0.02;0.06]	**0.15<br>[0.03;0.28]**	0.06<br>[-0.05;0.17]	0.06<br>[-0.03;0.15]	**-0.64<br>[-0.78;-0.50]**	**-0.11<br>[-0.16;-0.04]**	-0.07<br>[-0.23;0.07]	0.13<br>[-0.09;0.31]	**-0.19<br>[-0.43;-0.01]**	0.02<br>[-0.10;0.20]	-0.12<br>[-0.26;0.08]	-0.35<br>[-0.62;0.01]
Chocolate & conf.	**0.19<br>[0.01;0.36]**	0.05<br>[-0.02;0.13]	-0.01<br>[-0.19;0.23]	-0.01<br>[-0.15;0.15]	0.13<br>[0;0.27]	-0.18<br>[-0.39;0.01]	**-0.75<br>[-0.85;-0.66]**	**0.27<br>[0.06;0.42]**	**0.49<br>[0.24;0.72]**	**0.41<br>[0.11;0.73]**	**-0.30<br>[-0.48;-0.08]**	**-0.22<br>[-0.44;-0.03]**	**-1.10<br>[-1.44;-0.72]**
Cake-type snacks	**-0.22<br>[-0.50;-0.01]**	-0.08<br>[-0.16;0.02]	0.12<br>[-0.08;0.37]	-0.02<br>[-0.19;0.18]	0.12<br>[-0.05;0.30]	-0.25<br>[-0.52;0.02]	**-0.14<br>[-0.23;-0.04]**	**-0.53<br>[-0.74;-0.25]**	**0.70<br>[0.30;1.03]**	-0.24<br>[-0.63;0.20]	0.25<br>[-0.06;0.51]	0.04<br>[-0.23;0.32]	**-1.18<br>[-1.71;-0.60]**
Savoury snacks	0.03<br>[-0.07;0.16]	0.01<br>[-0.04;0.04]	-0.03<br>[-0.15;0.06]	-0.07<br>[-0.15;0.04]	-0.03<br>[-0.11;0.05]	-0.04<br>[-0.17;0.09]	**-0.07<br>[-0.13;-0.01]**	-0.05<br>[-0.15;0.07]	**-0.83<br>[-0.98;-0.68]**	-0.05<br>[-0.25;0.15]	0.08<br>[-0.06;0.21]	0.07<br>[-0.06;0.21]	-0.14<br>[-0.36;0.11]
Meat & fish	-0.01<br>[-0.10;0.06]	0<br>[-0.03;0.02]	0.01<br>[-0.07;0.09]	0<br>[-0.07;0.05]	-0.01<br>[-0.07;0.04]	0<br>[-0.07;0.08]	-0.01<br>[-0.04;0.03]	0.01<br>[-0.08;0.07]	-0.11<br>[-0.24;0.01]	**-0.70<br>[-0.81;-0.57]**	-0.06<br>[-0.14;0.03]	-0.04<br>[-0.12;0.05]	-0.02<br>[-0.17;0.14]
Dairy & eggs	0.02<br>[-0.05;0.07]	0<br>[-0.02;0.02]	0.01<br>[-0.04;0.06]	0.04<br>[-0.01;0.10]	-0.02<br>[-0.06;0.03]	-0.01<br>[-0.07;0.06]	-0.03<br>[-0.06;0]	0<br>[-0.05;0.08]	-0.02<br>[-0.12;0.08]	0.02<br>[-0.06;0.12]	**-0.93<br>[-1.01;-0.86]**	**-0.09<br>[-0.17;-0.03]**	**-0.23<br>[-0.39;-0.06]**
Fruit & veg	0<br>[-0.06;0.07]	-0.02<br>[-0.04;0]	0.01<br>[-0.06;0.07]	0.04<br>[-0.01;0.09]	-0.03<br>[-0.07;0.01]	**0.09<br>[0.02;0.15]**	**-0.06<br>[-0.10;-0.03]**	**-0.08<br>[-0.15;-0.01]**	0.03<br>[-0.06;0.12]	-0.08<br>[-0.19;0.03]	-0.03<br>[-0.11;0.06]	**-0.69<br>[-0.75;-0.58]**	0.07<br>[-0.12;0.21]
Rest food & drink	-0.02<br>[-0.05;0.09]	0.01<br>[-0.01;0.03]	0.01<br>[-0.04;0.07]	0.03<br>[-0.01;0.07]	-0.01<br>[-0.05;0.02]	0.05<br>[-0.02;0.11]	0.04<br>[0;0.06]	**-0.06<br>[-0.12;-0.01]**	**-0.1<br>[-0.18;-0.01]**	**-0.1<br>[-0.19;-0.01]**	-0.02<br>[-0.08;0.05]	-0.06<br>[-0.13;0]	**-0.7<br>[-0.83;-0.58]**

Notes: Elasticities in bold indicate those where 95% confidence intervals do not include zero. Columns indicate the group of price change and rows indicate the group of demand change. High-sugar soft drinks: >8g of sugar/100ml; medium-sugar soft drinks: 5-8g of sugar/100ml; low-sugar soft drinks: <5g of sugar/100ml; other soft drinks: water, fruit juice with no added sugars and milk-based drinks.

For peer review only

STROBE Checklist statement

Smith et al. "Reducing sugar consumption: are sweet snacks more sensitive to price increases than sugar-sweetened beverages?"

	Item No	Recommendation
**Title and abstract**	1	(*a*) Indicate the study's design with a commonly used term in the title or the abstract [abstract]
		(*b*) Provide in the abstract an informative and balanced summary of what was done and what was found [included]
**Introduction**		
Background/rationale	2	Explain the scientific background and rationale for the investigation being reported [reported on page 4]
Objectives	3	State specific objectives, including any prespecified hypotheses [reported on page 4]
**Methods**		
Study design	4	Present key elements of study design early in the paper [page 4]
Setting	5	Describe the setting, locations, and relevant dates, including periods of recruitment, exposure, follow-up, and data collection [page 4/5 where relevant; secondary data]
Participants	6	(*a*) Give the eligibility criteria, and the sources and methods of selection of participants
Variables	7	Clearly define all outcomes, exposures, predictors, potential confounders, and effect modifiers. Give diagnostic criteria, if applicable [demand model briefly explained in page 5, and explained in detail in technical appendix]
Data sources/ measurement	8*	For each variable of interest, give sources of data and details of methods of assessment (measurement). Describe comparability of assessment methods if there is more than one group [explained in text on page 5 or in/adjacent tables]
Bias	9	Describe any efforts to address potential sources of bias [described in brief in page 5, in technical appendix in detail]
Study size	10	Explain how the study size was arrived at [explained in page 4]
Quantitative variables	11	Explain how quantitative variables were handled in the analyses. If applicable, describe which groupings were chosen and why [explained in pages 4-5]
Statistical methods	12	(*a*) Describe all statistical methods, including those used to control for confounding [technical appendix]
		(*b*) Describe any methods used to examine subgroups and interactions [page 5]
		(*c*) Explain how missing data were addressed [page 4]
		(*d*) If applicable, describe analytical methods taking account of sampling strategy [n/a]
		(*e*) Describe any sensitivity analyses [n/a]
**Results**		
Participants	13*	(a) Report numbers of individuals at each stage of study—eg numbers potentially eligible, examined for eligibility, confirmed eligible, included in the study, completing follow-up, and analysed [n/a as secondary data]
		(b) Give reasons for non-participation at each stage [n/a]
		(c) Consider use of a flow diagram [n/a]
Descriptive data	14*	(a) Give characteristics of study participants (eg demographic, clinical, social) and information on exposures and potential confounders [table 1]

For peer review only

For peer review only - http://bmjopen.bmj.com/site/about/guidelines.xhtml

		(b) Indicate number of participants with missing data for each variable of interest [page 4 where relevant]
Outcome data	15*	Report numbers of outcome events or summary measures [tables 2-3]
Main results	16	(*a*) Give unadjusted estimates and, if applicable, confounder-adjusted estimates and their precision (eg, 95% confidence interval). Make clear which confounders were adjusted for and why they were included [figures 1-4, appendix 2, pages 6-7]
		(*b*) Report category boundaries when continuous variables were categorized [throughout tables]
		(*c*) If relevant, consider translating estimates of relative risk into absolute risk for a meaningful time period [n/a]
Other analyses	17	Report other analyses done—eg analyses of subgroups and interactions, and sensitivity analyses [pages 6-7]
**Discussion**		
Key results	18	Summarise key results with reference to study objectives [page 7]
Limitations	19	Discuss limitations of the study, taking into account sources of potential bias or imprecision. Discuss both direction and magnitude of any potential bias [page 7]
Interpretation	20	Give a cautious overall interpretation of results considering objectives, limitations, multiplicity of analyses, results from similar studies, and other relevant evidence [page 8]
Generalisability	21	Discuss the generalisability (external validity) of the study results [page 7]
**Other information**		
Funding	22	Give the source of funding and the role of the funders for the present study and, if applicable, for the original study on which the present article is based [page 3]

For peer review only
